# Predictors of Multiple Sclerosis After Clinically Isolated Syndrome: A Systematic Review and Meta‐Analysis

**DOI:** 10.1111/ene.70711

**Published:** 2026-07-31

**Authors:** María Paula Zafra‐Sierra, Carolina Ferreira‐Atuesta, Daniela Sofía Rodríguez, Jairo Gaitán, Susana Otero, Mar Tintoré, Fred Lublin, Gavin Giovannoni, Jaime Toro, Saúl Reyes

**Affiliations:** ^1^ Department of Neurology Fundación Santa Fe de Bogotá Bogotá Colombia; ^2^ School of Medicine Universidad El Bosque Bogotá Colombia; ^3^ Department of Neurology University Hospital Zurich Zurich Switzerland; ^4^ School of Medicine Universidad de los Andes Bogotá Colombia; ^5^ Multiple Sclerosis Centre of Catalonia (Cemcat) Vall D'hebron Barcelona Hospital Barcelona Spain; ^6^ The Corinne Goldsmith Dickinson Center for Multiple Sclerosis and Department of Neurology Icahn School of Medicine at Mount Sinai New York New York USA; ^7^ Blizard Institute, Faculty of Medicine and Dentistry Queen Mary University of London London UK

**Keywords:** clinically isolated syndrome, multiple sclerosis, progression, risk factors

## Abstract

**Background:**

Most people with multiple sclerosis (MS) present with a clinically isolated syndrome (CIS); however, not all individuals with CIS are subsequently diagnosed with MS.

**Methods:**

A systematic literature review was conducted until December 2025. Observational studies of adults with CIS that were later diagnosed with MS were included. Odds ratios (ORs) were pooled using random‐effects meta‐analysis. Heterogeneity was assessed with I^2^, sensitivity analyses with leave‐one‐out procedures, and publication bias with funnel plots and Egger's test. Meta‐regression was performed to explore further sources of heterogeneity when appropriate.

**Results:**

Seventy‐two studies with 9915 adults were included. In the meta‐analysis, younger age (OR = 1.6, *p* < 0.01) and multifocal presentation (OR = 1.55, *p* = 0.02) were associated with a diagnosis of MS after a CIS. Magnetic resonance imaging findings, including a higher number of T2 lesions (OR = 7.46, *p* = 0.02), periventricular lesions (OR = 4.08, *p* < 0.01), corpus callosum lesions (OR = 14.89, *p* = 0.02), infratentorial lesions (OR = 2.16, *p* = 0.03), spinal cord lesions (OR = 1.4, *p* = 0.03), and gadolinium‐enhancing lesions (OR = 1.91, *p* = 0.01), as well as cerebrospinal fluid inflammatory markers such as oligoclonal bands (OR = 3.57, *p* < 0.01) and pleocytosis (OR = 3.34, *p* = 0.02), were also associated with a subsequent diagnosis of MS.

**Conclusions:**

This meta‐analysis identified factors associated with an increased likelihood of MS after a CIS. These findings may help identify high‐risk individuals and guide personalized treatment strategies.

## Graphical abstract

Most people with multiple sclerosis (MS) present with a clinically isolated syndrome (CIS); however, not all individuals with CIS are subsequently diagnosed with MS. We identified several clinical (younger age and monofocal presentation), imaging (number of T2 lesions, lesion location, gadolinium enhancement), and paraclinical (oligoclonal bands and pleocytosis) predictors that predict higher odds of MS diagnosis after a first demyelinating event. Recognizing these predictors may stratify high‐risk patients and guide personalized treatment strategies.

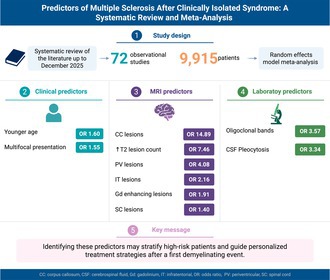

## 1 Introduction

Multiple sclerosis (MS) is the most common demyelinating disease of the central nervous system (CNS) in young adults, accounting for a high number of disability‐adjusted life years [1]. Approximately 85% of people with MS report having had a clinically isolated syndrome (CIS) event. A CIS event is defined as the first clinically symptomatic demyelinating event not yet fulfilling the MS diagnostic criteria [2]. Rates of MS diagnosis following a CIS vary widely, with estimates ranging from 40% to 85% over a 15–20‐year period; however, approximately 15% to 20% of cases do not meet diagnostic criteria during follow‐up [3].

Studies have shown that individuals diagnosed with MS in the early years after a CIS event are at high risk of increased inflammatory activity, poor recovery of neurological function, and pronounced brain atrophy [4]. Additionally, it is well established that the impact of disease‐modifying therapies (DMTs) is greatest in the earliest stages of the disease [5]. Given the prognostic variability and the importance of prompt intervention after CIS, early risk stratification is crucial for identifying high‐risk individuals and guiding treatment decisions. While oligoclonal bands (OCBs) and abnormal MRI findings are well‐established predictors of MS diagnosis after CIS, the role of other factors, such as demographic characteristics and additional biomarkers, remains unclear. To date, no comprehensive systematic evaluation of these factors has been conducted. This study used a systematic review and meta‐analysis to identify the factors associated with an increased likelihood of MS after a CIS.

## 2 Methods

This study was conducted following the Preferred Reporting Items for Systematic Reviews and Meta‐analyses (PRISMA) reporting guidelines [6]. This work was registered and the protocol published in PROSPERO CRD42022324386. No ethics committee approval was required as this study uses previously published data.

### 2.1 Search Strategy and Study Selection

A systematic review of the literature in EMBASE, PubMed, and Scopus was conducted up to December 31st, 2025, using a predefined search strategy (eAppendix 1 in *Supplement*). Observational studies of adults with a history of a CIS that reported subsequent MS diagnosis status were included, encompassing cohort, cross‐sectional, and case–control designs. Studies were excluded if the full text was unavailable, outcome data were missing, odds ratios (ORs) were not reported and raw data were unavailable for calculation, or if they consisted of duplicate publications, reviews, conference proceedings, dissertations, ongoing research, or preprints, or were published in languages other than Spanish or English. Four investigators screened studies according to title and abstract (M.P.Z., J.G., D.S.R., S.R.N.), and then a full‐text review was done. A third author resolved disagreements between the two reviewing authors selected for each article, and senior authors addressed unresolved discrepancies. Covidence [7] software was used for study screening and selection.

### 2.2 Definitions and Outcomes

A CIS was defined as a first clinical episode in which a patient has symptoms and signs suggestive of an inflammatory demyelinating disorder of the CNS [2]. Monofocal CIS was defined as a CIS with signs indicating a lesion affecting one of the following regions: the optic nerve, spinal cord, brainstem or cerebellum, or less commonly in a cerebral hemisphere [2]. Multifocal CIS was defined as a clinically isolated syndrome involving ≥ 2 of these regions [2]. Given the evolution of MS diagnostic criteria over time [8, 9, 10, 11, 12, 13], MS diagnosis was defined according to the criteria used in each study. The primary outcome was a diagnosis of MS following a CIS event.

### 2.3 Data Extraction

Three authors (M.P.Z., J.G., D.S.R.) independently extracted data from all included studies. Collected information included the first author, year of publication, study title, criteria used for MS diagnosis, follow‐up duration in months, sample size, and the number of patients diagnosed with MS versus those not diagnosed after a CIS event. In addition, the following evaluated factors were recorded: age, sex, smoking status, type of CIS, baseline Expanded Disability Status Scale (EDSS) score, vitamin D levels, cerebrospinal fluid (CSF) findings such as pleocytosis, OCBs, and neurofilament light chain (NfL) levels, as well as baseline MRI characteristics, including the number of T2 lesions, gadolinium‐enhancing lesions, and lesion location in the periventricular area, infratentorial region, corpus callosum, and spinal cord. To ensure consistency, extracted data were cross‐checked, and discrepancies were resolved through discussion among the senior authors.

### 2.4 Risk‐Of‐Bias Assessment

Two authors (M.P.Z. and J.G.) independently evaluated the quality of the included studies using the Newcastle‐Ottawa Quality Assessment Scale [14]. Studies were assessed based on factors such as cohort representativeness, selection of non‐exposed groups, ascertainment of exposure, control for confounding variables, outcome assessment, follow‐up duration, and adequacy of follow‐up. Discrepancies in scoring were resolved through discussion between the senior authors.

### 2.5 Statistical Analysis

A random‐effects model meta‐analysis was conducted for evaluated factors with sufficient data from at least three cohort studies. ORs were extracted for each factor or calculated from raw data when available. Heterogeneity was assessed using Cochran's Q test and I^2^ statistics, categorized as low (< 25%), moderate (25%–75%), or high (> 75%). Knapp–Hartung adjustments were applied to calculate confidence intervals around the pooled effect. Sensitivity analysis and publication bias were assessed using leave‐one‐out influence analyses for all meta‐analytic models and Egger's regression test when at least 10 studies were available; otherwise, visual inspection of funnel plots was performed. The choice of additional sensitivity and bias analyses was guided by the degree of between‐study heterogeneity rather than the statistical significance of pooled effects. Accordingly, for outcomes with high heterogeneity, additional analyses were conducted, including meta‐regression to explore potential sources of variability (i.e., study design, diagnostic criteria, follow‐up duration, and study sample size). Statistical analyses were performed in R (version 4.4.2) using the meta, metafor, and dmetar packages. *p*‐values were two‐sided, with a significance level set at *p* < 0.05.

## 3 Results

### 3.1 Study Characteristics

**FIGURE 1:**
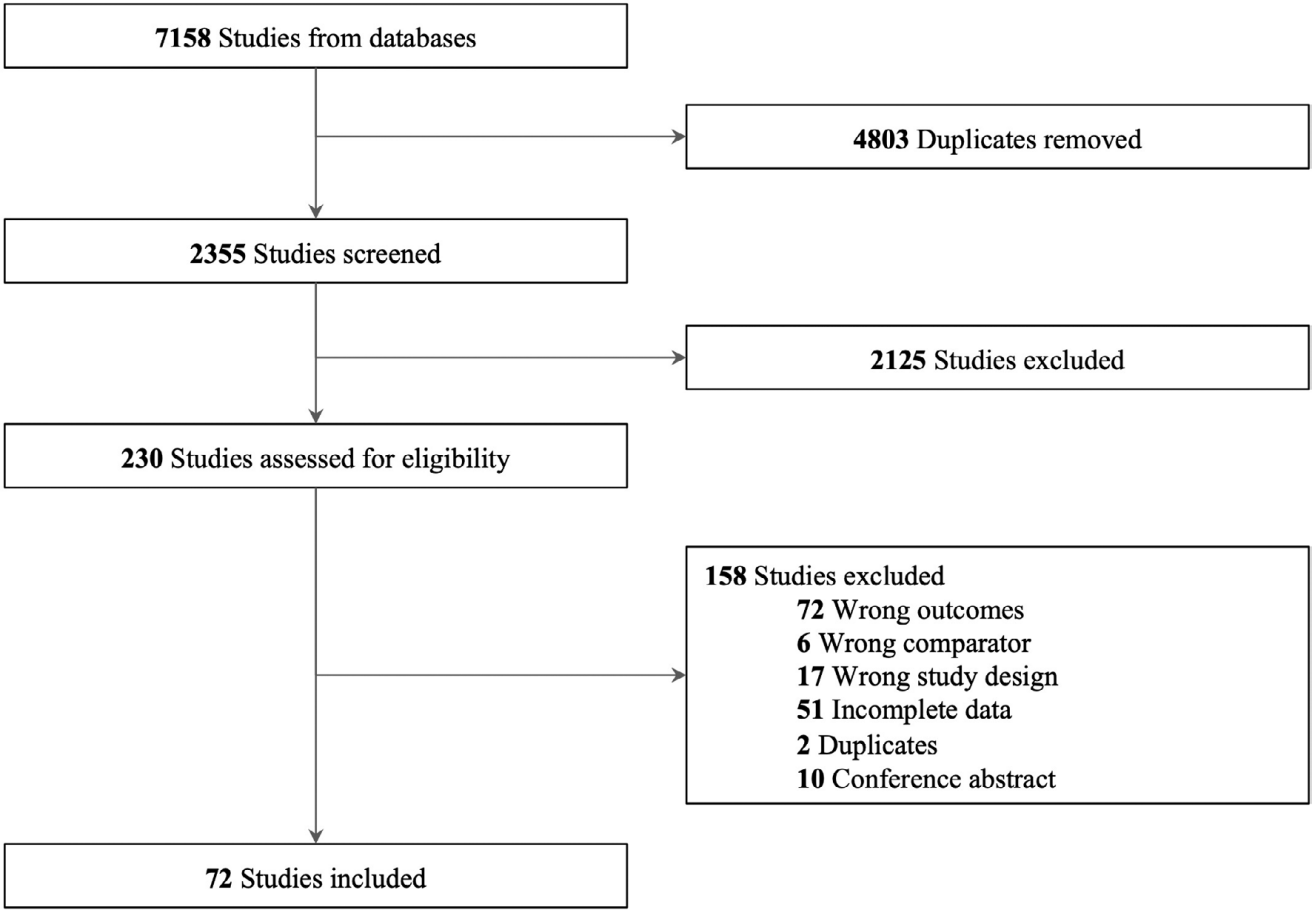
PRISMA diagram.

A total of 72 studies, comprising 9915 participants from 69 unique cohorts, were included in the meta‐analysis (Figure 1) [4, 15, 16, 17, 18, 19, 20, 21, 22, 23, 24, 25, 26, 27, 28, 29, 30, 31, 32, 33, 34, 35, 36, 37, 38, 39, 40, 41, 42, 43, 44, 45, 46, 47, 48, 49, 50, 51, 52, 53, 54, 55, 56, 57, 58, 59, 60, 61, 62, 63, 64, 65, 66, 67, 68, 69, 70, 71, 72, 73, 74, 75, 76, 77, 78, 79, 80, 81, 82, 83, 84, 85]. The median follow‐up duration for all studies was 37.5 months (interquartile range [IQR] 24–60). Fifty‐nine studies (81.9%) were prospective cohorts [4, 15, 16, 17, 18, 20, 21, 22, 23, 24, 25, 26, 28, 30, 31, 32, 33, 35, 36, 37, 38, 39, 40, 41, 42, 44, 45, 46, 48, 50, 51, 52, 54, 55, 56, 57, 58, 59, 60, 61, 62, 63, 64, 65, 66, 67, 70, 71, 72, 74, 75, 76, 78, 79, 80, 81, 82, 83, 84] and 47 (65.3%) were published in the last decade [15, 16, 17, 18, 19, 20, 21, 23, 24, 25, 26, 27, 28, 30, 31, 33, 35, 36, 37, 39, 40, 45, 46, 48, 50, 51, 53, 55, 57, 58, 59, 62, 63, 64, 65, 66, 69, 70, 71, 77, 78, 79, 80, 81, 82, 83]. The McDonald 2010 criteria [11] were applied in 25 studies (34.7%) [15, 16, 17, 21, 23, 28, 34, 35, 38, 45, 49, 55, 57, 58, 59, 61, 62, 63, 64, 65, 70, 79, 81, 82, 85], the Poser criteria [10] in 21 studies (29.2%) [18, 19, 24, 26, 29, 30, 39, 44, 46, 48, 53, 54, 56, 60, 66, 67, 69, 71, 74, 75, 76], the McDonald 2005 criteria [8] in 11 studies (15.3%) [4, 25, 31, 36, 37, 42, 50, 51, 52, 68, 84], the McDonald 2017 [12] criteria in 8 studies (11.1%) [20, 27, 33, 77, 78, 80, 83], and the McDonald 2001 [9] in 4 studies (5.6%) [22, 41, 47, 73]. Regarding year of publication, 1 study (1.4%) [60] was published before 2005, 8 studies (11.1%) [32, 41, 42, 47, 56, 68, 74, 76] between 2006 and 2010, 33 studies (45.8%) [4, 22, 24, 25, 28, 29, 30, 31, 34, 35, 36, 37, 38, 39, 43, 44, 48, 49, 50, 51, 52, 54, 55, 61, 66, 67, 69, 70, 71, 72, 73, 84, 85] between 2011 and 2017, and 30 studies (41.7%) [15, 16, 17, 18, 19, 20, 21, 23, 26, 27, 33, 40, 45, 46, 53, 57, 58, 59, 62, 63, 64, 65, 75, 77, 78, 79, 80, 81, 82, 83] between 2018 and 2024. The studies covered 26 countries, including Spain (*n* = 11, 15.3%) [21, 24, 25, 47, 50, 54, 60, 61, 70, 71, 76], Italy (*n* = 10, 13.9%) [22, 28, 29, 36, 40, 49, 52, 62, 69, 74], Germany (*n* = 5, 6.9%) [35, 37, 42, 45, 68], Czech Republic (*n* = 5, 6.9%) [4, 15, 19, 46, 83], China (*n* = 4, 5.6%) [27, 44, 53, 80], and the United Kingdom (*n* = 4, 5.46%) [18, 32, 58, 81]. Details of all assessed studies are available in the eTable 1 and eTable 2 of the *Supplement*.

### 3.2 Risk of Bias

The risk of bias analysis revealed that 44 studies (61.1%) were classified as good quality [4, 15, 17, 22, 23, 25, 26, 27, 28, 29, 31, 32, 34, 38, 39, 41, 43, 47, 49, 50, 52, 53, 54, 55, 56, 57, 58, 61, 63, 66, 67, 68, 70, 71, 73, 74, 76, 77, 78, 80, 81, 82, 84, 85], and 26 studies (36.1%) were considered poor quality (eTable 3 and eFigure 1 in *Supplement*) [16, 18, 19, 20, 21, 24, 33, 35, 36, 37, 40, 42, 44, 45, 46, 48, 51, 59, 60, 62, 64, 65, 72, 79, 83, 86]. Methodological concerns were observed in the domains of outcomes and comparability, due to insufficient follow‐up (*n* = 55, 76.4%) [4, 15, 16, 17, 19, 20, 21, 22, 23, 24, 28, 29, 30, 31, 33, 34, 35, 36, 37, 38, 39, 40, 41, 42, 43, 44, 45, 46, 47, 49, 52, 53, 55, 56, 57, 59, 60, 61, 62, 63, 64, 65, 66, 67, 68, 69, 72, 73, 74, 77, 78, 79, 80, 83, 84] and due to lack of confounder adjustment (*n* = 37, 51.4%) [16, 18, 19, 20, 21, 23, 24, 29, 30, 31, 33, 35, 36, 37, 40, 42, 44, 45, 46, 48, 51, 54, 59, 60, 61, 62, 63, 64, 65, 69, 72, 75, 79, 81, 82, 83, 85], respectively.

### 3.3 Meta‐Analysis Results

#### 3.3.1 Demographic and Clinical Variables

**FIGURE 2:**
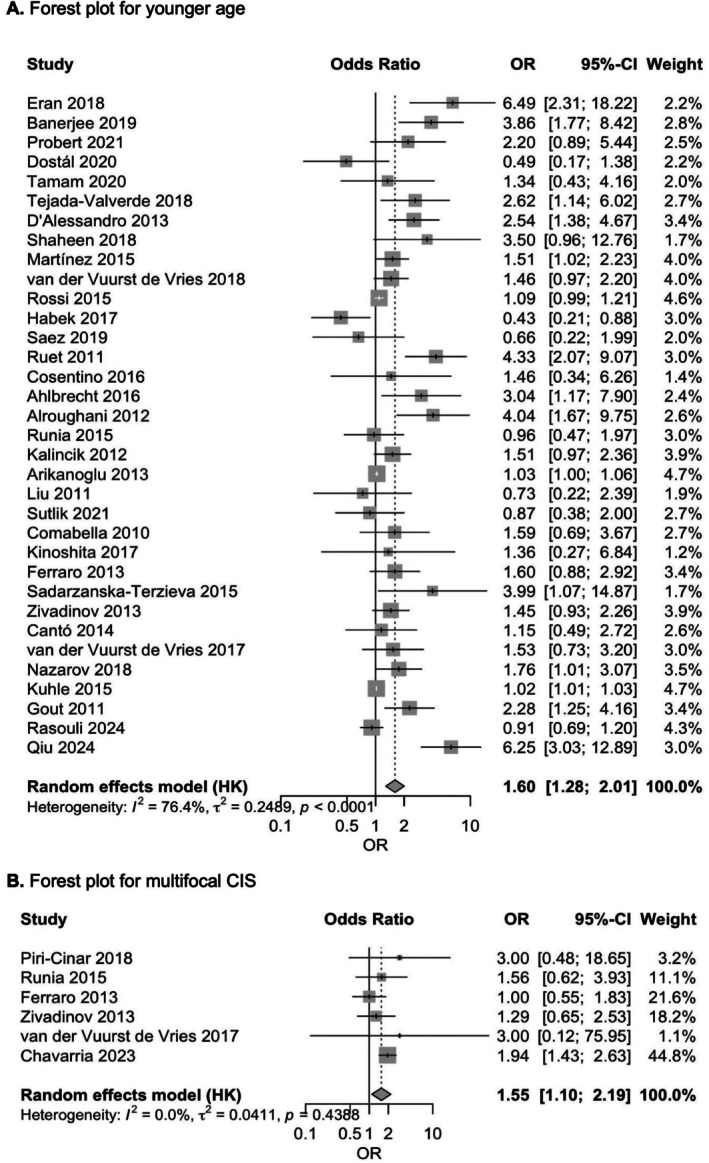
Forest plot for (A) younger age and (B) multifocal clinically isolated syndrome (CIS).

A total of 34 studies, including 4598 patients [4, 16, 17, 18, 19, 20, 21, 22, 25, 26, 28, 30, 33, 34, 36, 37, 38, 39, 43, 44, 46, 47, 48, 49, 51, 54, 55, 59, 66, 67, 79, 80, 84, 87], examined the association between younger age at CIS onset and the likelihood of a subsequent diagnosis of MS. A statistically significant association was observed (OR = 1.6, 95% CI 1.30–2.00, *p* = 0.0001), with high heterogeneity (I^2^ = 76.4%) (Table 1, Figure 2). Meta‐regression analyses including study design, diagnostic criteria used, follow‐up duration, and sample size did not identify significant moderators (eTable 4). The leave‐one‐out sensitivity analysis further confirmed robustness (eFigure 2). The funnel plot appeared asymmetric and Egger's test was significant (*p* < 0.0001) (eFigure 3); however, no association between study size and effect magnitude was observed in the meta‐regression.

**TABLE 1:** Meta‐analysis results.

Variable	Meta‐analysis					
	Number of studies	OR	IC 95%	*p*‐value	I^2	Eggers test *p*‐value
**Age**						
Younger age	34	1.6044	1.2823–2.0075	0.0001	76.4%	< 0.0001
**Sex**						
Female	39	1.0656	0.8805–1.2896	0.5044	35.8%	0.0138
**EDSS**						
Greater score	16	1.9639	0.7025–5.4903	0.1820	80.8%	0.0940
**Type of CIS**						
Optic neuritis	13	0.7112	0.4054–1.2475	0.2110	67.9%	0.9271
Spinal cord syndrome	13	1.2951	0.6677–2.5120	0.4118	63.5%	0.5985
Multifocal	6	1.5517	1.1014–2.1862	0.0216	0%	N/A
**Smoking status**						
Smoker	3	1.6073	0.2753–9.3836	0.3667	88.1%	N/A
**MRI characteristics**						
Greater number of T2 lesions	10	7.4605	1.5277–36.4343	0.0186	93.1%	0.0118
Spinal cord lesions	7	1.3713	1.0501–1.7907	0.0275	32.3%	N/A
Periventricular lesions	10	4.0826	2.9920–5.5708	< 0.0001	30%	0.1752
Infratentorial lesions	7	2.1625	1.0986–4.2564	0.0317	61.8%	N/A
Corpus callosum lesions	3	14.8848	3.4218–64.7489	0.0156	0%	N/A
Gadollinium enhancement	9	1.9117	1.2623–2.8954	0.0070	0%	N/A
**Biomarkers**						
Vitamind D deficiency	3	7.2435	0.3580–146.5540	0.1053	84.7%	N/A
Oligoclonal bands	38	3.5671	2.7225–4.6737	< 0.0001	65.2%	< 0.0001
CSF NFL	4	4.3032	0.6824–27.1367	0.0860	84.9%	N/A
CSF pleocytosis	6	3.3874	1.3759–8.3393	0.0176	75.6%	N/A

No significant associations were observed for sex, baseline EDSS scores, or smoking status (Table 1). The analysis of sex showed low‐to‐moderate heterogeneity, and EDSS scores and smoking status demonstrated substantial between‐study heterogeneity. The leave‐one‐out sensitivity analyses did not identify influential studies for sex or EDSS scores (eFigure 4); the analysis of smoking status showed marked instability due to the small number of available studies. Egger's test did not indicate significant funnel plot asymmetry for EDSS scores, and results for sex suggested possible asymmetry in the absence of a statistically significant pooled effect (eFigure 5).

Six studies, including 939 patients, evaluated multifocal presentation at CIS onset [23, 39, 49, 55, 82, 84]. Multifocal onset was significantly associated with MS diagnosis (OR = 1.55, 95% CI 1.10–2.19, *p* = 0.022) (Table 1, Figure 2). No relevant between‐study heterogeneity was observed (I^2^ = 0%). The leave‐one‐out analysis showed similar effect estimates across models (OR range approximately 1.26–1.81); statistical significance was attenuated when the largest study was excluded (eFigure 6). Formal assessment of funnel plot asymmetry was not performed due to the limited number of studies (eFigure 7). Other clinical presentation features, including optic neuritis and spinal cord syndrome, were not significantly associated with a subsequent diagnosis of MS (Table 1). These analyses showed moderate‐to‐substantial between‐study heterogeneity. Leave‐one‐out sensitivity analyses demonstrated consistent effect estimates across models, with no single study materially influencing the pooled results, and Egger's tests did not suggest funnel plot asymmetry (eFigures 8 and 9).

#### 3.3.2 MRI Variables

**FIGURE 3:**
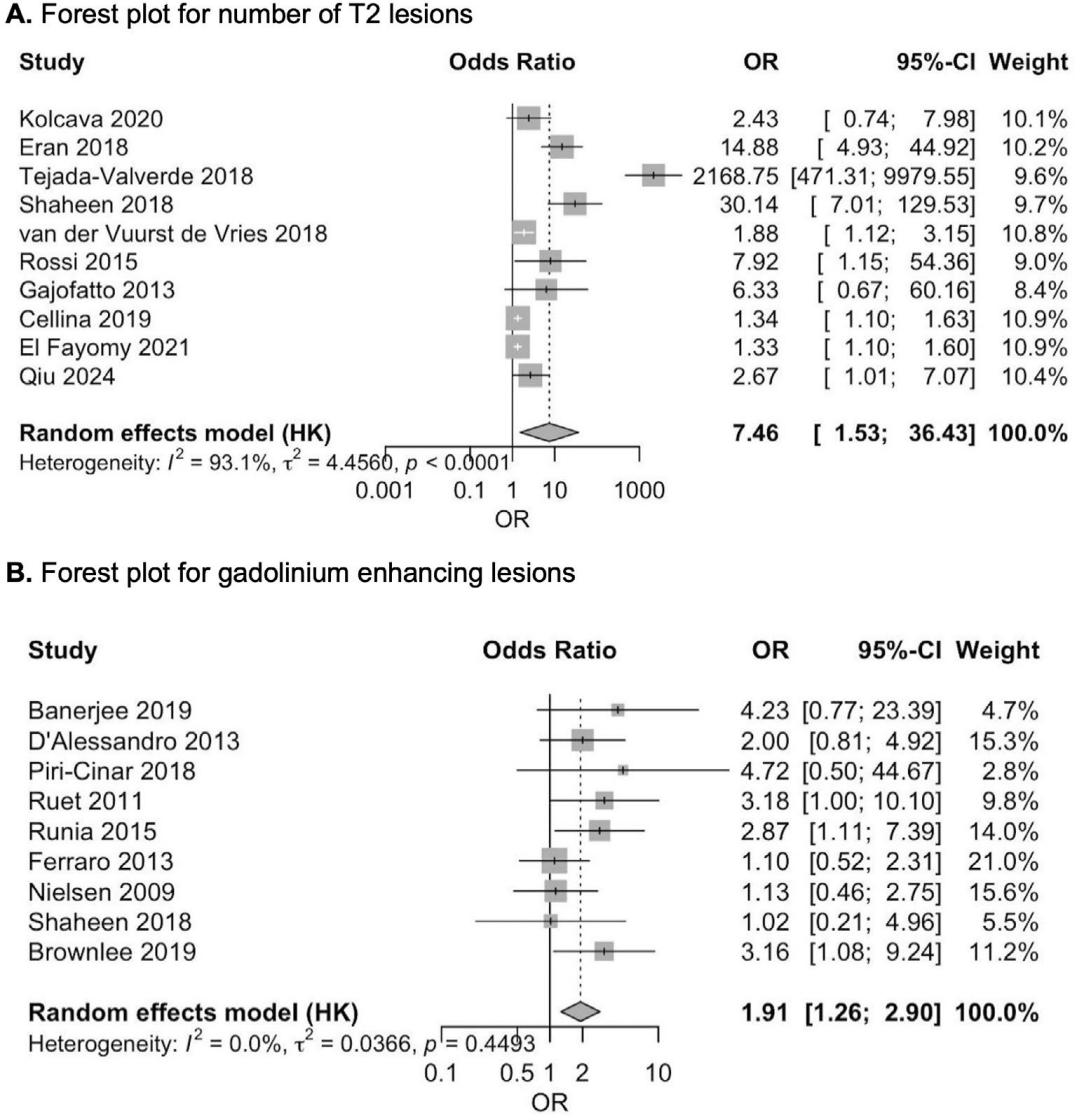
Forest plot for (A) number of T2 lesions and (B) gadolinium enhancing lesions.

Ten studies, including 731 patients, evaluated the association between the number of T2 lesions at CIS onset and a subsequent MS diagnosis [15, 16, 21, 26, 28, 29, 40, 63, 80, 87]. A higher T2 lesion burden was significantly associated with MS (OR = 7.46, 95% CI 1.53–36.43, *p* = 0.019) (Table 1, Figure 3). Between‐study heterogeneity was substantial (I^2^ = 93.1%). Leave‐one‐out sensitivity analyses showed consistent associations across models (eFigure 10). Visual inspection of the funnel plot and Egger's test suggested possible small‐study effects (eFigure 11). Meta‐regression analyses including MS diagnostic criteria and study design did not identify significant sources of heterogeneity (eTable 5).

**FIGURE 4:**
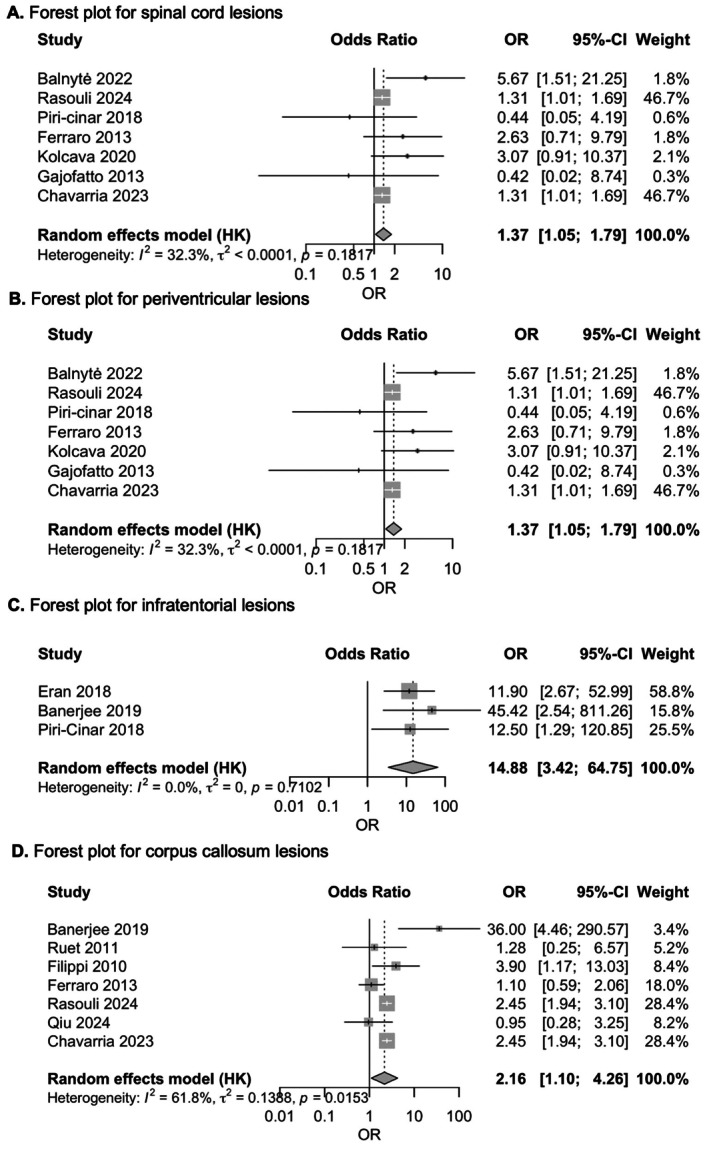
Forest plot for lesion localization on MRI: (A) spinal cord lesions, (B) periventricular lesions, (C) infratentorial lesions, and (D) corpus callosum lesions.

The location of MRI lesions at CIS onset was associated with an increased risk of a subsequent diagnosis of MS (Table 1, Figure 4): Periventricular lesions (10 studies, 1232 patients) [16, 17, 23, 29, 32, 34, 74, 79, 80, 82] showed a significant association with MS diagnosis (OR = 4.08, 95% CI 2.99–5.57, *p* < 0.0001; I^2^ = 30.0%), with no evidence of small‐study effects on Egger's test (*p* = 0.18). Corpus callosum lesions (3 studies, 169 patients) [16, 17, 23] were also associated with MS (OR = 14.88, 95% CI 3.42–64.75, *p* = 0.0156; I^2^ = 0%). Similarly, significant associations with MS were observed for infratentorial lesions (7 studies, 1545 patients) [17, 34, 49, 74, 79, 80, 82] (OR = 2.16, 95% CI 1.10–4.26, *p* = 0.0317; I^2^ = 61.8%), and spinal cord lesions (7 studies, 1040 patients) [15, 23, 29, 49, 77, 79, 82] (OR = 1.37, 95% CI 1.05–1.79, *p* = 0.0275; I^2^ = 32.3%). Visual inspection of funnel plots for all the analyses did not suggest marked asymmetry, and leave‐one‐out sensitivity analyses showed that no single study had a dominant influence on the pooled estimates (eFigures 12 and 13).

Gadolinium‐enhancing lesions were associated with an increased likelihood of MS diagnosis (9 studies, 1149 patients, OR = 1.91, 95% CI 1.26–2.90, *p* = 0.007) (Table 1, Figure 3) [17, 22, 23, 34, 39, 49, 56, 57, 58], with low heterogeneity (I^2^ = 0%). Sensitivity analyses using the leave‐one‐out approach showed consistent results (eFigure 14), and visual inspection of the funnel plot did not suggest relevant asymmetry (eFigure 15).

#### 3.3.3 Biomarkers

**FIGURE 5:**
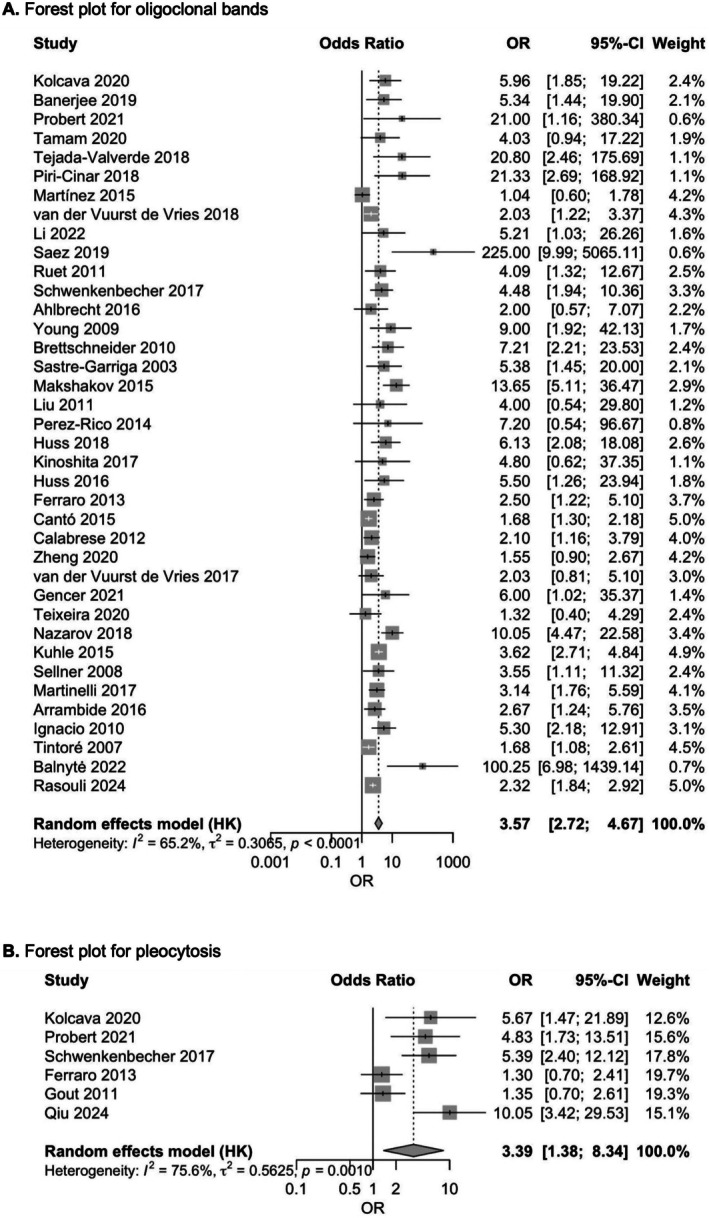
Forest plot for (A) oligoclonal bands and (B) cerebrospinal fluid pleocytosis.

The presence of OCBs was associated with an increased risk of MS diagnosis (38 studies, 6066 patients; OR = 3.57, 95% CI 2.72–4.67, *p* < 0.0001), with moderate heterogeneity (I^2^ = 65.2%) (Table 1, Figure 5) [15, 17, 18, 20, 21, 23, 25, 26, 27, 33, 34, 35, 37, 41, 42, 44, 45, 48, 49, 50, 52, 53, 55, 59, 60, 61, 64, 65, 66, 68, 69, 70, 75, 76, 77, 79, 85]. Leave‐one‐out sensitivity analyses showed consistent results (eFigure 16), and Egger's test suggested the presence of small‐study effects (*p* < 0.0001) (eFigure 17). CSF pleocytosis was also significantly associated with a subsequent diagnosis of MS (6 studies, 747 patients; OR = 3.39, 95% CI 1.38–8.34, *p* = 0.0176), with substantial heterogeneity (I^2^ = 75.6%) (Table 1, Figure 5) [15, 18, 35, 49, 67, 80]. Leave‐one‐out sensitivity analyses showed no single study driving the overall effect (eFigure 18), and visual inspection of the funnel plot did not reveal marked asymmetry (eFigure 19).

Vitamin D and CSF NfL levels were evaluated in a limited number of studies and were not significantly associated with MS (Table 1). Both analyses showed substantial heterogeneity and unstable pooled estimates, with leave‐one‐out sensitivity analyses indicating variability in the overall effect when individual studies were excluded (eFigure 20).

## 4 Discussion

This systematic review and meta‐analysis identified factors associated with an increased likelihood of MS diagnosis following a CIS event. Younger age at onset, multifocal clinical presentation, higher MRI lesion burden, specific lesion locations (periventricular, corpus callosum, infratentorial, and spinal cord), gadolinium‐enhancing lesions, and the presence of OCBs and CSF pleocytosis emerged as predictors, supporting their role in early risk stratification.

The increased likelihood of MS diagnosis among patients with younger age at CIS onset appeared consistent across study designs and clinical contexts. Previous studies in children and adolescents have shown that younger individuals with CIS have the highest rates of MS and the shortest time to diagnosis, consistent with a more inflammatory disease course and higher annualized relapse rates [88]. Similarly, in adults, younger age at CIS onset has also been associated with a higher likelihood of being diagnosed with MS, particularly when baseline MRI demonstrates dissemination in space (DIS) [89]. With advancing age, there is a marked reduction in inflammatory disease activity, including fewer relapses and a decrease in contrast‐enhancing lesions and new T2 lesions on MRI [90]. This reduction may be attributed, among other factors, to immunosenescence [91]. Taken together, these findings suggest that younger patients with CIS may experience a more active inflammatory process, potentially contributing to earlier disease progression and a higher likelihood of MS diagnosis.

The association between multifocal presentation at CIS onset and MS diagnosis is biologically plausible and consistent with diagnostic criteria, as multifocality may reflect broader dissemination of demyelinating activity within the CNS. Multifocal presentations have been associated with a higher risk of a second clinical attack, a higher baseline lesion burden, and a greater likelihood of early disability progression [92]. Furthermore, patients with multifocal CIS may already fulfill DIS criteria, a highly specific marker for MS [13]. Recent evidence suggests that patients fulfilling classic lesion distribution criteria may not require prolonged follow‐up to establish dissemination in time (DIT) [93]. Accordingly, DIT is not mandatory for a diagnosis of MS in specific situations under the 2024 revisions of the McDonald criteria [13].

MRI markers also play a central role in MS risk prediction. Our results showed that a higher number of T2 lesions in patients with CIS is associated with an increased likelihood of MS diagnosis. This association has been further characterized in previous studies, which have shown that patients with 10 or more T2 lesions are at particularly high risk of a second attack and subsequent MS diagnosis [94]. T2 lesions are widely recognized as markers of disease activity, and their accumulation over time may contribute to ongoing axonal damage and disability [94]. Similarly, T2 lesion volume and the extent of intralesional myelin loss are predictive of disease progression and burden [95]. These findings suggest that patients with CIS who have a higher number of T2 lesions may experience a more aggressive disease course, thereby increasing their likelihood of fulfilling diagnostic criteria for MS. Importantly, although the Egger's test was not significant, the funnel plot showed marked asymmetry, suggesting potential small‐study effects and possible overestimation of the association.

Several factors may account for the association between specific lesion locations and subsequent MS diagnosis. Periventricular lesions are a hallmark of MS pathology, and their detection is a key component of the DIS criteria, thereby increasing the likelihood of meeting diagnostic requirements [13]. Periventricular lesions are not specific to MS and can be seen in different conditions (e.g., normal aging, vascular comorbidities, and migraine); however, requiring ≥ 3 periventricular lesions in older patients with CIS improves diagnostic specificity for MS compared with a single lesion [96]. Furthermore, periventricular lesions are not only diagnostic but also prognostic and have been associated with greater tissue damage and disability worsening [97]. Corpus callosum lesions were also identified as a high‐risk feature, consistent with previous evidence linking corpus callosum involvement with subsequent clinical relapses and disability progression [98]. In addition, studies using advanced MRI techniques indicate that structural and microstructural changes in the corpus callosum are present early in CIS, reflecting underlying myelin pathology [99]. Although corpus callosum lesions can be seen in other inflammatory diseases such as neuromyelitis optica spectrum disorder and acute demyelinating encephalomyelitis, some lesion features (i.e., focal, ovoid, with clear margins, and radially oriented) are more characteristic of MS [100]. The association between infratentorial lesions and subsequent MS diagnosis is supported by previous studies demonstrating that the presence of ≥ 1 infratentorial lesion at CIS onset confers a significantly increased risk of MS, with brainstem involvement carrying particularly strong prognostic implications [101]. Furthermore, multiple infratentorial lesions have been associated with both a higher likelihood of diagnosis and greater disability accumulation over time [101]. These findings underscore the prognostic relevance of infratentorial involvement in early CIS. Finally, spinal cord lesions are likewise predictors of MS diagnosis in CIS, and the presence of multiple lesions in this location further strengthens this association [102]. Spinal involvement has been linked to a shorter time to MS diagnosis and disability progression [103]. Notably, the contribution of spinal cord lesions to MS diagnosis appears to be particularly evident in patients with CIS who do not meet brain MRI criteria [103]. This association may be partially attributed to the high specificity of spinal cord lesions for MS, and to their potential to reflect a more aggressive disease phenotype [104].

Gadolinium‐enhancing lesions were significantly associated with MS diagnosis and showed minimal heterogeneity, supporting their role as markers of active inflammation and blood–brain barrier disruption. Additionally, their presence may contribute to continued demyelination and axonal damage [89]. Evidence suggests that even before the onset of clinical symptoms (i.e., radiologically isolated syndrome), gadolinium‐enhancing lesions are linked to a higher likelihood of MS diagnosis over time [105]. However, it is important to note that these lesions are observed in only one‐third of patients with a first clinical event, limiting their sensitivity as a diagnostic tool [106].

Our findings align with growing evidence that the presence of CSF OCBs significantly increases the probability of MS diagnosis in CIS patients, consistently showing more than a twofold increase in likelihood and a shorter time to diagnosis [35, 86]. Importantly, the likelihood of MS diagnosis in CIS is influenced not only by the presence of OCBs but also by their number in the CSF, independently of brain MRI lesion burden [107]. A possible explanation is the role of OCBs as markers of an ongoing immune response within the CNS, suggesting a higher likelihood of disease progression [108]. Moreover, the role of OCBs in diagnosing MS has long been established. The 2017 McDonald criteria explicitly incorporate the presence of CSF‐specific OCBs as a marker of DIT, allowing for an MS diagnosis in patients with a typical CIS and MRI evidence of DIS [12]. Of note, their specificity can be limited, underscoring the need for complementary biomarkers to enhance diagnostic accuracy [109]. Interestingly, CSF pleocytosis, mostly defined as mild, was also associated with a subsequent diagnosis of MS. This is consistent with previous studies showing that pleocytosis can occur in ≥ 50% of patients with CIS in the context of MS [110]. Furthermore, CSF pleocytosis may reflect a more aggressive inflammatory milieu and has been associated with short‐term inflammatory disease activity and acute lesions with restricted diffusion on MRI [111].

### 4.1 Strengths and Limitations

This meta‐analysis provides a comprehensive evaluation of factors associated with MS diagnosis after CIS, an area not previously examined in a unified and systematic manner, supported by a rigorous methodology, an inclusive search strategy, and a large sample of patients with CIS. Most included studies were prospective cohorts, thereby reducing the risk of selection and recall bias. Sensitivity analyses using a leave‐one‐out approach confirmed that no single study unduly influenced the pooled estimates, supporting the robustness of the findings.

Several limitations should be acknowledged. Substantial heterogeneity was observed across multiple analyses, likely reflecting differences in patient populations, follow‐up duration, MRI protocols, and biomarker assays. Although meta‐regression and sensitivity analyses were performed, residual heterogeneity persisted. Furthermore, the evolution of MS diagnostic criteria over time, particularly the increased sensitivity of the 2017 McDonald criteria, may have influenced MS diagnosis rates and limited direct comparability across studies. Finally, disparities in healthcare systems and access to diagnostic resources across regions may have affected the time to diagnosis and the likelihood of MS confirmation, factors not fully captured in the available data.

This systematic review and meta‐analysis provide comprehensive evidence on factors associated with MS diagnosis following a CIS event. Younger age at onset, multifocal clinical presentation, MRI features of disease burden, and CSF inflammatory biomarkers were consistently associated with an increased likelihood of MS diagnosis. Our findings provide valuable insights into the early and accurate risk stratification of patients with CIS. This is highly relevant in the context of the current era of MS management, which focuses on early and effective treatment to prevent further disease activity and neurological disability.

## Author Contributions

**Carolina Ferreira‐Atuesta:** conceptualization, investigation, writing – original draft, methodology, writing – review and editing. **Susana Otero:** conceptualization, writing – review and editing, supervision. **María Paula Zafra‐Sierra:** conceptualization, investigation, writing – original draft, methodology, writing – review and editing, data curation, formal analysis. **Mar Tintoré:** conceptualization, writing – review and editing, supervision. **Gavin Giovannoni:** conceptualization, writing – review and editing, supervision. **Jaime Toro:** conceptualization, writing – review and editing, supervision. **Jairo Gaitán:** conceptualization, investigation, writing – review and editing. **Daniela Sofía Rodríguez:** conceptualization, investigation, writing – review and editing. **Fred Lublin:** conceptualization, writing – review and editing, supervision. **Saúl Reyes:** conceptualization, investigation, writing – original draft, methodology, writing – review and editing, supervision.

## Funding

The authors have nothing to report.

## Disclosure

The authors have nothing to report.

## Conflicts of Interest

The authors declare no conflicts of interest.

## Supporting information


**Data S1:** eAppendix 1. Search strategy.
eTable 1. Studies included in the systematic review.
eTable 2. Studies assessed but not included in the systematic review.
eTable 3. Risk of bias assessment.
eTable 4. Meta‐regression for age.
eTable 5. Meta‐regression for number of T2 lesions.
eFigure 1. Risk of bias assessment summary plot.
eFigure 2. Forest‐plot of leave‐one‐out analysis for younger age.
eFigure 3. Funnel plot for younger age.
eFigure 4. Forest‐plot of leave‐one‐out analysis for female sex and EDSS.
eFigure 5. Funnel plot for female sex and EDSS.
eFigure 6. Forest‐plot of leave‐one‐out analysis for multifocal presentation.
eFigure 7. Funnel plot for multifocal presentation.
eFigure 8. Forest‐plot of leave‐one‐out analysis for optic neuritis and spinal cord.
eFigure 9. Funnel plot for optic neuritis and spinal cord.
eFigure 10. Forest‐plot of leave‐one‐out analysis for number of T2 lesions.
eFigure 11. Funnel plot for number of T2 lesions.
eFigure 12. Forest‐plot of leave‐one‐out analysis for lesion localization.
eFigure 13. Funnel plot for lesion localization.
eFigure 14. Forest‐plot of leave‐one‐out analysis for gadolinium enhancement.
eFigure 15. Funnel plot for gadolinium enhancement.
eFigure 16. Forest‐plot of leave‐one‐out analysis for oligoclonal bands.
eFigure 17. Funnel plot for oligoclonal bands.
eFigure 18. Forest‐plot of leave‐one‐out analysis for CSF pleocytosis.
eFigure 19. Funnel plot for CSF pleocytosis.
eFigure 20. Forest‐plot of leave‐one‐out analysis for serum vitamin D deficiency and CSF NFL.

## Data Availability

The data that support the findings of this study are available from the corresponding author upon reasonable request.
