# Supplementary material for: Predictors of Multiple Sclerosis After Clinically Isolated Syndrome: A Systematic Review and Meta‐Analysis
Source: Eur J Neurol. 2026 Jul 31;33(8):e70711. doi: 10.1111/ene.70711 (PMC13428175; doi:10.1111/ene.70711)
Supplement: Supplementary file 1 — Data S1: eAppendix 1. Search strategy. eTable 1. Studies included in the systematic review. eTable 2. Studies assessed but not included in the systematic review. eTable 3. Risk of bias assessment. eTable 4. Meta‐regression for age. eTable 5. Meta‐regression for number of T2 lesions. eFigure 1. Risk of bias assessment summary plot. eFigure 2. Forest‐plot of leave‐one‐out analysis for younger age. eFigure 3. Funnel plot for younger age. eFigure 4. Forest‐plot of leave‐one‐out analysis for female sex and EDSS. eFigure 5. Funnel plot for female sex and EDSS. eFigure 6. Forest‐plot of leave‐one‐out analysis for multifocal presentation. eFigure 7. Funnel plot for multifocal presentation. eFigure 8. Forest‐plot of leave‐one‐out analysis for optic neuritis and spinal cord. eFigure 9. Funnel plot for optic neuritis and spinal cord. eFigure 10. Forest‐plot of leave‐one‐out analysis for number of T2 lesions. eFigure 11. Funnel plot for number of T2 lesions. eFigure 12. Forest‐plot of leave‐one‐out analysis for lesion localization. eFigure 13. Funnel plot for lesion localization. eFigure 14. Forest‐plot of leave‐one‐out analysis for gadolinium enhancement. eFigure 15. Funnel plot for gadolinium enhancement. eFigure 16. Forest‐plot of leave‐one‐out analysis for oligoclonal bands. eFigure 17. Funnel plot for oligoclonal bands. eFigure 18. Forest‐plot of leave‐one‐out analysis for CSF pleocytosis. eFigure 19. Funnel plot for CSF pleocytosis. eFigure 20. Forest‐plot of leave‐one‐out analysis for serum vitamin D deficiency and CSF NFL. [file ENE-33-e70711-s001.docx]

**Supplementary Online Content**

**eAppendix 1.** Search strategy

**eTable 1.** Studies included in the systematic review

**eTable 2.** Studies assessed but not included in the systematic review

**eTable 3.** Risk of bias assessment

**eTable 4.** Meta-regression for age

**eTable 5.** Meta-regression for number of T2 lesions

**eFigure 1.** Risk of bias assessment summary plot

**eFigure 2.** Forest-plot of leave-one-out analysis for younger age

**eFigure 3.** Funnel plot for younger age

**eFigure 4.** Forest-plot of leave-one-out analysis for female sex and EDSS

**eFigure 5.** Funnel plot for female sex and EDSS

**eFigure 6**. Forest-plot of leave-one-out analysis for multifocal presentation

**eFigure 7.** Funnel plot for multifocal presentation

**eFigure 8**. Forest-plot of leave-one-out analysis for optic neuritis and spinal cord

**eFigure 9**. Funnel plot for optic neuritis and spinal cord

**eFigure 10.** Forest-plot of leave-one-out analysis for number of T2 lesions

**eFigure 11.** Funnel plot for number of T2 lesions

**eFigure 12.** Forest-plot of leave-one-out analysis for lesion localization

**eFigure 13.** Funnel plot for lesion localization

**eFigure 14.** Forest-plot of leave-one-out analysis for gadolinium enhancement

**eFigure 15.** Funnel plot for gadolinium enhancement

**eFigure 16.** Forest-plot of leave-one-out analysis for oligoclonal bands

**eFigure 17.** Funnel plot for oligoclonal bands

**eFigure 18.** Forest-plot of leave-one-out analysis for CSF pleocytosis

**eFigure 19.** Funnel plot for CSF pleocytosis

**eFigure 20.** Forest-plot of leave-one-out analysis for serum vitamin D deficiency and CSF NFL

**eAppendix 1. Search Strategy**

EMBASE:

'clinically isolated syndrome':ti,ab,kw AND 'multiple sclerosis':ti,ab,kw AND ('risk factor':ti,ab,kw OR 'progression':ti,ab,kw OR 'conversion':ti,ab,kw OR ‘predictors’:ti,ab,kw) NOT ('myelooptic neuropathy':ti,ab,kw OR 'radiologically isolated syndrome':ti,ab,kw) NOT (review:it OR 'case series':it OR 'case report':it)

PUBMED:

(((((((clinically isolated syndrome[Title/Abstract]) AND (multiple sclerosis[Title/Abstract])) AND ((risk factors[Title/Abstract]) OR (disease progression[Title/Abstract]) OR (conversion[Title/Abstract]) OR (predictors[Title/Abstract]))) NOT (neuromyelitis optica[MeSH Terms])) OR (radiologically isolated syndrome[MeSH Terms])) NOT (case series[Publication Type])) NOT (case report[Publication Type])) NOT (review[Publication Type])

SCOPUS:

( TITLE-ABS-KEY ( clinically AND isolated AND syndrome ) AND TITLE-ABS-KEY ( multiple AND sclerosis ) AND TITLE-ABS-KEY ( risk AND factors ) OR TITLE-ABS-KEY ( conversion ) OR TITLE-ABS-KEY ( progression ) OR TITLE-ABS-KEY ( predictors ) AND NOT TITLE-ABS-KEY ( neuromyelitis AND optica ) OR TITLE-ABS-KEY ( radiologically AND isolated AND syndrome ) ANT NOT ALL ( review ) AND NOT ALL ( case ) )

**eTable 1. Studies included in the Systematic Review**

| **Title** | **Author** | **Year of Publication** | **Country** | **N** | **Recruitment Period** | **Type of study** | **MS Diagnostic Criteria** | **Mean FU*** |
| --- | --- | --- | --- | --- | --- | --- | --- | --- |
| Conversion to multiple sclerosis after a clinically isolated syndrome of the brainstem: cranial magnetic resonance imaging, cerebrospinal fluid and neurophysiological findings. | Sastre-Garriga | 2003 | Spain | 51 | 1995-2001 | Prospective cohort | Poser | 37 |
| Do oligoclonal bands add information to MRI in first attacks of multiple sclerosis? | Tintoré | 2007 | Spain | 415 | N/A | Prospective cohort | Poser | 50 |
| Acute partial transverse myelitis: Risk factors for conversion to multiple sclerosis | Sellner | 2008 | Germany | 73 | 1999-2005 | Retrospective cohort | Mc Donald 2005 | 46 |
| MRI characteristics are predictive for CDMS in monofocal, but not in multifocal patients with a clinically isolated syndrome. | Nielsen | 2009 | Multicenter | 246 | 2002-2003 | Prospective cohort | Poser | 24 |
| Clinically isolated acute transverse myelitis: prognostic features and incidence. | Young | 2009 | New Zealand | 61 | 2001-2005 | Prospective cohort | Mc Donald 2001 | 30 |
| The chemokine CXCL13 is a prognostic marker in clinically isolated syndrome (CIS). | Brettschneider | 2010 | Germany | 91 | N/A | Prospective cohort | Mc Donald 2005 | 24 |
| Oligoclonal bands and MRI in clinically isolated syndromes: Predicting conversion time to multiple sclerosis | Ignacio | 2010 | Argentina | 40 | 1998-2006 | Prospective cohort | Poser | 60 |
| Intracortical lesions: Relevance for new MRI diagnostic criteria for multiple sclerosis | Filippi | 2010 | Italy | 119 | 2005 | Prospective cohort | Poser | 55.3 |
| Early MRI in optic neuritis: the risk for clinically definite multiple sclerosis. | Swanton | 2010 | UK | 142 | 1995-2004 | Prospective cohort | Relapse | 62 |
| Cerebrospinal fluid chitinase 3-like 1 levels are associated with conversion to multiple sclerosis. | Comabella | 2010 | Spain | 60 | >1995 | Retrospective cohort | Mc Donald 2001 | 60 |
| Prognostic value of cerebrospinal fluid analysis at the time of a first demyelinating event | Gout | 2011 | France | 208 | 1994-2006 | Prospective cohort | Poser | 42 |
| Predictive factors for multiple sclerosis in patients with clinically isolated spinal cord syndrome. | Ruet | 2011 | France | 114 | 2000-2007 | Retrospective cohort | Mc Donald 2010 | 48 |
| Clinical isolated syndrome: a 3-year follow-up study in China.Clinical isolated syndrome: a 3-year follow-up study in China. | Liu | 2011 | China | 42 | 2007-2011 | Prospective cohort | Poser | 38 |
| Assessment of outcome predictors after first attack of optic neuritis | Mamarabadi | 2011 | Iran | 109 | 2001-2009 | Retrospective cohort | Mc Donald 2001 | 98 |
| Volumetric MRI markers and predictors of disease activity in early multiple sclerosis: a longitudinal cohort study. | Kalincik | 2012 | Czech Republic | 220 | 2005-2009 | Prospective cohort | Mc Donald 2005 | 24 |
| The association of intrathecal immunoglobulin synthesis and cortical lesions predicts disease activity in clinically isolated syndrome and early relapsing-remitting multiple sclerosis. | Calabrese | 2012 | Italy | 46 | 2006-2007 | Prospective cohort | Mc Donald 2005 | 36 |
| Predictors of Conversion to Multiple Sclerosis in Patients with Clinical Isolated Syndrome Using the 2010 Revised McDonald Criteria. | Alroughani | 2012 | Kuwait | 97 | N/A | Prospective cohort | Mc Donald 2010 | 24 |
| Thalamic atrophy is associated with development of clinically definite multiple sclerosis. | Zivadinov | 2013 | Australia | 216 | 2005-2009 | Prospective cohort | Mc Donald 2005 | 24 |
| Risk of multiple sclerosis following clinically isolated syndrome: a 4-year prospective study. | D'Alessandro | 2013 | Italy | 168 | 2004-2007 | Prospective cohort | Mc Donald 2001 | 24 |
| Impact of cigarette smoking on conversion from clinically isolated syndrome to clinically definite multiple sclerosis. | Arikanoglu | 2013 | Turkey | 95 | 2007-2011 | Retrospective cohort | Relapse | 60 |
| Environmental Factors Associated with Disease Progression after the First Demyelinating Event: Results from the Multi-Center SET Study | Horakova | 2013 | Multicenter | 211 | 2005-2009 | Prospective cohort | Relapse | 24 |
| Clinical and biomarker assessment of demyelinating events suggesting multiple sclerosis. | Gajofatto | 2013 | Italy | 46 | 1996-2010 | Retrospective cohort | Poser | 60 |
| Cerebrospinal fluid oligoclonal IgM bands predict early conversion to clinically definite multiple sclerosis in patients with clinically isolated syndrome. | Ferraro | 2013 | Italy | 205 | 2007-2011 | Retrospective cohort | Mc Donald 2010 | 36 |
| Validation of semaphorin 7A and ala-Œ≤-his-dipeptidase as biomarkers associated with the conversion from clinically isolated syndrome to multiple sclerosis. | Cantó | 2014 | Spain | 56 | >1995 | Prospective cohort | Poser | 96 |
| Evaluation of visual structural and functional factors that predict the development of multiple sclerosis in clinically isolated syndrome patients. | Perez-Rico | 2014 | Spain | 29 | N/A | Prospective cohort | Mc Donald 2010 | 12 |
| Subclinical central inflammation is risk for RIS and CIS conversion to MS. | Rossi | 2015 | Italy | 39 | N/A | Prospective cohort | Mc Donald 2010 | 24 |
| Longitudinal assessment of clinically isolated syndrome with diffusion tensor imaging and volumetric MRI. | Kolasa | 2015 | Finland | 20 | 2006-2012 | Prospective cohort | Mc Donald 2005 | 48 |
| Glial and neuronal markers in cerebrospinal fluid predict progression in multiple sclerosis. | Martínez | 2015 | Spain | 109 | 1997-2011 | Prospective cohort | Mc Donald 2005 | 132 |
| Fatigue at time of CIS is an independent predictor of a subsequent diagnosis of multiple sclerosis. | Runia | 2015 | Netherlands | 127 | N/A | Prospective cohort | Poser | 24 |
| Diagnostic and Prognostic Value of the Cerebrospinal Fluid Concentration of Immunoglobulin Free Light Chains in Clinically Isolated Syndrome with Conversion to Multiple Sclerosis. | Makshakov | 2015 | Russia | 198 | 2012-2015 | Retrospective cohort | Mc Donald 2010 | 24 |
| Defining high, medium and low impact prognostic factors for developing multiple sclerosis | Tintoré | 2015 | Spain | 1015 | >1995 | Prospective cohort | Poser | 81 |
| Conversion from clinically isolated syndrome to multiple sclerosis: A large multicantre study | Kuhle | 2015 | Multicenter | 1047 | 1986-2011 | Prospective cohort | Poser | 48 |
| Chitinase 3-like 1: prognostic biomarker in clinically isolated syndromes. | Cantó | 2015 | Multicenter - Europe | 813 | N/A | Prospective cohort | Mc Donald 2005 | 60 |
| Abnormally high levels of anti-collagen type IV IgG antibodies in the serum of patients with a clinically isolated syndrome correlate with an increased risk of conversion to MS. | Sadarzanska- Terzieva | 2015 | Bulgaria | 40 | 2008 | Prospective cohort | Mc Donald 2005 | 60 |
| Protein-Based Classifier to Predict Conversion from Clinically Isolated Syndrome to Multiple Sclerosis. | Borras | 2016 | Spain | 50 | N/A | Prospective cohort | Poser | 60 |
| Neurofilament light chain level is a weak risk factor for the development of MS | Arrambide | 2016 | Spain | 68 | N/A | Prospective cohort | Poser | 78 |
| Dopaminergic receptors and adrenoceptors in circulating lymphocytes as putative biomarkers for the early onset and progression of multiple sclerosis. | Cosentino | 2016 | Italy | 21 | N/A | Prospective cohort | Mc Donald 2005 | 12 |
| Deregulation of microRNA-181c in cerebrospinal fluid of patients with clinically isolated syndrome is associated with early conversion to relapsing-remitting multiple sclerosis. | Ahlbrecht | 2016 | Germany | 58 | 2005-2011 | Prospective cohort | Mc Donald 2005 | 12 |
| Soluble CD27 Levels in Cerebrospinal Fluid as a Prognostic Biomarker in Clinically Isolated Syndrome. | van der Vuurst de Vries | 2017 | Netherlands | 77 | 2002-2015 | Prospective cohort | Mc Donald 2010 | 54 |
| Prognostic value of oligoclonal IgG bands in Japanese clinically isolated syndrome converting to clinically definite multiple sclerosis. | Kinoshita | 2017 | Japan | 26 | 2006-2014 | Prospective cohort | Poser | 60 |
| Postural Orthostatic Tachycardia Predicts Early Conversion to Multiple Sclerosis after Clinically Isolated Syndrome. | Habek | 2017 | Croatia | 62 | 2014-2016 | Prospective cohort | Mc Donald 2010 | 6 |
| Multiple biomarkers improve the prediction of multiple sclerosis in clinically isolated syndromes | Martinelli | 2017 | Italy | 243 | 2000-2013 | Retrospective cohort | Poser | 87 |
| Clinically Isolated Syndrome According to McDonald 2010: Intrathecal IgG Synthesis Still Predictive for Conversion to Multiple Sclerosis. | Schwenkenbecher | 2017 | Germany | 120 | 2010-2015 | Prospective cohort | Mc Donald 2010 | 47 |
| The Role of Assay of Free Immunoglobulin Light Chains in the Diagnosis of the Onset of Multiple Sclerosis | Nazarov | 2018 | Russia | 260 | 2012-2016 | Prospective cohort | Mc Donald 2010 | 24 |
| Smoking at time of CIS increases the risk of clinically definite multiple sclerosis. | van der Vuurst de Vries | 2018 | Netherlands | 250 | 2006-2017 | Prospective cohort | Poser | 58 |
| Prediction of Conversion from Clinically Isolated Syndrome to Multiple Sclerosis According to Baseline | Piri-Cinar | 2018 | Turkey | 41 | 2009-2010 | Prospective cohort | Mc Donald 2010 | 24 |
| MRI in predicting conversion to multiple sclerosis within 1 year. | Eran | 2018 | USA | 46 | 2014-2016 | Prospective cohort | Mc Donald 2010 | 12 |
| Intrathecal immunoglobulin M production: A promising high-risk marker in clinically isolated syndrome patients. | Huss | 2018 | Germany | 126 | 2002-2014 | Prospective cohort | Mc Donald 2010 | 34 |
| Early predictors of conversion in patients with clinically isolated syndrome: a preliminary Egyptian study | Shaheen | 2018 | Egypt | 43 | 2014-2015 | Prospective cohort | Mc Donald 2010 | 12 |
| Clinical usefulness of prognostic biomarkers in optic neuritis. | Tejada-Valverde | 2018 | Spain | 68 | 2005-2014º | Prospective cohort | Mc Donald 2010 | 36 |
| Validation of CSF free light chain in diagnosis and prognosis of multiple sclerosis and clinically isolated syndrome: prospective cohort study in Buenos Aires. | Saez | 2019 | Argentina | 36 | 2015-2017 | Prospective cohort | Mc Donald 2017 | 28 |
| MRI of acute optic neuritis (ON) at the first episode: Can we predict the visual outcome and the development of multiple sclerosis (MS)? | Cellina | 2019 | Italy | 37 | 2015-2017 | Prospective cohort | Mc Donald 2017 | 12 |
| Early imaging predictors of long-term outcomes in relapse-onset multiple sclerosis | Brownlee | 2019 | UK | 164 | 1995-2004 | Prospective cohort | Mc Donald 2010 | 181.2 |
| Conversion of clinically isolated syndrome to multiple sclerosis: a prospective multi-center study in Eastern India. | Banerjee | 2019 | India | 82 | 2009-2016 | Prospective cohort | Mc Donald 2010 | 60 |
| Cerebrospinal fluid neurofilament light chain predicts disease activity after the first demyelinating event suggestive of multiple sclerosis | Gaetani | 2019 | Italy | 32 | 2006-2016 | Prospective cohort | Mc Donald 2010 | 45.6 |
| MR Diffusion Properties of Cervical Spinal Cord as a Predictor of Progression to Multiple Sclerosis in Patients with Clinically Isolated Syndrome | Dostál | 2020 | Czech Republic | 47 | N/A | Cross-sectional | Poser | 48 |
| IgG Index Revisited: Diagnostic Utility and Prognostic Value in Multiple Sclerosis. | Zheng | 2020 | China | 105 | 2012-2019 | Retrospective cohort | Poser | 24 |
| CSF levels of HoxB3 and YKL-40 may predict conversion from clinically isolated syndrome to relapsing remitting multiple sclerosis. | Tamam | 2020 | Turkey | 33 | 2016-2018º | Prospective cohort | Mc Donald 2017 | 24 |
| Conversion of clinically isolated syndrome to multiple sclerosis: a prospective study. | Kolcava | 2020 | Czech Republic | 64 | 2015-2019 | Prospective cohort | Mc Donald 2010 | 24 |
| Clinically isolated syndrome, oligoclonal bands and multiple sclerosis | Teixeira | 2020 | Portugal | 82 | 2009-2018 | Prospective cohort | Mc Donald 2010 | 84 |
| Signs of neuroinflammation outweigh neurodegeneration as predictors for early conversion to MS | El Fayomy | 2021 | Egypt | 42 | N/A | Prospective cohort | Mc Donald 2010 | 12 |
| Integrative biochemical, proteomics and metabolomics cerebrospinal fluid biomarkers predict clinical conversion to multiple sclerosis. | Probert | 2021 | UK | 54 | 2010-2018º | Prospective cohort | Poser | 24 |
| Evaluating Magnetic Resonance Diffusion Properties Together with Brain Volumetry May Predict Progression to Multiple Sclerosis. | Sutlik | 2021 | Czech Republic | 72 | N/A | Prospective cohort | Poser | 24 |
| Cerebrospinal fluid level of phosphorylated neurofilament heavy chain is higher in converting clinically isolated syndrome and correlates with CAMP response element-binding protein concentration | Gencer | 2021 | Turkey | 23 | N/A | Prospective cohort | Mc Donald 2010 | 36 |
| Factors Related to the Progression of Clinically Isolated Syndrome to Multiple Sclerosis: A Retrospective Study in Lithuania. | Balnytė | 2022 | Lithuania | 138 | 2015-2020 | Retrospective cohort | Mc Donald 2017 | 60 |
| Analysis of factors correlated with spinal clinically isolated syndrome conversion to multiple sclerosis. | Li | 2022 | China | 61 | 2010-2020 | Retrospective cohort | Mc Donald 2017 | 54 |
| Conversion Predictors of Clinically Isolated Syndrome to Multiple Sclerosis in Mexican Patients: A Prospective Study. | Chavarria | 2023 | Mexico | 273 | 2006-2010 | Prospective cohort | Mc Donald 2010 | 120 |
| Progression predictors of clinically isolated syndrome to multiple sclerosis: A prospective study in China. | Qiu | 2024 | China | 96 | 2018-2021 | Prospective cohort | Mc Donald 2017 | 24 |
| Prognostic relevance of the C-X-C motif chemokine ligand13 and interleukin-8 in predicting the transition fromclinically isolated syndrome to multiple sclerosis | Klíčová | 2024 | Czech Republic | 51 | 2018-2022 | Prospective cohort | Mc Donald 2017 | 24 |
| Predicting the conversion from clinically isolated syndrome to multiple sclerosis: An explainable machine learning approach. | Rasouli | 2024 | Iran | 273 | 2006-2010 | Prospective cohort | Mc Donald 2010 | 120 |
| Evaluating multiple sclerosis severity loci 30 years after a clinically isolated syndrome. | Sahi | 2024 | UK | 38 | N/A | Prospective cohort | Mc Donald 2010 | 360 |
| Cerebrospinal fluid neurofilament light chain in acute optic neuritis and its predictive ability of multiple sclerosis. | Passali | 2024 | Germany | 74 | 2018-2021 | Prospective cohort | Mc Donald 2017 | 24 |

Abbreviations: FU: follow-up, N: number of patients included, N/A: not applicable or not available, MS: multiple sclerosis, UK: United Kingdom, USA: United States of America. *: Mean follow-up in months, º: approximate

**eTable 2. Studies assessed but not included in the Systematic Review**

| **Title** | **Author** | **Year**  **Publication** | **Country** | **N** | **Type of study** | **MS criteria** | **Mean FU*** |
| --- | --- | --- | --- | --- | --- | --- | --- |
| The influence of HLA-DR and -DQ alleles on progression to multiple sclerosis following a clinically isolated syndrome. | Kelly | 1993 | USA | 70 | Prospective cohort | Relapse | 60 |
| The significance of brain magnetic resonance imaging abnormalities at presentation with clinically isolated syndromes suggestive of multiple sclerosis. A 5-year follow-up study | Morrissey | 1993 | UK | 89 | Prospective cohort | Poser | 60 |
| Quantitative brain MRI lesion load predicts the course of clinically isolated syndromes suggestive of multiple sclerosis | Filippi | 1994 | Italy | 84 | Prospective cohort | Poser | 63 |
| The prognostic value of brain MRI in clinically isolated syndromes of the CNS. A 10-year follow-up. | O'Riordan | 1998 | UK | 81 | Prospective cohort | Relapse | 120 |
| Multisequence MRI in clinically isolated syndromes and the early development of MS | Brex | 1999 | UK | 50 | Prospective cohort | Relapse | 12 |
| Prognostic value of MR and magnetization transfer imaging findings in patients with clinically isolated syndromes suggestive of multiple sclerosis at presentation | Iannucci | 2000 | Italy | 27 | Prospective cohort | Poser | 33 |
| 14-3-3 protein in the CSF as prognostic marker in early multiple sclerosis. | Martínez | 2001 | Spain | 38 | Retrospective cohort | Relapse | 27 |
| Isolated demyelinating syndromes: Comparison of CSF oligoclonal bands and different MR imaging criteria to predict conversion to CDMS | Tintoré | 2001 | Spain | 112 | Prospective cohort | Poser | 31 |
| Progressive ventricular enlargement in patients with clinically isolated syndromes is associated with the early development of multiple sclerosis | Dalton | 2002 | UK | 55 | Prospective cohort | Mc Donlad 2001 | 12 |
| Long-term follow up of patients with clinically isolated syndromes, relapsing-remitting and secondary progressive multiple sclerosis. | Eriksson | 2003 | Sweden | 220 | Retrospective cohort | Poser | 60 |
| Antimyelin antibodies as a predictor of clinically definite multiple sclerosis after a first demyelinating event. | Berger | 2003 | Austria | 103 | Prospective cohort | Relapse | 12 |
| Intrathecal B-cell clonal expansion, an early sign of humoral immunity, in the cerebrospinal fluid of patients with clinically isolated syndrome suggestive of multiple sclerosis. | Qin | 2003 | USA | 48 | Prospective cohort | Relapse | 72 |
| Early development of multiple sclerosis is associated with progressive grey matter atrophy in patients presenting with clinically isolated syndromes | Dalton | 2004 | UK | 58 | Prospective cohort | Mc Donlad 2001 | 36 |
| Anti-myelin antibodies do not allow earlier diagnosis of multiple sclerosis. | Lim | 2005 | UK | 47 | Prospective cohort | Mc Donlad 2001 | 12 |
| Axonal damage markers in the cerebrospinal fluid of patients with clinically isolated syndrome improve predicting conversion to definite multiple sclerosis. | Brettschneider | 2006 | Germany | 52 | Prospective cohort | Mc Donlad 2001 | 48 |
| Antimyelin antibodies and the risk of relapse in patients with a primary demyelinating event. | Rauer | 2006 | Germany | 45 | Retrospective cohort | Relapse | 60 |
| Baseline MRI predicts future attacks and disability in clinically isolated syndromes | Tintoré | 2006 | Spain | 156 | Prospective cohort | Relapse | 84 |
| Clinically isolated syndromes: A new oligoclonal band test accurately predicts conversion to MS | Masjuan | 2006 | Spain | 52 | Prospective cohort | Poser | 72 |
| Anti-myelin antibodies in clinically isolated syndrome indicate the risk of multiple sclerosis in a Swiss cohort. | Greeve | 2007 | Switzerland | 39 | Prospective cohort | Mc Donlad 2001 | 6 |
| Intrathecal IgM-synthesis does not correlate with the risk of relapse in patients with a primary demyelinating event. | Schneider | 2007 | Germany | 42 | Retrospective cohort | Relapse | 60 |
| Anti-myelin antibodies predict the clinical outcome after a first episode suggestive of MS. | Tomassini | 2007 | Italy | 51 | Prospective cohort | Poser | 36 |
| Antimyelin antibodies in clinically isolated syndromes correlate with inflammation in MRI and CSF | Kuhle | 2007 | Switzerland | 133 | Prospective cohort | Poser | 32.9 |
| Smoking is a risk factor for early conversion to clinically definite multiple sclerosis. | Di Pauli | 2008 | Austria | 148 | Prospective cohort | Mc Donald 2001 | 36 |
| Prognostic value of high-field proton magnetic resonance spectroscopy in patients presenting with clinically isolated syndromes suggestive of multiple sclerosis. | Wattjes | 2008 | Germany | 25 | Prospective cohort | Mc Donald 2005 | 8 |
| The persistency of high levels of pSTAT3 expression in circulating CD4+ T cells from CIS patients favors the early conversion to clinically defined multiple sclerosis. | Frisullo | 2008 | Italy | 18 | Prospective cohort | Relapse | 15 |
| Abrogation of T cell quiescence characterizes patients at high risk for multiple sclerosis after the initial neurological event | Corvol | 2008 | USA | 62 | Prospective cohort | Mc Donlad 2001 | 20 |
| A three-year, multi-parametric MRI study in patients at presentation with CIS | Rocca | 2008 | Multicenter | 263 | Retrospective cohort | Poser | 37.2 |
| Acute transverse myelitis with normal brain MRI: Long-term risk of MS | Perumal | 2008 | USA | 58 | Prospective cohort | Poser | 61.8 |
| IgG antibodies against measles, rubella, and varicella zoster virus predict conversion to multiple sclerosis in clinically isolated syndrome. | Brettschneider | 2009 | Germany | 89 | Prospective cohort | Mc Donald 2005 | 24 |
| Callosal lesion predicts future attacks after clinically isolated syndrome. | Jafari | 2009 | Netherlands | 158 | Prospective cohort | Poser | 39 |
| CSF proteome analysis in clinically isolated syndrome (CIS): candidate markers for conversion to definite multiple sclerosis. | Tumani | 2009 | Germany | 16 | Prospective cohort | Relapse | 24 |
| Clinical predictors of early second event in patients with clinically isolated syndrome | Mowry | 2009 | USA | 330 | Prospective cohort | Relapse | 12 |
| Brainstem lesions in clinically isolated syndromes | Tintoré | 2010 | Spain | 246 | Prospective cohort | Poser | 92.4 |
| Predicting conversion to MS--the role of a history suggestive of demyelination. | Morrow | 2010 | USA | 116 | Retrospective cohort | Mc Donald 2005 | 120 |
| Brain atrophy and lesion load measures over 1 year relate to clinical status after 6 years in patients with clinically isolated syndromes. | Di Filippo | 2010 | UK | 99 | Prospective cohort | Poser | 72 |
| Role of cerebrospinal fluid neurofilament light chain and N-Acetyl Aspartate in predicting the evolution to multiple sclerosis of clinically isolated syndrome suggestive of multiple sclerosis | Tortorella | 2010 | Italy | 59 | Prospective cohort | Mc Donald 2010 |  |
| The risk of relapse after a clinically isolated syndrome is related to the pattern of oligoclonal bands | Boscá | 2010 | Spain | 192 | Prospective cohort | Poser | 74 |
| Clinical features of CIS of the brainstem/cerebellum of the kind seen in MS | Sastre-Garriga | 2010 | Spain | 75 | Prospective cohort | Poser | 37 |
| Elevated Epstein-Barr virus-encoded nuclear antigen-1 immune responses predict conversion to multiple sclerosis | Lunemann | 2010 | Spain | 197 | Prospective cohort | Relapse | 84 |
| Cognitive impairment predicts conversion to multiple sclerosis in clinically isolated syndromes | Zipoli | 2010 | Italy | 56 | Prospective cohort | Mc Donlad 2001 | 42 |
| Predictive value of motor evoked potentials in clinically isolated syndrome. | Pallix-Guyot | 2011 | France | 28 | Prospective cohort | Poser | 24 |
| Increased peripheral blood CD5+ B cells predict earlier conversion to MS in high-risk clinically isolated syndromes. | Villar | 2011 | Spain | 55 | Prospective cohort | Poser | 36 |
| Cerebrospinal fluid CXCL13 in multiple sclerosis: a suggestive prognostic marker for the disease course. | Khademi | 2011 | Switzerland | 837 | Retrospective cohort | Mc Donlad 2001 |  |
| Relevance of brain lesion distribution and frequency to the subsequent conversion of a clinically isolated syndrome to multiple sclerosis | Giorgio | 2011 | Italy | 657 | Retrospective cohort | Not specific | 12 |
| Spinal cord involvement: An underestimated but powerful tool in diagnosing multiple sclerosis | Sombekke | 2011 | Netherlands | 137 | Prospective cohort | Mc Donald 2010 | 66 |
| Endotoxin levels predicts conversion from CIS to MS | Kreft | 2011 | Netherlands | 58 | Prospective cohort | Not specific | 33 |
| Evoked potential abnormalities predict disability progression at 5 years in patients with CIS | Di Maggio | 2011 | Italy | 91 | Retrospective cohort | Not specific |  |
| Evaluation of soluble HLA-G as a biomarker for multiple sclerosis | Waschbisch | 2011 | Germany | 209 | Retrospective cohort | Not specific | 64 |
| The predictive value of gray matter atrophy in clinically isolated syndromes | Calabrese | 2011 | Italy | 147 | Prospective cohort | Not specific | 48 |
| Assessing the value of spinal cord lesions in predicting development of multiple sclerosis in patients with clinically isolated syndromes. | Patrucco | 2012 | Argentina | 75 | Prospective cohort | Poser | 45 |
| High levels of cerebrospinal fluid free kappa chains predict conversion to multiple sclerosis. | Villar | 2012 | Spain | 78 | Prospective cohort | Relapse | 49 |
| Predictive nature of IgM anti-Œ±-glucose serum biomarker for relapse activity and EDSS progression in CIS patients: a BENEFIT study analysis. | Freedman | 2012 | Multicenter | 258 | Retrospective cohort | Not specific | 60 |
| Brain lesion location and clinical status 20 years after a diagnosis of clinically isolated syndrome suggestive of multiple sclerosis. | Dalton | 2012 | UK | 74 | Retrospective cohort | Poser | 240 |
| Blood adipokines and their association to disability status and progression index in multiple sclerosis: A three-year follow up study | Natarajan | 2012 | Finland | 60 | Prospective cohort | Not specific | 36 |
| Prediction of early conversion to multiple sclerosis in clinically isolated syndrome | Piri Cinar | 2012 | Turkey | 42 | Prospective cohort | Not specific | 6 |
| NF-H CSF levels in patients with clinically isolated syndromes | Tintoré | 2012 | Spain | 77 | Prospective cohort | Not specific | 89 |
| Prediction of conversion from clinically isolated syndrome to multiple sclerosis according to baseline characteristics | Piri Cinar | 2012 | Turkey | 41 | Prospective cohort | Not specific | 24 |
| Spinal cord lesions in CIS patients: A pivotal role in diagnosis and prognosis | Sombekke | 2012 | Netherlands | 121 | Prospective cohort | Mc Donald 2010 | 64 |
| High levels of free kappa light chains incerebrospinal fluid predict conversion to multiple sclerosis | Espiño | 2012 | Spain | 103 | Prospective cohort | Not specific |  |
| Relevance of brain lesion distribution and frequency for short-term conversion of patients with clinically isolated syndrome to multiple sclerosis | Giorgio | 2012 | Italy | 1012 | Prospective cohort | Not specific | 12 |
| Incidence of conversion to multiple sclerosis in Thai patients with clinically isolated syndrome | Anamnart | 2012 | Thailand | 42 | Retrospective cohort | Mc Donald 2005 | 24 |
| Isoprostanes in clinically isolated syndrome and early multiple sclerosis as biomarkers of tissue damage and predictors of clinical course | Sbardella | 2012 | Italy | 39 | Case-Control | Mc Donald 2010 | 24 |
| Clinical impact of early brain atrophy in clinically isolated syndromes. | Perez-Miralles | 2013 | Italy | 176 | Prospective cohort | Mc Donald 2005 | 53 |
| Spinal cord lesions in patients with clinically isolated syndrome: a powerful tool in diagnosis and prognosis. | Sombekke | 2013 | Netherlands | 121 | Prospective cohort | Mc Donald 2010 | 64 |
| Location of brain lesions predicts conversion of clinically isolated syndromes to multiple sclerosis. | Giorgio | 2013 | Italy | 1165 | Retrospective cohort | Relapse | 12 |
| Fatigue, but not vitamin D, predicts conversion to multiple sclerosis in clinically isolated syndrome (CIS) patients | Runia | 2013 | Netherlands | 146 | Prospective cohort | Not specific |  |
| Neurofilament light subunit and evolution of clinically isolated syndrome (CIS) to multiple sclerosis | Arrambide | 2013 | Spain | 93 | Prospective cohort | Mc Donald 2005 | 24 |
| Predictor magnetic resonance imaging factors of conversion to multiple sclerosis in patients with clinical isolated syndrome | Piri Cinar | 2013 | Turkey | 130 | Retrospective cohort | Not specific |  |
| Cerebrospinal fluid CXCL13 as a prognostic marker in clinically isolated syndrome patients | Ferraro | 2013 | Italy | 110 | Prospective cohort | Mc Donald 2010 |  |
| Smoking worsens multiple sclerosis prognosis: Evidence indicating more than one pathogenic pathway | Correale | 2013 | Argentina | 95 | Case-Control | Not specific |  |
| Retinal nerve fiber layer thickness as prognostic indicator for the conversion to clinically definite multiple sclerosis in clinically isolated syndrome with acute retrobulbar optic neuritis | Giambene | 2013 | Italy | 18 | Retrospective cohort | Not specific |  |
| Clinical and demographic predictors of the risk of conversion from clinically isolated syndrome to multiple sclerosis: A population-based study | Langer-Gould | 2013 | USA | 305 | Retrospective cohort | Not specific | 36 |
| Are obesity and tobacco smoke risk factors for conversion from clinically isolated syndrome to multiple sclerosis? | Langford | 2013 | USA | 305 | Retrospective cohort | Relapse | 36 |
| Do motor impairments detected on onset of multiple sclerosis suggest an early second attack? A prospective study | Kalron | 2013 | Israel | 49 | Prospective cohort | Mc Donald 2005 | 12 |
| Isoprostanes in clinically isolated syndrome and early multiple sclerosisas biomarkers of tissue damage and predictors of clinical course | Sbardella | 2013 | Italy | 39 | Prospective cohort | Relapse | 24 |
| Antimyelin antibodies as predictors of disability after clinically isolated syndrome. | Findling | 2014 | Switzerland | 93 | Prospective cohort | Relapse | 20 |
| Phase white matter signal abnormalities in patients with clinically isolated syndrome and other neurologic disorders. | Hagemeier | 2014 | USA | 48 | Prospective cohort | Relapse | 36 |
| Diagnostic value of brain chronic black holes on T1-weighted MR images in clinically isolated syndromes. | Mitjana | 2014 | Spain | 520 | Prospective cohort | Poser | 24 |
| Independent predictors of time to relapse after CIS in high-risk patients | Spelman | 2014 | Australia | 3296 | Prospective cohort | Not specific |  |
| Importance of CSF analysis in the era of McDonald 2010 criteria: A retrospective multicenter study in patients with a clinically isolated syndrome | Tumani | 2014 | Germany | 406 | Prospective cohort | Mc Donald 2010 | 32 |
| Early MRI predictors of clinical progression over 48 months in patients with clinically isolated syndrome | Uher | 2014 | Czech Republic | 210 | Prospective cohort | Mc Donald 2010 | 48 |
| Greater spinal cord atrophy predicts disability status five years after a clinically isolated syndrome | Brownlee | 2014 | UK | 121 | Prospective cohort | Mc Donald 2010 | 67.56 |
| Fatigue at time of CIS is an independent predictor of a subsequent diagnosis of multiple sclerosis | Runia | 2014 | Netherlands | 127 | Prospective cohort | Mc Donald 2010 | 35.4 |
| Cerebrospinal fluid oligoclonal IgG bands in Japanese patients with clinically isolated syndrome | Daifu | 2014 | Japan | 34 | Prospective cohort | Not specific | 38 |
| Contribution of spinal cord MR to the diagnosis of patients with clinically isolated syndromes suggestive of multiple sclerosis | Arrambide | 2014 | Spain | 207 | Retrospective cohort | Not specific |  |
| Possible prognostic value of cerebrospinal fluid in clinically isolated syndrome | Krasulova | 2014 | Czech Republic | 124 | Prospective cohort | Not specific | 48 |
| Early predictors of multiple sclerosis after a typical clinically isolated syndrome | Ruet | 2014 | France | 652 | Prospective cohort | Mc Donald 2010 | 44.6 |
| No association of multiple sclerosis activity and progression with EBV or tobacco use in BENEFIT. | Munger | 2015 | Multicenter | 451 | Prospective cohort | Mc Donald 2001 | 6 |
| Increased PK11195-PET binding in normal-appearing white matter in clinically isolated syndrome. | Giannetti P | 2015 | UK | 27 | Case-Control | Mc Donald 2010 | 24 |
| Free kappa light chains in cerebrospinal fluid as a biomarker to assess risk conversion to multiple sclerosis. | Menendez - Valladares | 2015 | Spain | 176 | Case-Control | Mc Donald 2010 | 24 |
| Flow cytometric analysis of T cell/monocyte ratio in clinically isolated syndrome identifies patients at risk of rapid disease progression. | Nemecek | 2015 | Germany | 187 | Prospective cohort | Mc Donald 2010 | 22 |
| Early magnetic resonance imaging predictors of clinical progression after 48 months in clinically isolated syndrome patients treated with intramuscular interferon Œ≤-1a. | Uher | 2015 | Czech Republic | 210 | Prospective cohort | Mc Donlad 2001 | 48 |
| Cerebrospinal fluid CXCL13 in clinically isolated syndrome patients: Association with oligoclonal IgM bands and prediction of Multiple Sclerosis diagnosis. | Ferraro | 2015 | Italy | 110 | Prospective cohort | Mc Donald 2010 | 24 |
| Chitinase 3-like proteins as diagnostic and prognostic biomarkers of multiple sclerosis. | Hinsinger | 2015 | France | 40 | Prospective cohort | Mc Donald 2005 | 60 |
| Corpus callosum lesions in patients with a clinical isolated syndrome | Cerdá-Fuertes | 2015 | Spain | 77 | Prospective cohort | Mc Donald 2010 | 36 |
| Cerebrospinal fluid/serum CXCL13 ratio: An early prognostic marker of conversion to clinically definite multiple sclerosis | Dujmovic | 2015 | Multicenter | 55 | Prospective cohort | Not specific | 48 |
| Evaluation of postulant predictive and prognostic biomarkers in multiple sclerosis: Fetuin-A, S100B and GFAP | Altintas | 2015 | Multicenter | 23 | Prospective cohort | Not specific | 24 |
| Intrathecal synthesis of immunoglobulin free light chains in clinically isolated syndrome predicts conversion to multiple sclerosis and disability level in two years follow-up | Makshakov | 2015 | Russia | 100 | Retrospective cohort | Not specific | 24 |
| Cervical cord atrophy in clinically isolated syndrome and early multiple sclerosis: A novel paraclinical risk marker for conversion to manifest multiple sclerosis? | Hagstrom | 2015 | Germany | 45 | Prospective cohort | Not specific | 24 |
| Cerebrospinal fluid free kappa chains as a possible biomarker to assay risk conversion to multiple sclerosis | Menendez | 2015 | Spain | 176 | Case-Control | Mc Donald 2010 | 24 |
| Importance of cerebrospinal fluid analysis in the era of McDonald 2010 criteria: a German-Austrian retrospective multicenter study in patients with a clinically isolated syndrome. | Huss | 2016 | Germany | 362 | Retrospective cohort | Mc Donald 2010 | 48 |
| Quantifying risk of early relapse in patients with first demyelinating events: Prediction in clinical practice. | Spelman | 2016 | Multicenter | 3296 | Prospective cohort | Poser | 24 |
| Midsagittal corpus callosum area and conversion to multiple sclerosis after clinically isolated syndrome: A multicentre Australian cohort study. | Odenthal | 2016 | Australia | 143 | Case-Control | Mc Donald 2005 | 60 |
| Risk factors for convertion to clinically defined multiple sclerosis after clinically isolated syndrome in a racially mixed Brazilian cohort. | Wing | 2016 | Brazil | 122 | Retrospective cohort | Relapse | 12 |
| Increased expression of Tbet in CD4(+) T cells from clinically isolated syndrome patients at high risk of conversion to clinically definite MS. | Basedo | 2016 | Ireland | 20 | Prospective cohort | Relapse | 12 |
| Contribution of the symptomatic lesion in establishing MS diagnosis and prognosis. | Tintore | 2016 | Spain | 1107 | Prospective cohort | Mc Donald 2010 | 12 |
| Soluble CD27 levels in CSF as a prognostic biomarker in clinically isolated syndrome | Van Der Vuurst | 2016 | Netherlands | 77 | Prospective cohort | Not specific | 52 |
| IL-6/IL-10 producing B cells in patients with clinically isolated syndrome: Association with radiological/clinical evolution | Guerrier | 2016 | France | 46 | Prospective cohort | Not specific | 12 |
| Prognostic role of cerebrospinal fluid neurofilament, chitinase-3-like and tau levels in patients with clinically isolated syndrome | Ferraro | 2016 | Italy | 44 | Prospective cohort | Not specific |  |
| Vitamin D metabolic pathway alterations and risk of multiple sclerosis in patients with clinically isolated syndromes | Farina | 2016 | Italy | 114 | Retrospective cohort | Not specific | 92.5 |
| Myeloid microvesicles and risk of multiple sclerosis in patients with clinically isolated syndromes | Dalla-Costa | 2016 | Italy | 101 | Prospective cohort | Mc Donald 2010 | 36 |
| Postural orthostatic tachycardia predicts clinical and MRI disease activity within the six months of a clinically isolated syndrome | Habek | 2016 | Croatia | 58 | Prospective cohort | Relapse | 6 |
| Anti-HHV6 IgG levels are associated with hazard of conversion to clinically definite multiple sclerosis after CIS | Tao | 2016 | Australia | 279 | Prospective cohort | Mc Donald 2010 | 60 |
| Immunological markers as risk factors in clinically isolated syndrome (MS) | Posova | 2016 | Czech Republic | 212 | Prospective cohort | Not specific | 48 |
| Quality of life does not predict conversion from CIS to definite MS: A five-year prospective study | Baldin | 2016 | Italy | 162 | Prospective cohort | Not specific | 60 |
| Vitamine D metabolic pathway abnormalities and risk of multiple sclerosis in patients with clinically isolated syndromes | Martinelli | 2016 | Multicenter | 120 | Prospective cohort | Not specific | 84 |
| Clinically isolated syndrome (CIS): Measure of axonal status | Fernnandez | 2016 | Spain | 20 | Prospective cohort | Not specific |  |
| Spinal cord lesions: A modest contributor to  diagnosis in clinically isolated syndromes but a  relevant prognostic factor | Arrambide | 2017 | Spain | 207 | Prospective cohort | Mc Donald 2010 | 36 |
| High risk of early conversion to multiple sclerosis in clinically isolated syndromes with dissemination in space at baseline. | Gaetani | 2017 | Italy | 137 | Retrospective cohort | Mc Donald 2010 | 36 |
| Sodium intake and multiple sclerosis activity and progression in BENEFIT. | Fitzgerald | 2017 | USA | 465 | Prospective cohort | Mc Donald 2010 | 60 |
| Relevance of early cervical cord volume loss in the disease evolution of clinically isolated syndrome and early multiple sclerosis: a 2-year follow-up study. | Hagstrom | 2017 | Germany | 53 | Prospective cohort | Mc Donald 2005 | 24 |
| Clinical presentation of clinically isolated syndrome (CIS); A long term follow up study in Iranian patients | Izadi | 2017 | Iran | 143 | Prospective cohort | Not specific | 36 |
| Evaluation of the predictive value of three serum and CSF biomarkers for the development of clinically definite multiple sclerosis (CDMS) following an initial clinically isolated demyelinating event (CIS) | Yaghmour | 2017 | Israel | 29 | Prospective cohort | Not specific | 60 |
| Predicting conversion from clinically isolated syndrome to multiple sclerosis: Identification of a cerebrospinal fluid neurofilament light cut-off value | Gaetani | 2017 | Sweden | 32 | Prospective cohort | Mc Donald 2010 | 36 |
| Vitamin D deficiency predicts early conversion of clinically isolated syndrome to clinically definite multiple sclerosis: A preliminary Egyptian study | Shaheen | 2017 | Egypt | 43 | Prospective cohort | Mc Donald 2010 | 12 |
| Health-related quality of life in clinically isolated syndrome and risk of conversion to multiple sclerosis. | Baldin | 2018 | Italy | 162 | Prospective cohort | Mc Donald 2001 | 60 |
| Does vitamin D deficiency predict early conversion of clinically isolated syndrome? A preliminary Egyptian study. | Shaheen | 2018 | Egypt | 43 | Case-Control | Mc Donald 2010 | 12 |
| Multi-component relaxation in clinically isolated syndrome: Lesion myelination may predict multiple sclerosis conversion. | Kitzler | 2018 | Germany | 16 | Case-Control | Mc Donald 2010 | 9 |
| Higher Serum Immunoglobulin G3 Levels May Predict the Development of Multiple Sclerosis in Individuals With Clinically Isolated Syndrome. | Trennd | 2018 | Japan | 20 | Prospective cohort | Mc Donald 2010 | 12 |
| Peripheral blood memory B cell frequency predicts conversion from clinically isolated syndrome to multiple sclerosis. | Aktura | 2018 | Turkey | 56 | Case-Control | Mc Donald 2010 | 0 |
| Non-lesional cerebellar damage in patients with clinically isolated syndrome: DTI measures predict early conversion into clinically definite multiple sclerosis. | Kugler | 2018 | Germany | 46 | Prospective cohort | Not specific | 48 |
| Association of Retinal Ganglion Cell Layer Thickness With Future Disease Activity in Patients With Clinically Isolated Syndrome. | Zimmermann | 2018 | Germany | 97 | Prospective cohort | Mc Donald 2010 | 24 |
| CSF kappa light chain ratios potential prognostic impact in clinically isolated syndrome conversion to multiple sclerosis | Salavisa | 2018 | Portugal | 31 | Retrospective cohort | Not specific | 64 |
| Diagnostic value of enhancing lesions on post-contrast T1-weighted magnetic resonance images in patients with a clinically isolated syndrome | Diaz-Sanchez | 2018 | Spain | 143 | Prospective cohort | Mc Donald 2017 | 56 |
| Clinically isolated syndrome: Natural course and diagnostic criteria | Seabra | 2018 | Portugal | 69 | Prospective cohort | Mc Donald 2010 | 24 |
| Prognostic potential of CSF biomarkers in clinically isolated syndrome | Emersic | 2018 | Slovenia | 147 | Retrospective cohort | Relapse | 48 |
| Retinal ganglion cell layer and disease activity in clinically isolated syndrome | Zimmermann | 2018 | Germany | 97 | Prospective cohort | Mc Donald 2010 | 60 |
| Prognostic value of serum neurofilaments in patients with clinically isolated syndromes | Dalla Costa | 2019 | Italy | 222 | Retrospective cohort | Mc Donald 2017 | 100 |
| A prospective, observational study on conversion of clinically isolated syndrome to multiple sclerosis during 4-year period (MS NEO study) in Taiwan. | Ro | 2019 | China | 152 | Prospective cohort | Mc Donald 2005 | 48 |
| Intrathecal IgM production is a strong risk factor for early conversion to multiple sclerosis. | Pfuhl | 2019 | Germany | 93 | Prospective cohort | Mc Donald 2010 | 24 |
| Visual evoked potentials improve the prediction of multiple sclerosis in patients with clinically isolated syndromes | Dalla Costa | 2019 | Italy | 243 | Prospective cohort | Not specific | 84 |
| Early imaging predictors of longer term multiple sclerosis risk and severity in acute optic neuritis | Gajamange | 2019 | Australia | 36 | Prospective cohort | Mc Donald 2010 | 102 |
| Menarche, pregnancies, and breastfeeding do not modify long-term prognosis in multiple sclerosis | Zuluaga, Tintoré | 2019 | Spain | 501 | Cross-sectional | Mc Donald 2010 |  |
| Slowing of brain atrophy with teriflunomide and delayed conversion to clinically definite MS. | Zivadinov | 2020 | USA | 485 | Prospective cohort | Mc Donald 2005 | 60 |
| A 30-Year Clinical and Magnetic Resonance Imaging Observational Study ofMultiple Sclerosis and Clinically Isolated Syndromes | Chung | 2020 | UK | 91 | Prospective cohort | Mc Donald 2010 | 360 |
| Nonlesional diffusely abnormal appearing white matter in clinically isolated syndrome: Prevalence, association with clinical and MRI features, and risk for conversion to multiple sclerosis. | Holmes | 2021 | Canada | 142 | Prospective cohort | Mc Donald 2005 | 46 |
| CSF extracellular vesicles and risk of disease activity after a first demyelinating event | Dalla Costa | 2021 | Italy | 212 | Prospective cohort | Relapse | 20 |
| Body mass index as a predictor of MS activity and progression among participants in BENEFIT. | Escobar | 2022 | Spain | 464 | Retrospective cohort | Mc Donald 2005 | 60 |
| T1/T2-weighted ratio in multiple sclerosis: A longitudinal study with clinical associations. | Boaventura | 2022 | Canada | 117 | Prospective cohort | Mc Donald 2017 | 12 |
| Multimodal Evoked Potentials as Potential Biomarkers of Disease Activity in Patients With Clinically Isolated Syndrome. | Dziadkowiak | 2022 | Germany | 29 | Prospective cohort | Mc Donald 2017 | 36 |
| Predictive factors of conversion of clinically isolated syndrome to clinically definite multiple sclerosis | Kolcava | 2022 | Czech Republic | 169 | Prospective cohort | Relapse | 60 |
| A Prospective Study of Serum Levels of Polyunsaturated Fatty Acids and Effects on Multiple Sclerosis Disease Activity and Progression (S40.006) | Munger | 2022 | USA | 468 | Prospective cohort | Mc Donald 2005 | 60 |
| Multimodal Evoked Potentials as Potential Biomarkers of Disease Activity in Patients With Clinically Isolated Syndrome | Dziadkowiak, | 2022 | Poland | 79 | Prospective cohort | Mc Donald 2017 | 36 |
| IPAD-based processing speed test CogEval as a potential predictor of multiple sclerosis in patients with clinically isolated syndrome | Rodriguez | 2022 | Spain | 219 | Prospective cohort | Not specific | 60 |
| Identifying genetic and epigenetic signatures for predicting conversion to clinically definite multiple sclerosis in the AusLong study cohort | Xavier | 2022 | Australia | 241 | Prospective cohort | Not specific | 120 |
| Heat shock proteins HSP 70 and HSP 90 as predictors for conversion from CIS to CDMS | Rejdak | 2023 | Poland | 54 | Prospective cohort | Not specific | 36 |
| Prognostic value of cerebrospinal fluid biomarkers in multiple sclerosis: The key role of kappa free light chains and a multivariate predictor for disease progression. | Miklušová | 2024 | Netherlands | 82 | Retrospective cohort | Not specific | 24 |

Abbreviations: FU: follow-up, N: number of patients included, MS: multiple sclerosis, UK: United Kingdom, USA: United States of America. *: Mean follow-up in months º: approximate

**eTable 3. Risk of Bias Assessment**

| **Author** | **Year** | **Selection** | **Comparability** | **Outcome** | **Overall** | **Quality** |
| --- | --- | --- | --- | --- | --- | --- |
| Kolcava | 2020 | **** | ** | ** | 8 | Good |
| Eran | 2018 | **** |  | ** | 5 | Poor |
| Banerjee | 2019 | **** | ** | ** | 8 | Good |
| Probert | 2021 | **** |  | *** | 7 | Poor |
| Dostál | 2020 | **** |  | ** | 6 | Poor |
| Tamam | 2020 | **** |  | ** | 6 | Poor |
| Tejeda-Velarde | 2018 | **** |  | ** | 6 | Poor |
| D'Alessandro | 2013 | **** | ** | ** | 8 | Good |
| Piri-Cinar | 2018 | ** |  | ** | 4 | Good |
| Borras | 2016 | *** |  | ** | 5 | Poor |
| Martínez | 2015 | **** | ** | *** | 9 | Good |
| van der Vuurst de Vries | 2018 | *** | ** | *** | 8 | Good |
| Li | 2022 | **** | ** | *** | 9 | Good |
| Rossi | 2015 | **** | ** | ** | 8 | Good |
| Gajofatto | 2013 | **** | * | ** | 7 | Good |
| Kolasa | 2015 | **** | * | ** | 7 | Good |
| Swanton | 2010 | **** | ** | *** | 9 | Good |
| Saez | 2019 | **** |  | ** | 6 | Poor |
| Ruet | 2011 | **** | ** | ** | 8 | Good |
| Schwenkenbecher | 2017 | **** |  | ** | 6 | Poor |
| Cosentino | 2016 | **** |  | ** | 6 | Poor |
| Ahlbrecht | 2016 | **** |  | ** | 6 | Poor |
| Alroughani | 2012 | **** | ** | ** | 8 | Good |
| Runia | 2015 | **** | ** | ** | 8 | Good |
| Cellina | 2019 | **** |  | ** | 6 | Poor |
| Young | 2009 | **** | ** | ** | 8 | Good |
| Brettschneider | 2010 | **** |  | ** | 6 | Poor |
| Kalincik | 2012 | **** | ** | ** | 8 | Good |
| Sastre-Garriga | 2003 | **** |  | ** | 6 | Poor |
| Arikanoglu | 2013 | **** | ** | ** | 8 | Good |
| Liu | 2011 | **** |  | ** | 6 | Poor |
| Perez-Rico | 2014 | *** | * | ** | 7 | Good |
| Huss | 2018 | **** |  | ** | 6 | Poor |
| Brettschneider | 2009 | **** |  | ** | 6 | Poor |
| Stulik | 2021 | **** |  | ** | 6 | Poor |
| Comabella | 2010 | **** | ** | ** | 8 | Good |
| Kinoshita | 2017 | **** |  | *** | 7 | Poor |
| Huss | 2016 | **** |  | ** | 6 | Poor |
| Ferraro | 2013 | **** |  | *** | 7 | Poor |
| Cantó | 2015 | **** | ** | *** | 9 | Good |
| Sadarzanska-Terzieva | 2015 | **** |  | *** | 7 | Poor |
| Perez-Miralles | 2013 | **** | ** | ** | 8 | Good |
| Eriksson | 2003 | **** |  | *** | 7 | Poor |
| Calabrese | 2012 | **** | ** | ** | 8 | Good |
| Zheng | 2020 | **** | ** | ** | 8 | Good |
| Gaetani | 2019 | ** |  | ** | 4 | Poor |
| Zivadinov | 2013 | **** | ** | ** | 8 | Good |
| Cantó | 2014 | **** | * | *** | 8 | Good |
| van der Vuurst de Vries | 2017 | **** | ** | ** | 8 | Good |
| Nielsen | 2009 | **** | ** | ** | 8 | Good |
| El Fayomy | 2021 | **** | * | ** | 7 | Good |
| Gencer | 2021 | **** |  | ** | 6 | Poor |
| Teixeira | 2020 | *** |  | ** | 5 | Poor |
| Shaheen | 2018 | **** | ** | ** | 8 | Good |
| Nazarov | 2018 | **** |  | ** | 6 | Poor |
| Habek | 2017 | **** | * | ** | 7 | Unclear |
| Kuhle | 2015 | **** | ** | ** | 8 | Good |
| Gout | 2011 | **** | ** | ** | 8 | Good |
| Sellner | 2008 | **** | ** | ** | 8 | Good |
| Brownlee | 2019 | **** | ** | *** | 9 | Good |
| Martinelli | 2017 | *** | * | ** | 7 | Unclear |
| Arrambide | 2016 | **** | ** | *** | 9 | Good |
| Tintoré | 2015 | **** | ** | *** | 9 | Good |
| Horakova | 2013 | **** |  | ** | 6 | Poor |
| Mamarabadi | 2011 | **** | ** | ** | 8 | Good |
| Filippi | 2010 | **** | ** | ** | 8 | Good |
| Ignacio | 2010 | **** |  | *** | 7 | Poor |
| Tintoré | 2007 | **** | ** | *** | 9 | Good |
| Balnytė | 2022 | **** | ** | ** | 8 | Good |
| Passali | 2024 | **** | ** | ** | 8 | Good |
| Rasouli | 2024 | **** |  | ** | 6 | Poor |
| Qiu | 2024 | **** | ** | ** | 8 | Good |
| Sahi | 2024 | **** | * | *** | 8 | Good |
| Chavarria | 2023 | **** | * | *** | 8 | Good |
| Klíčová | 2024 | **** |  | ** | 6 | Poor |
| Makshakov | 2015 | **** | * | *** | 8 | Good |

**eTable 4. Meta regression for younger age**

| **Moderator** | **Estimate (β)** | **SE** | **z value** | **p value** | **95% CI** |
| --- | --- | --- | --- | --- | --- |
| Intercept | 0.59 | 0.26 | 2.29 | 0.022 | 0.09 to 1.09 |
| Follow-up (months) | −0.003 | 0.004 | −0.84 | 0.401 | −0.01 to 0.004 |
| Study design (retrospective vs prospective cohort) | 0.08 | 0.43 | 0.20 | 0.841 | −0.73 to 0.90 |
| MS criteria: McDonald 2010 | 0.13 | 0.33 | 0.40 | 0.689 | −0.51 to 0.78 |
| MS criteria: McDonald 2017 | 0.08 | 0.49 | 0.17 | 0.868 | −0.88 to 1.04 |
| MS criteria: Poser | −0.50 | 0.33 | −1.51 | 0.130 | −1.14 to 0.15 |
| MS criteria: Clinical relapse | −0.61 | 0.70 | −0.88 | 0.380 | −1.99 to 0.76 |
| MS criteria: McDonald 2001 | 0.07 | 0.54 | 0.12 | 0.903 | −1.00 to 1.13 |
| Test of moderators: QM(df = 8) = 10.63, p = 0.22. Residual heterogeneity: I² = 75.7%. | | | | | |

**eTable 5. Meta regression for number of T2 lesions**

| **Moderator** | **Estimate (β)** | **SE** | **z value** | **p value** | **95% CI** |
| --- | --- | --- | --- | --- | --- |
| Intercept | 2.79 | 0.98 | 2.84 | 0.004 | 0.86 to 4.71 |
| MS criteria: McDonald 2017 | −2.16 | 1.92 | −1.12 | 0.261 | −5.91 to 1.60 |
| MS criteria: Poser | −2.15 | 2.52 | −0.86 | 0.392 | −7.09 to 2.78 |
| Study design (retrospective vs prospective cohort) | 1.22 | 3.46 | 0.35 | 0.726 | −5.57 to 8.00 |
| Test of moderators: QM(df = 3) = 1.68, p = 0.64. Residual heterogeneity: I² = 96.0%. | | | | | |

**eFigure 1. Risk of Bias Assessment Summary Plot**


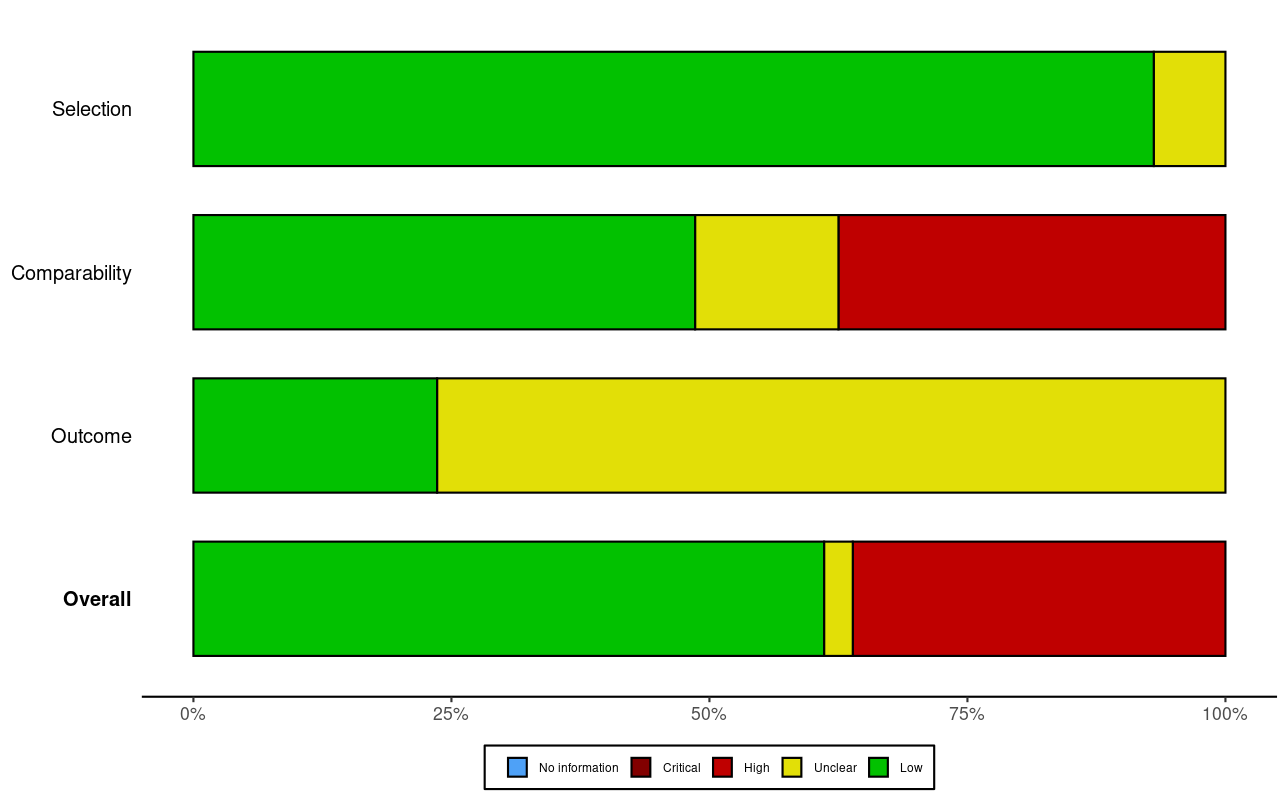


**eFigure 2. Forest-plot of leave-one-out analysis for younger age**


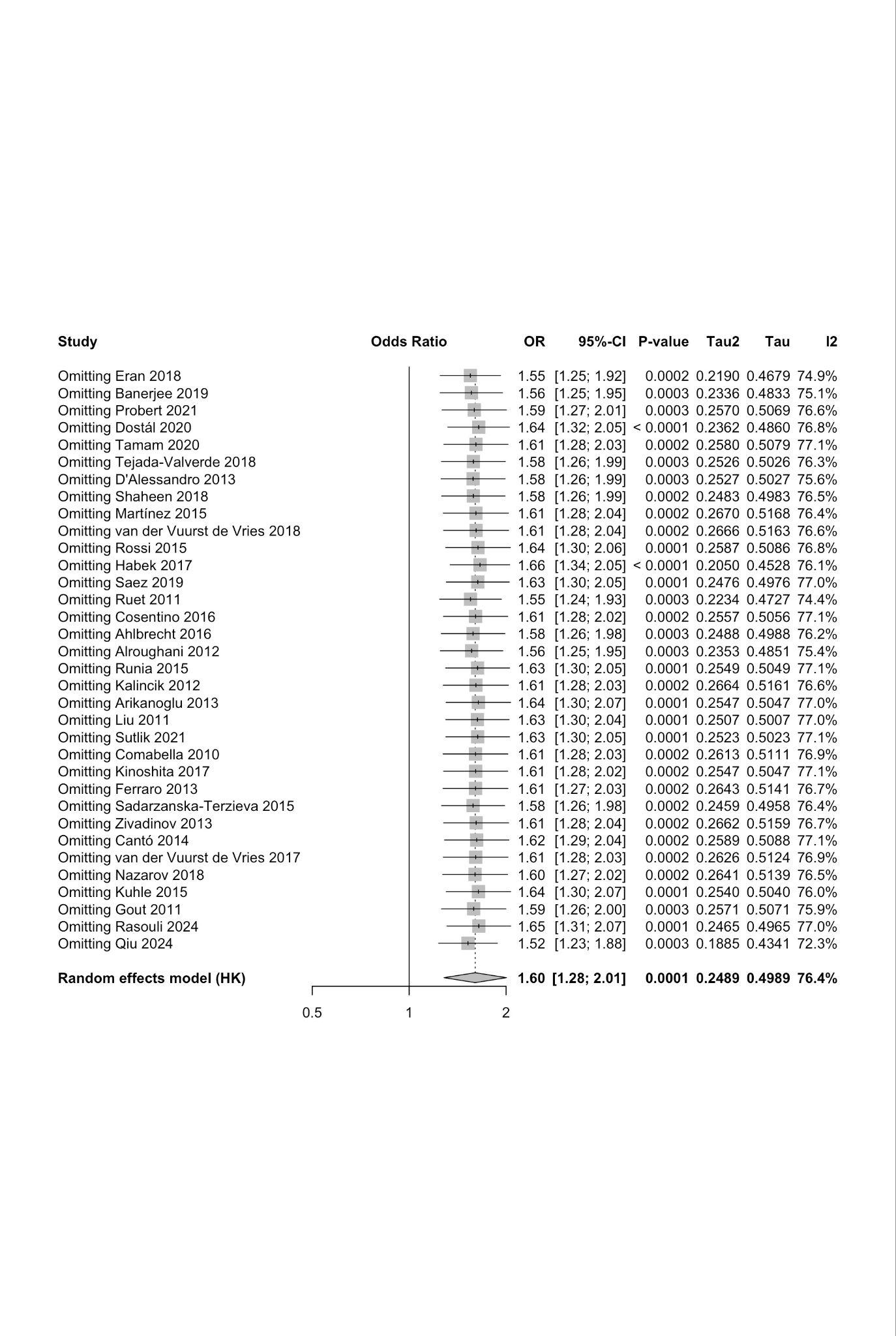


**eFigure 3. Funnel plot for younger age**

**
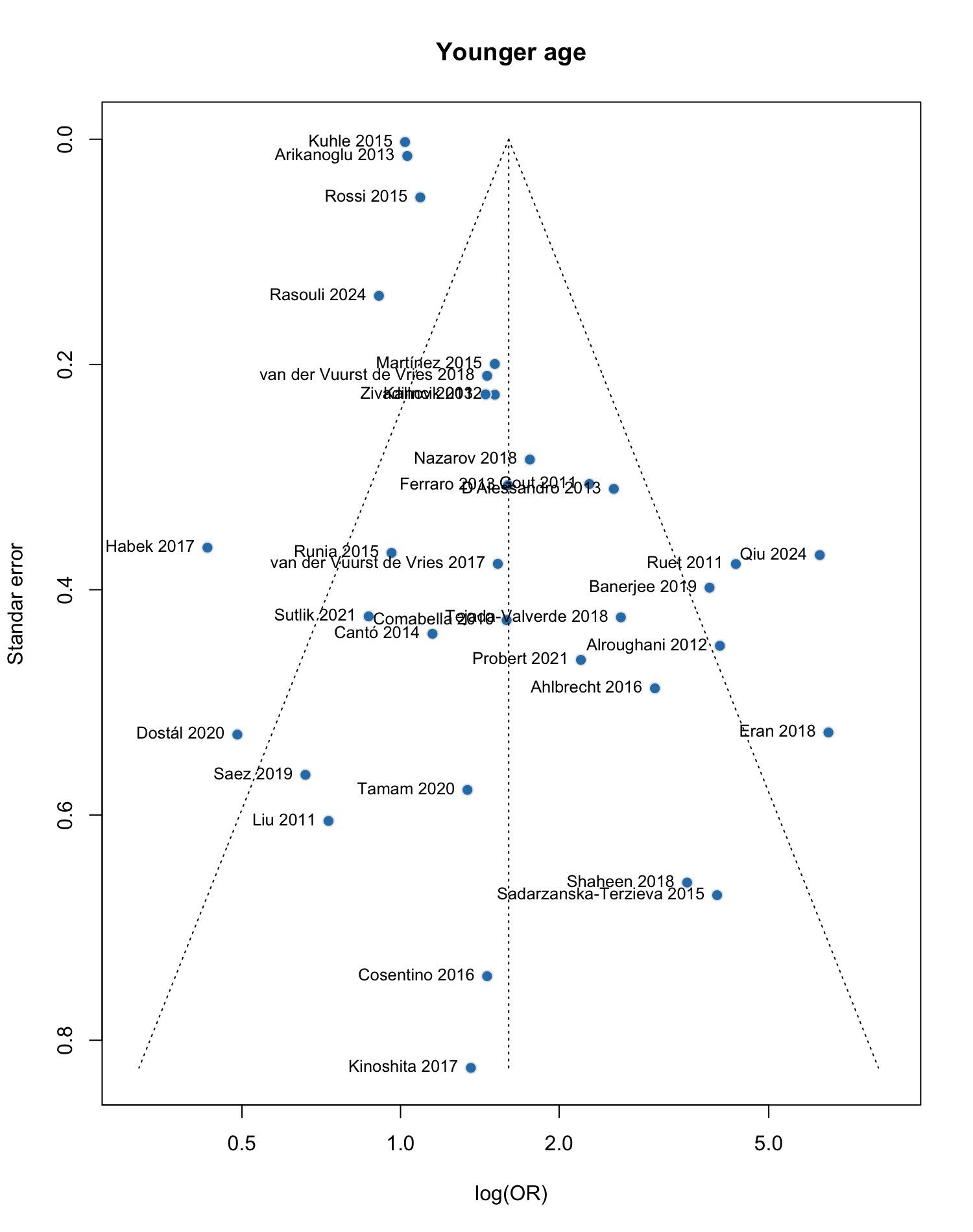
**

**eFigure 4. Forest-plot of leave-one-out analysis for female sex and EDSS**

1. **Female sex**

**
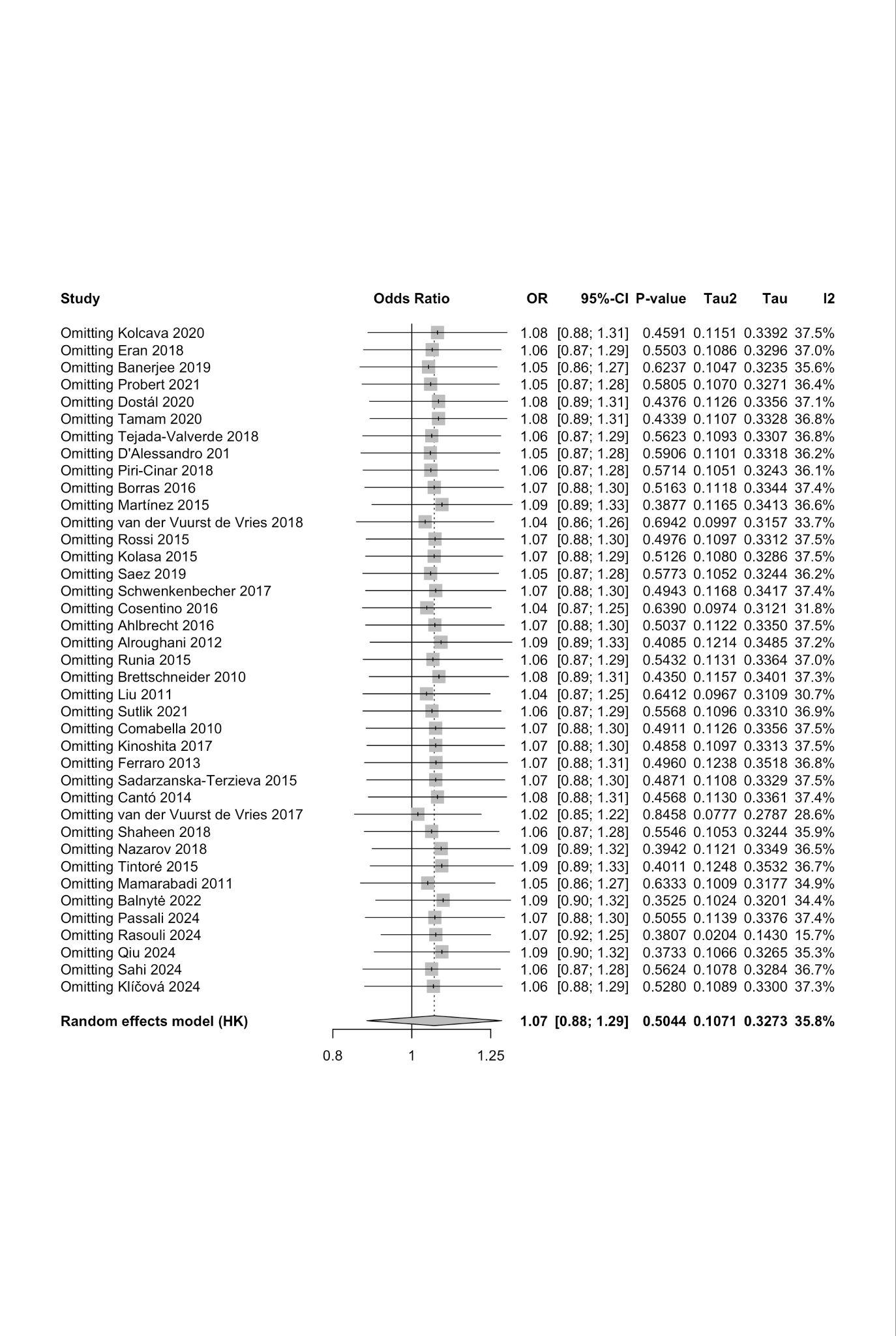
**

1. **EDSS**

**
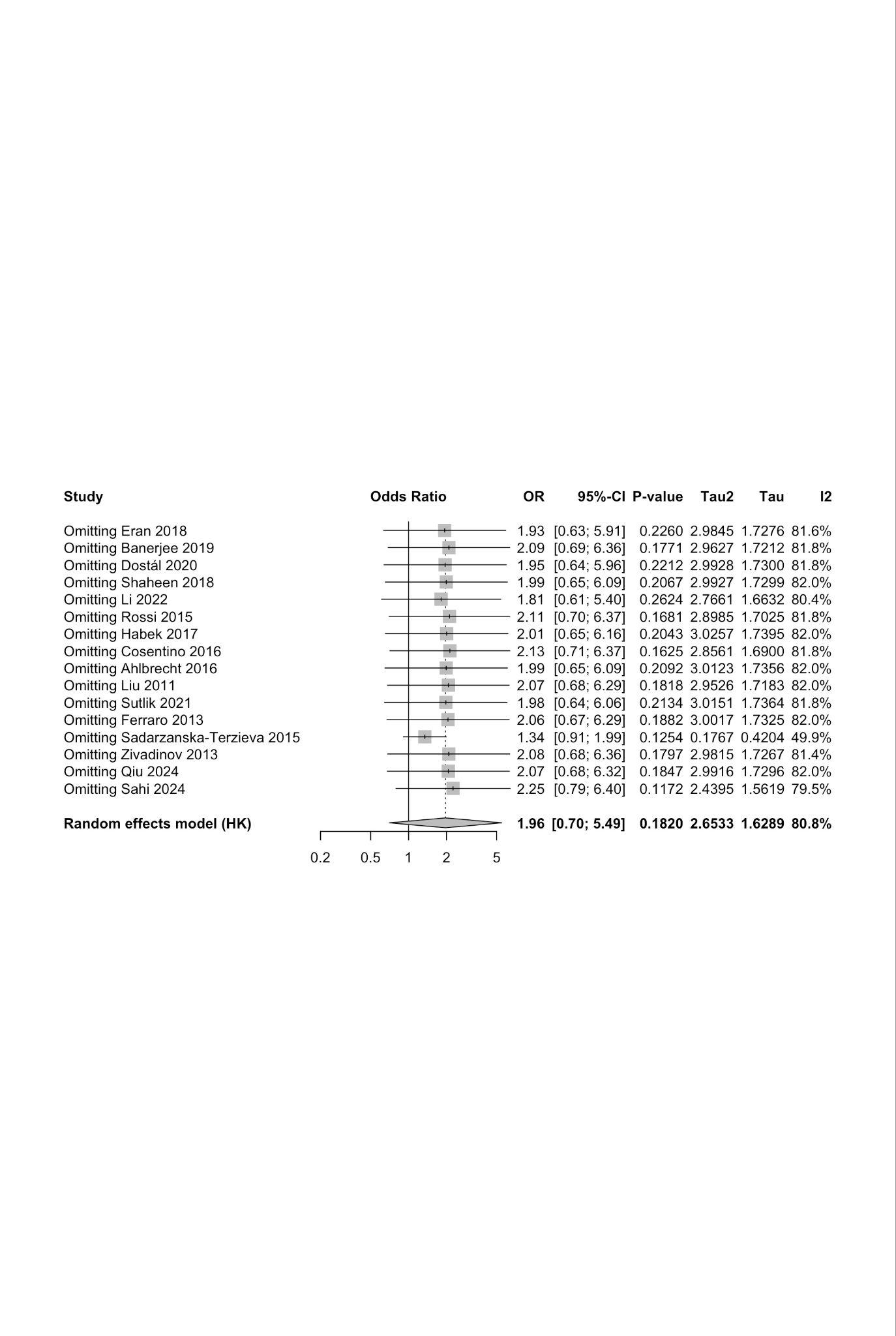
**

**eFigure 5. Funnel plot for female sex and EDSS**

1. **Female sex**

**
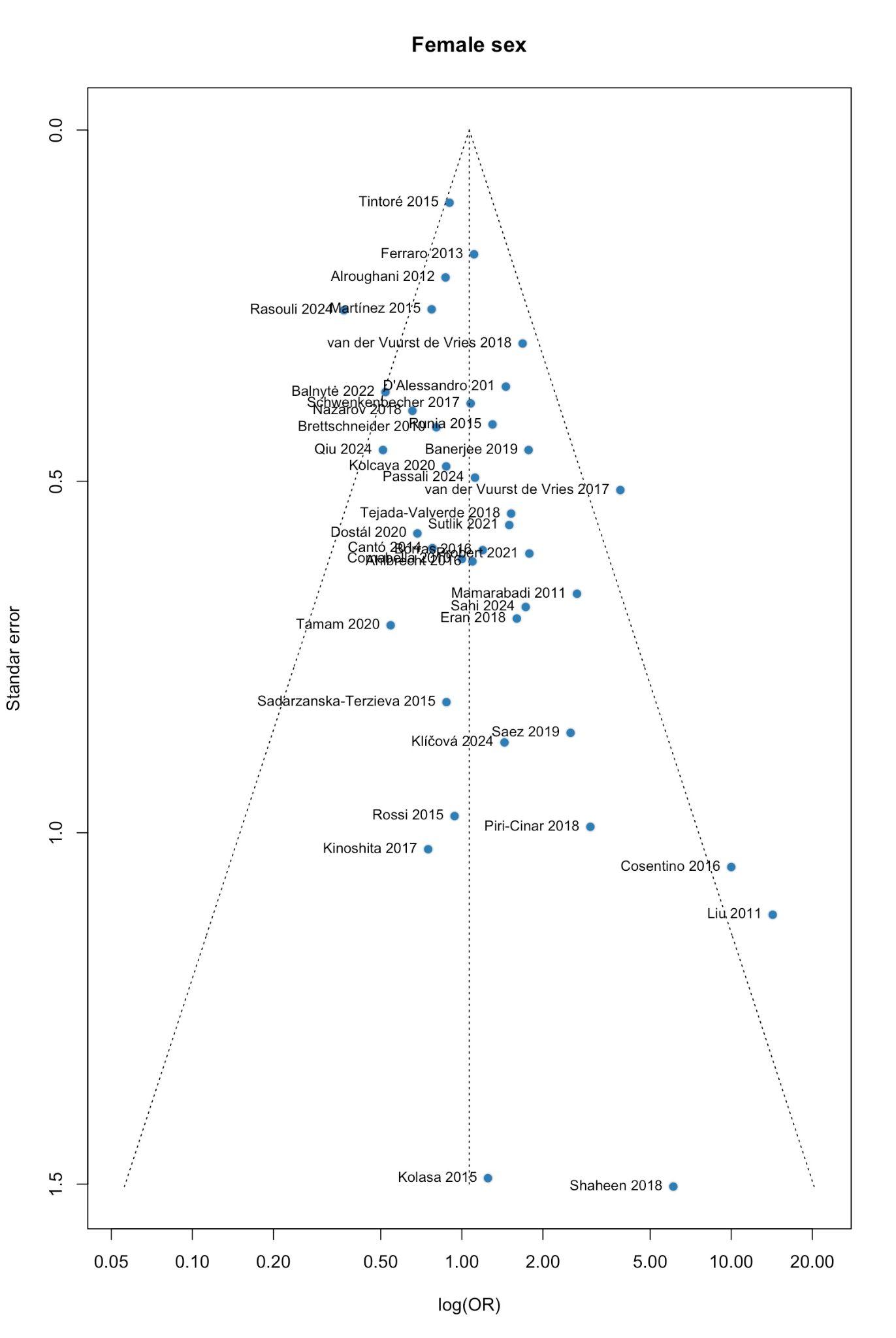
**

1. **EDSS**

**
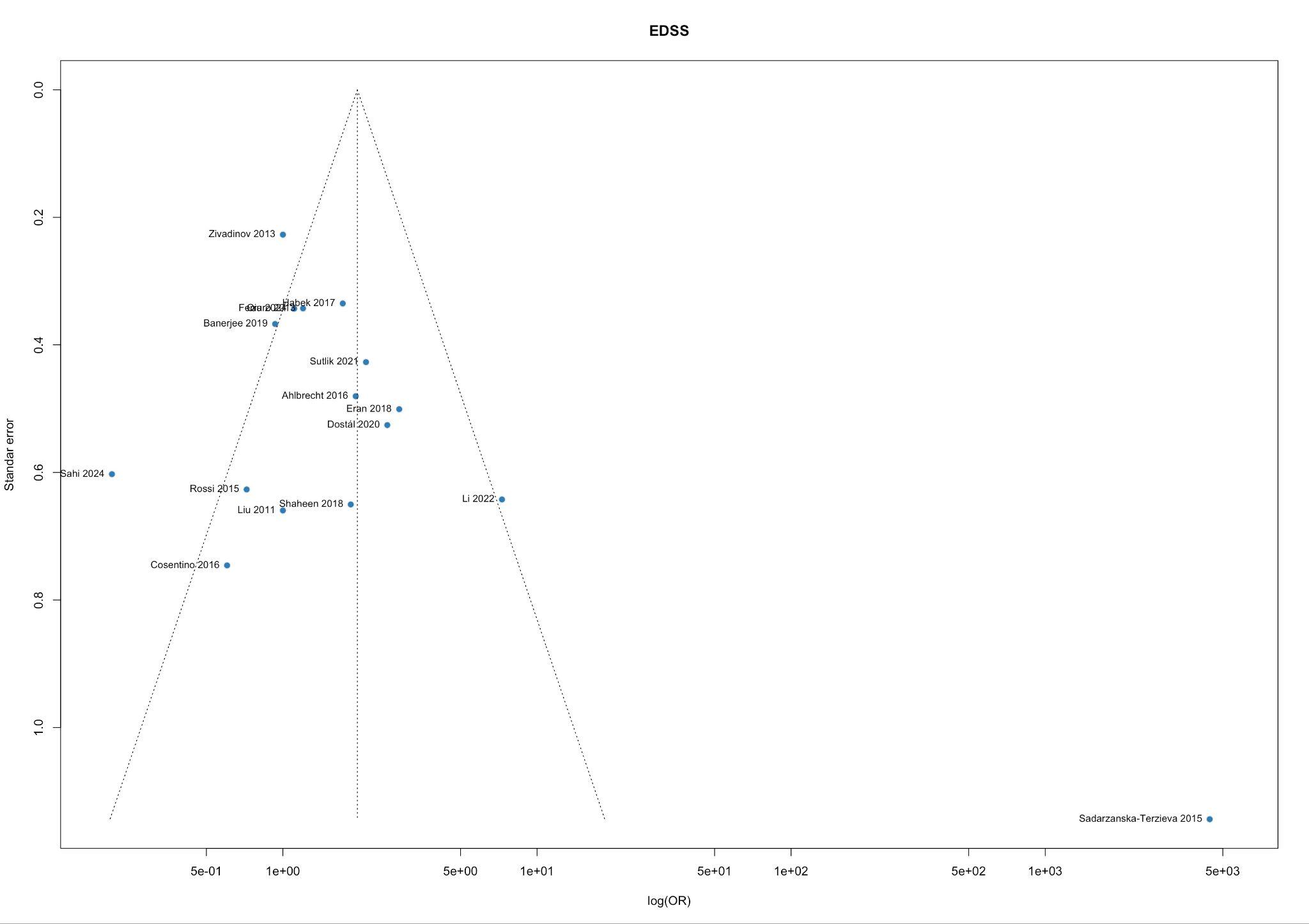
**

**eFigure 6**. **Forest-plot of leave-one-out analysis for multifocal presentation**

**
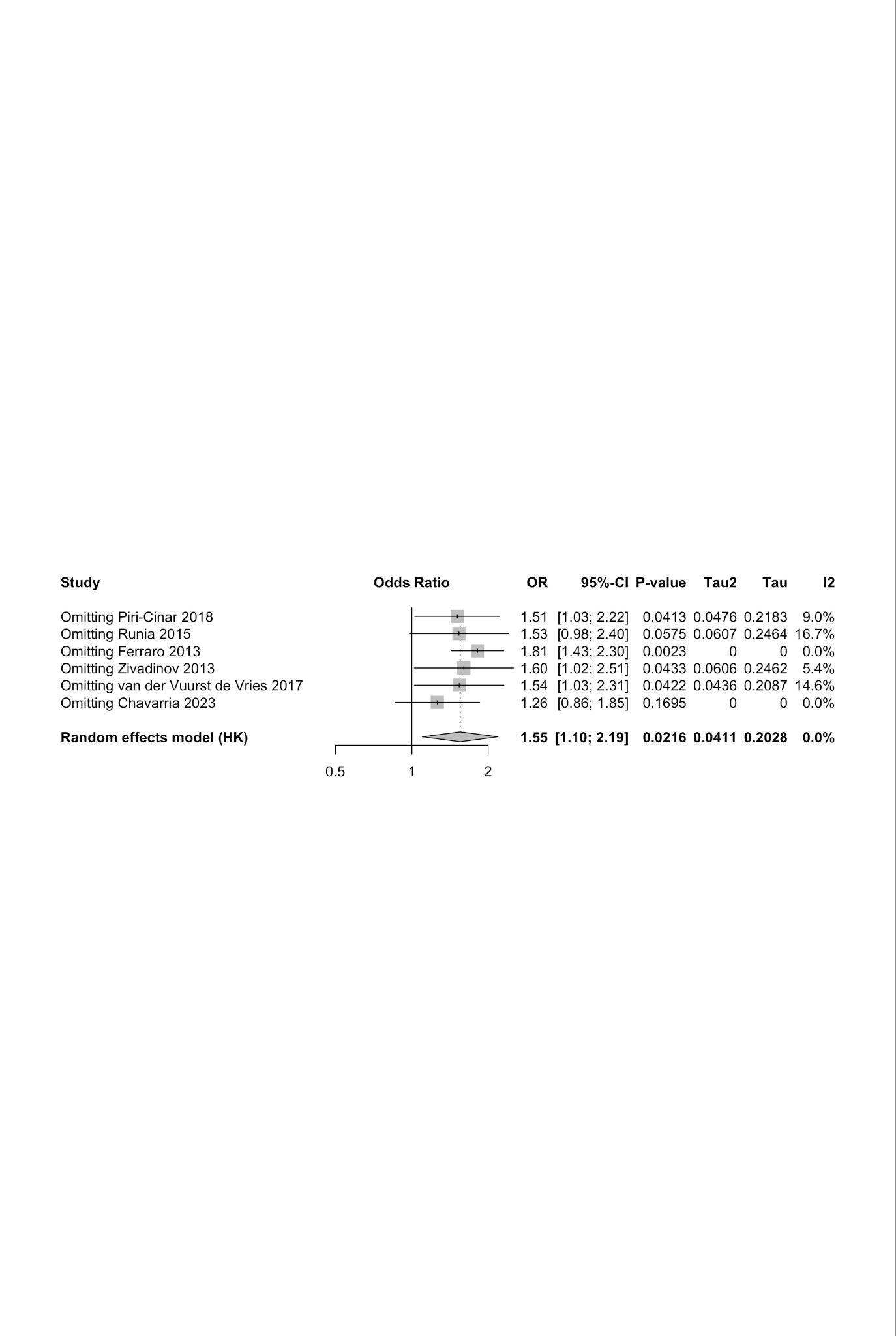
**

**eFigure 7. Funnel plot for multifocal presentation**

**
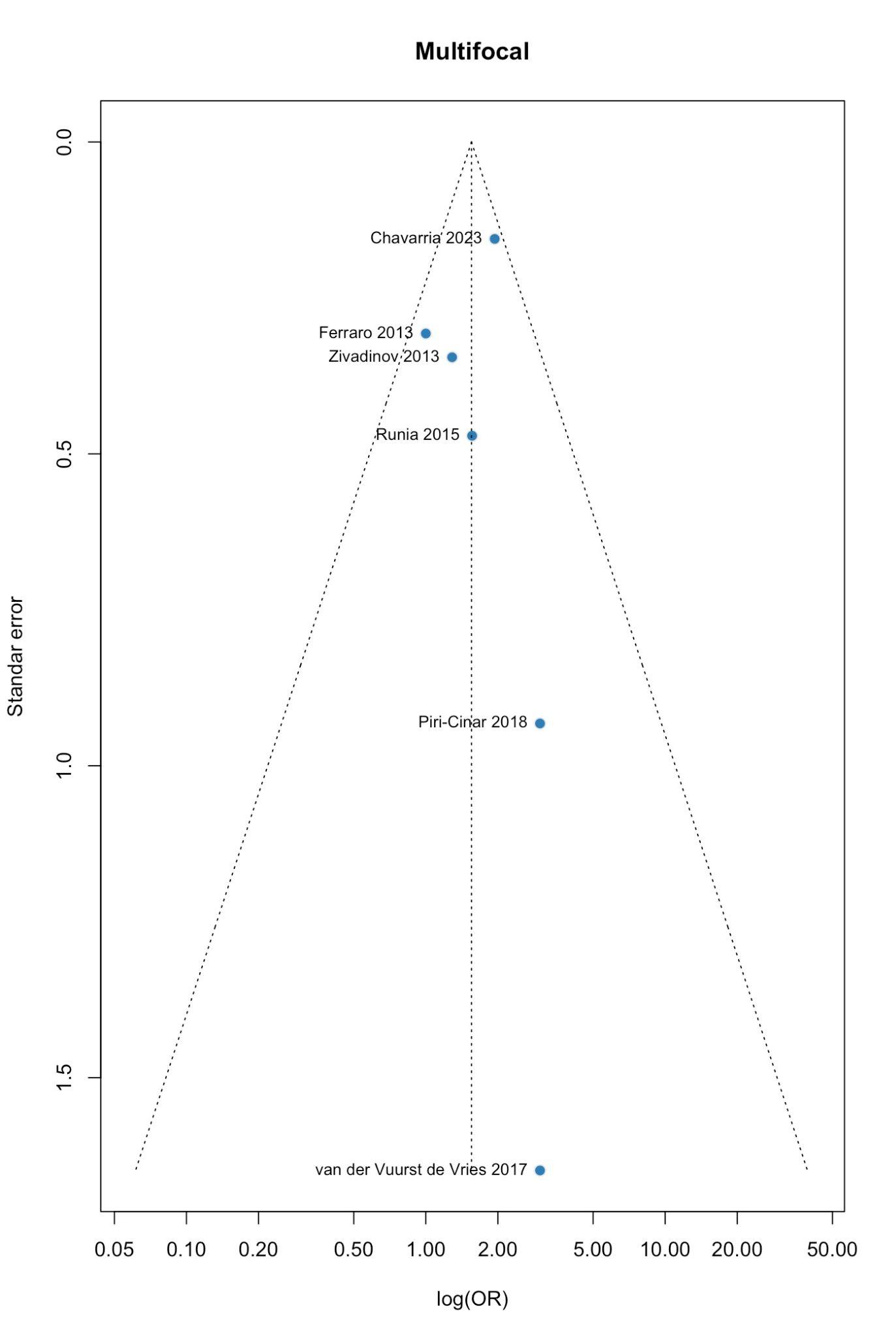
**

**eFigure 8. Forest-plot of leave-one-out analysis for optic neuritis and spinal cord**

1. **Optic neuritis**

**
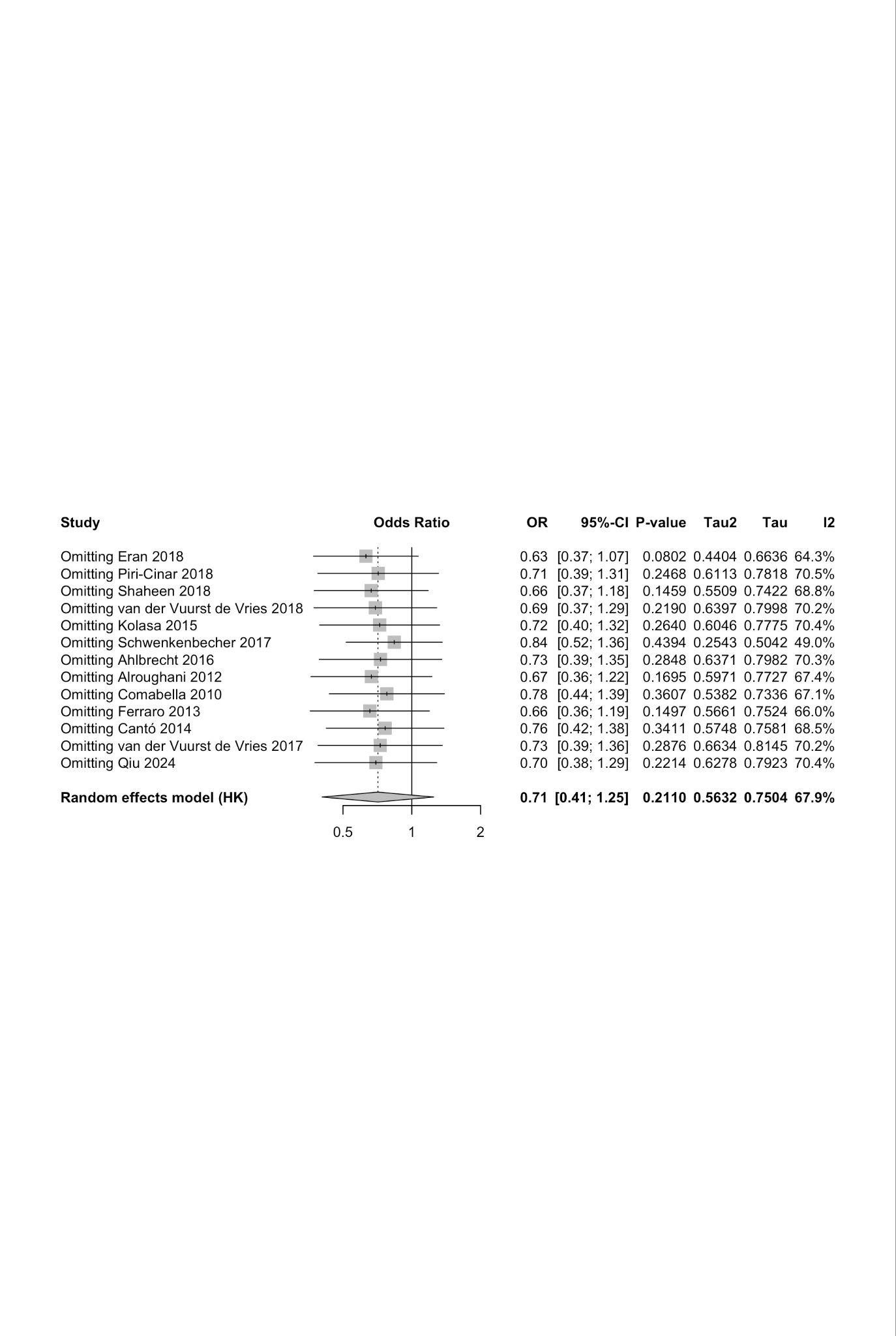
**

1. **Spinal cord**

**
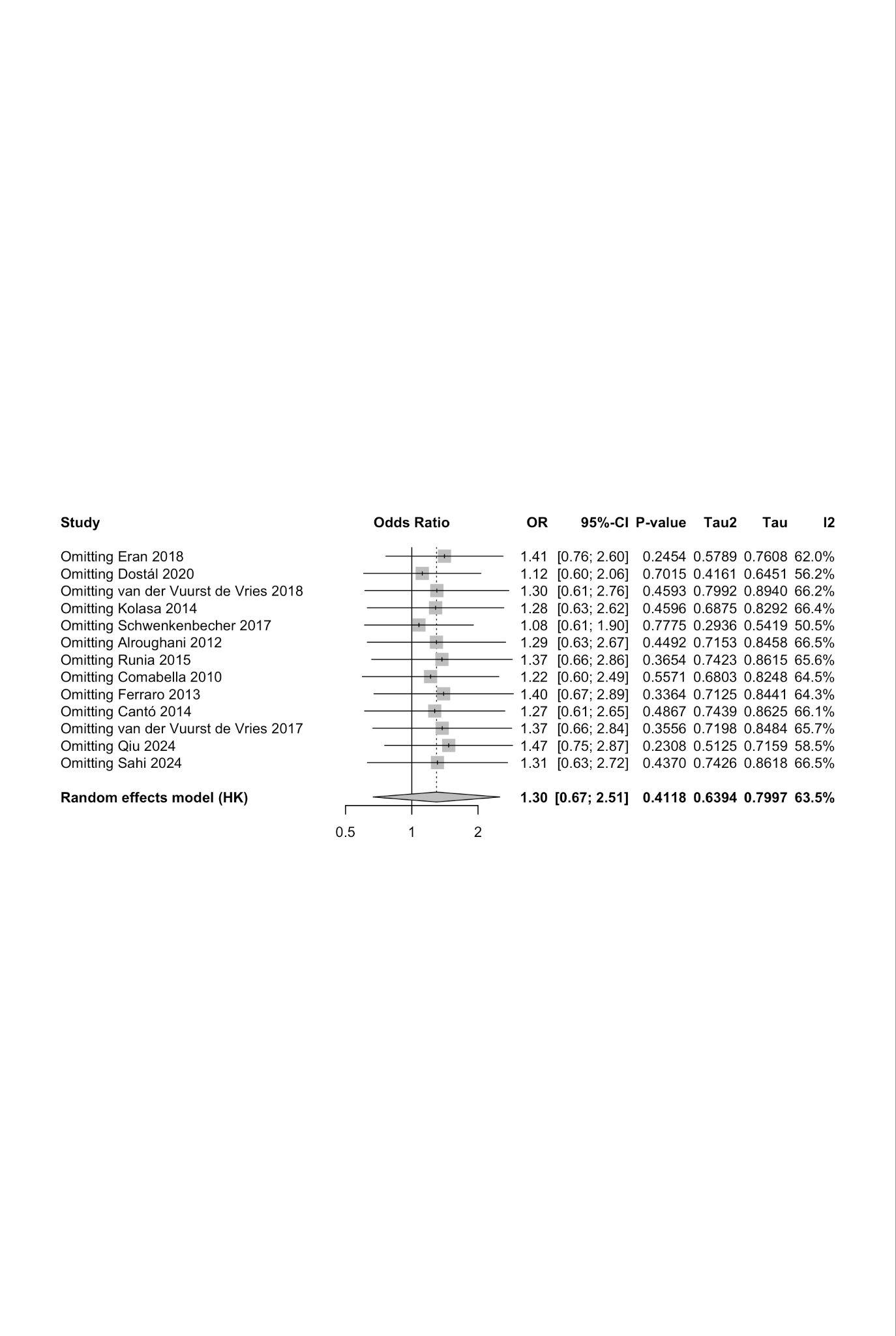
**

**eFigure 9. Funnel plot for optic neuritis and spinal cord**

1. **Optic neuritis**

**
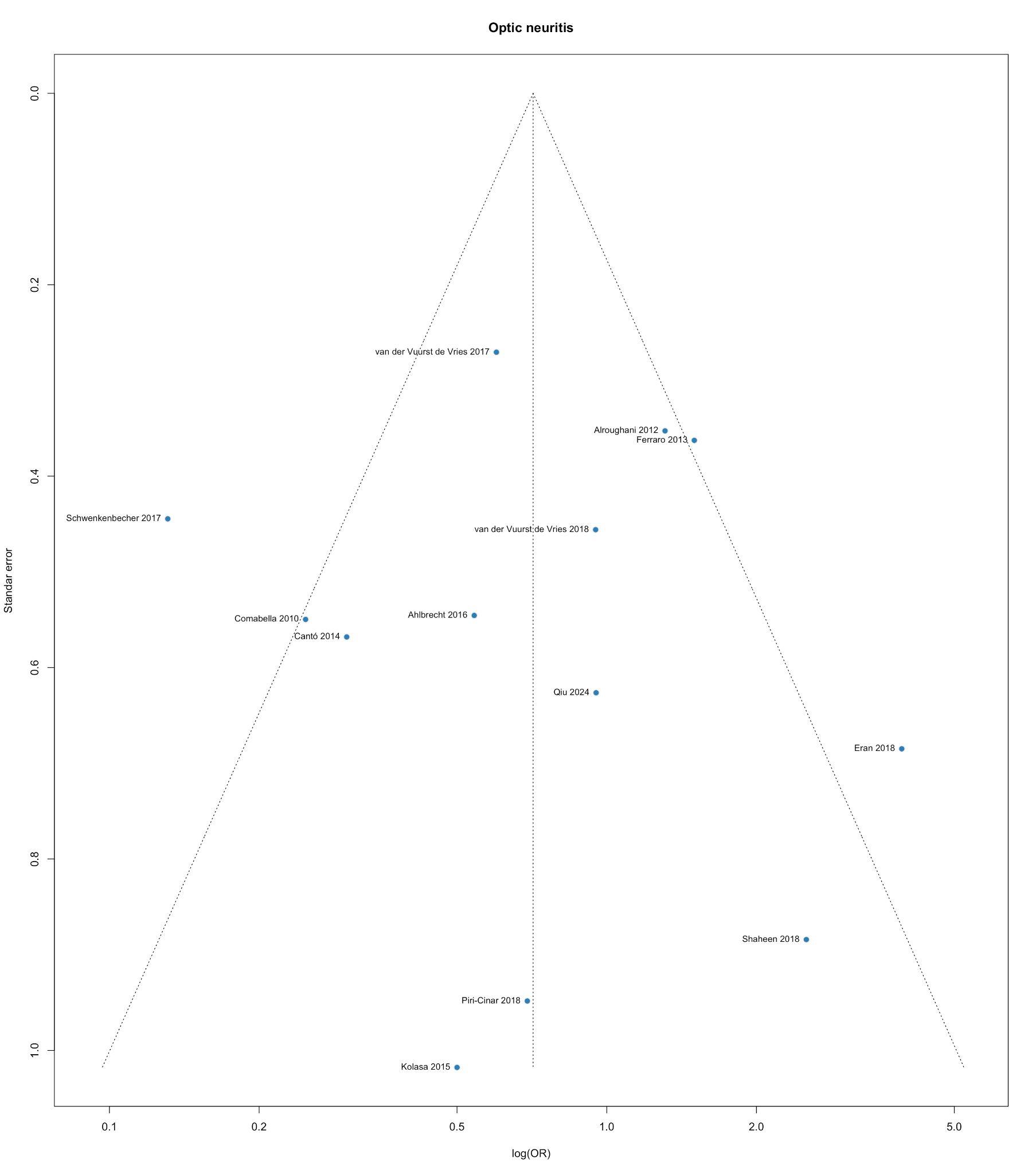
**

1. **Spinal cord**


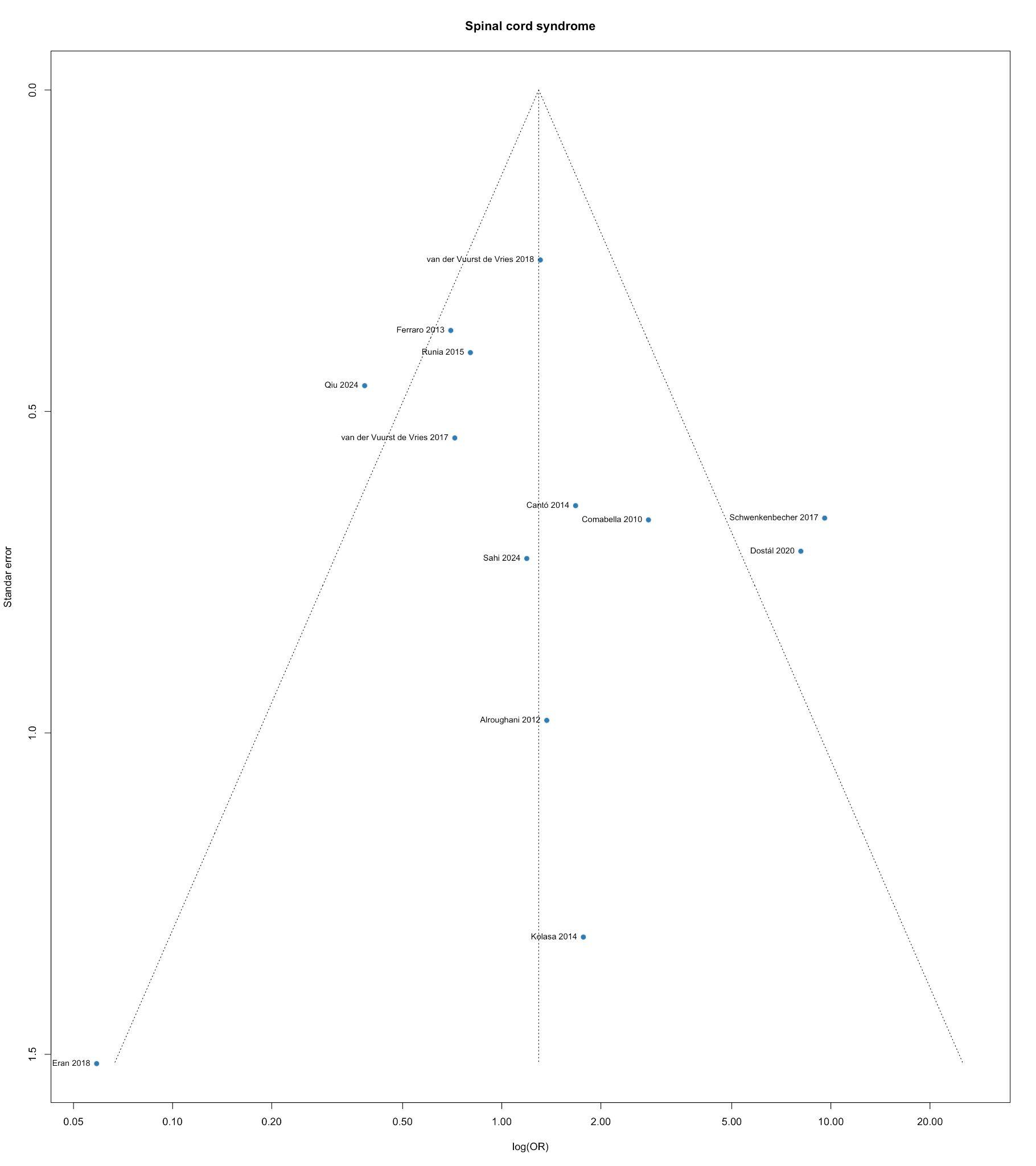


**eFigure 10. Forest-plot of leave-one-out analysis for number of T2 lesions**

**
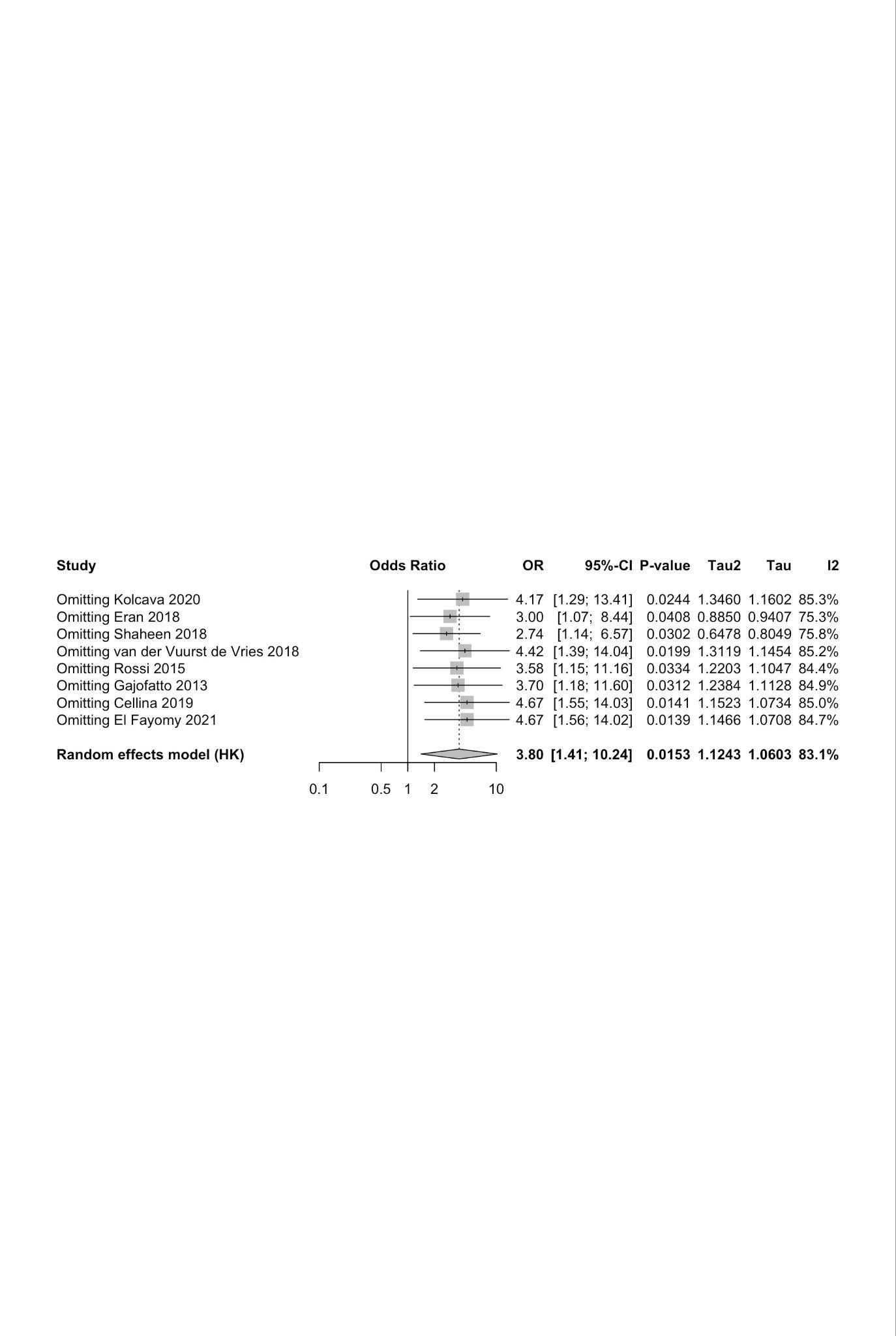
**

**eFigure 11. Funnel plot for number of T2 lesions**

**
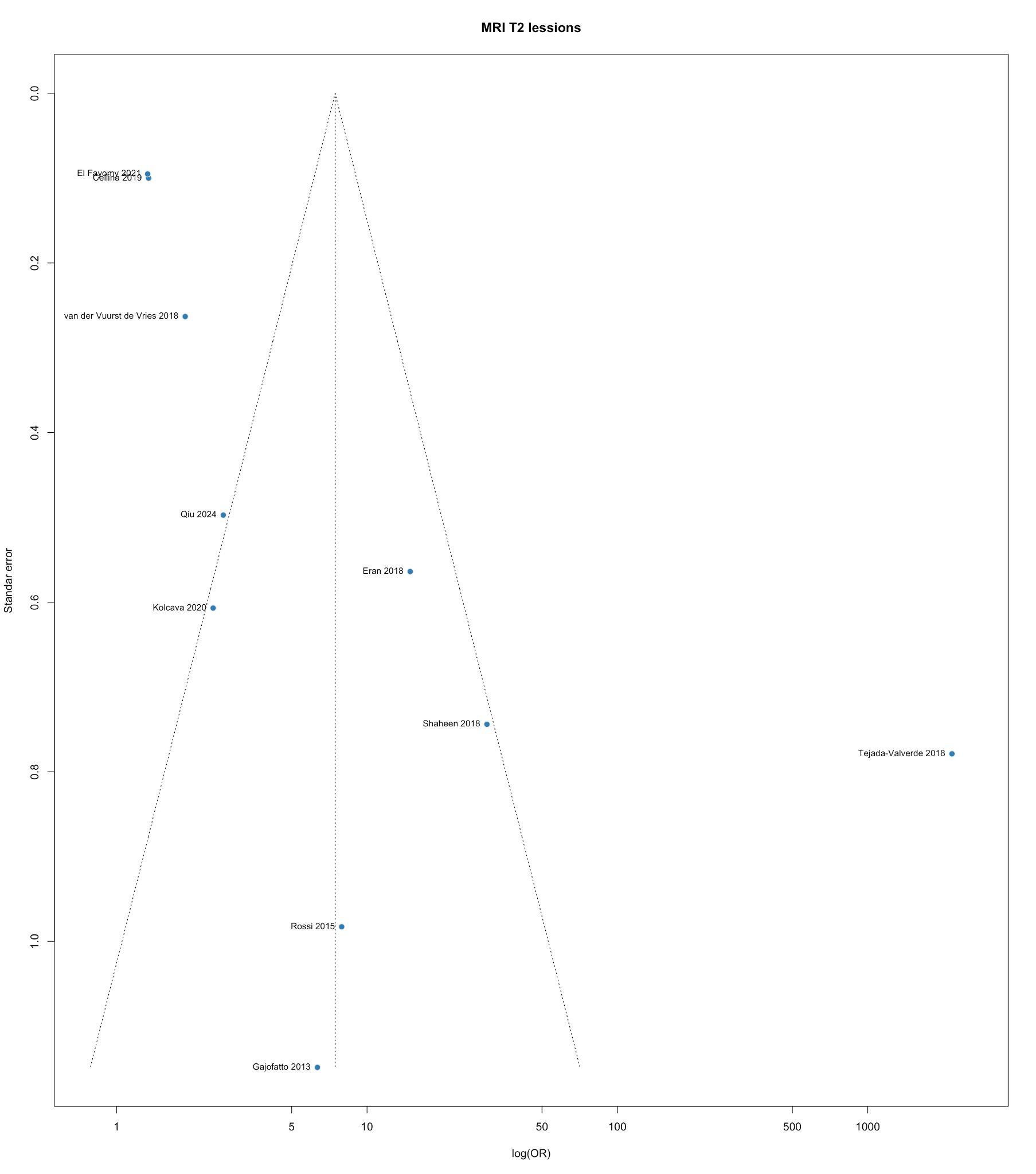
**

**eFigure 12. Forest-plot of leave-one-out analysis for lesion localization**

1. **Spinal cord**

**
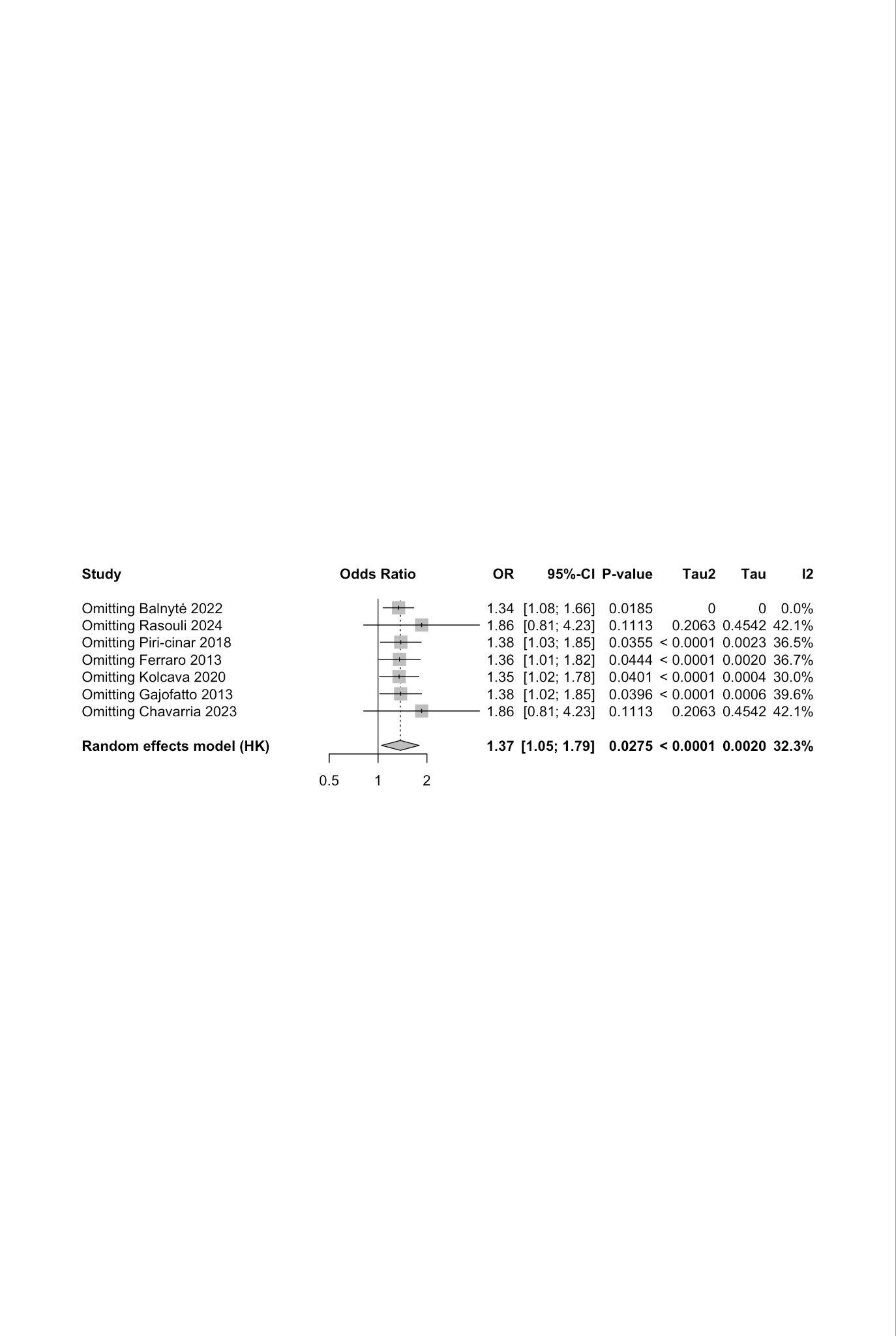
**

1. **Periventricular**

**
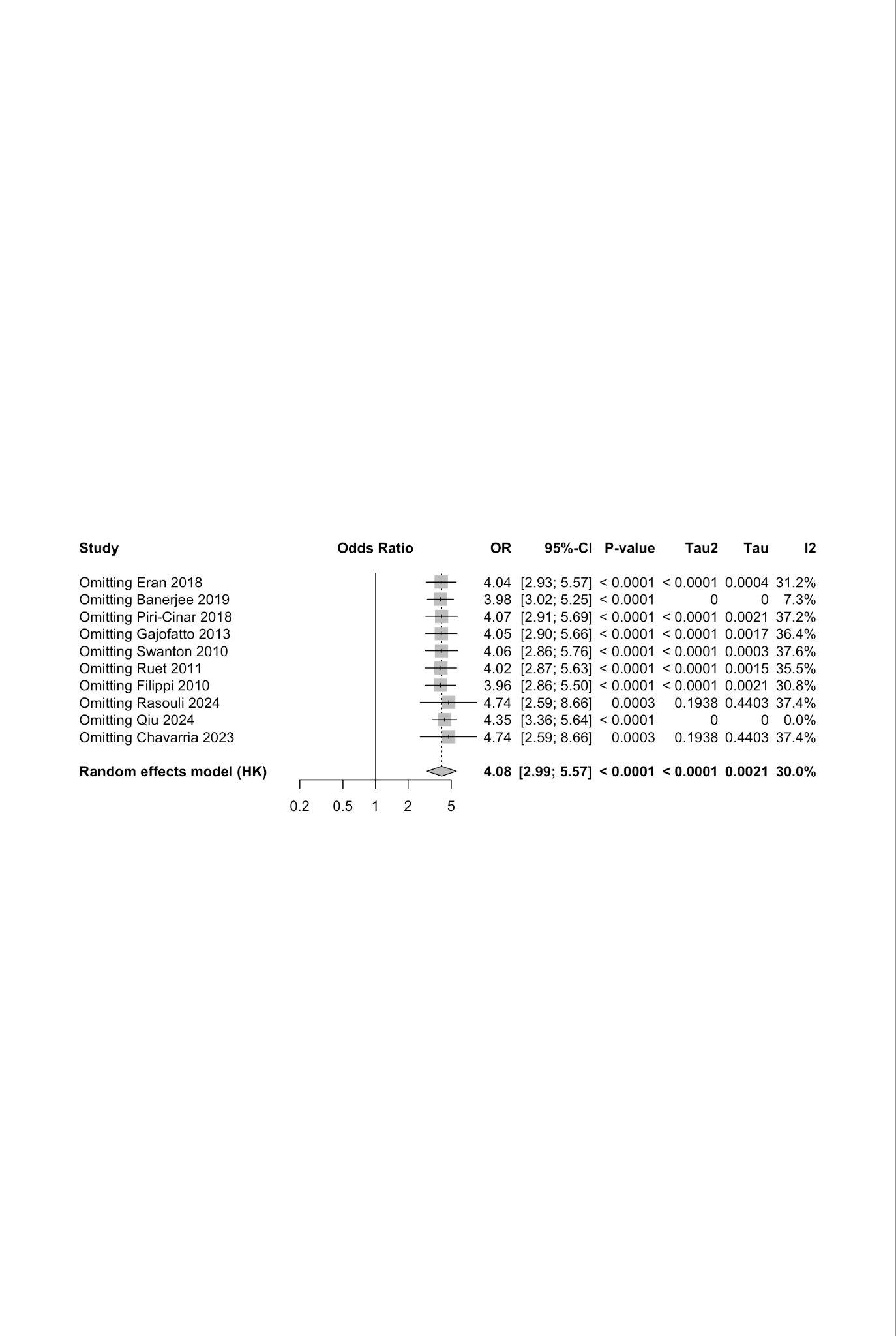
**

1. **Infratentorial**

**
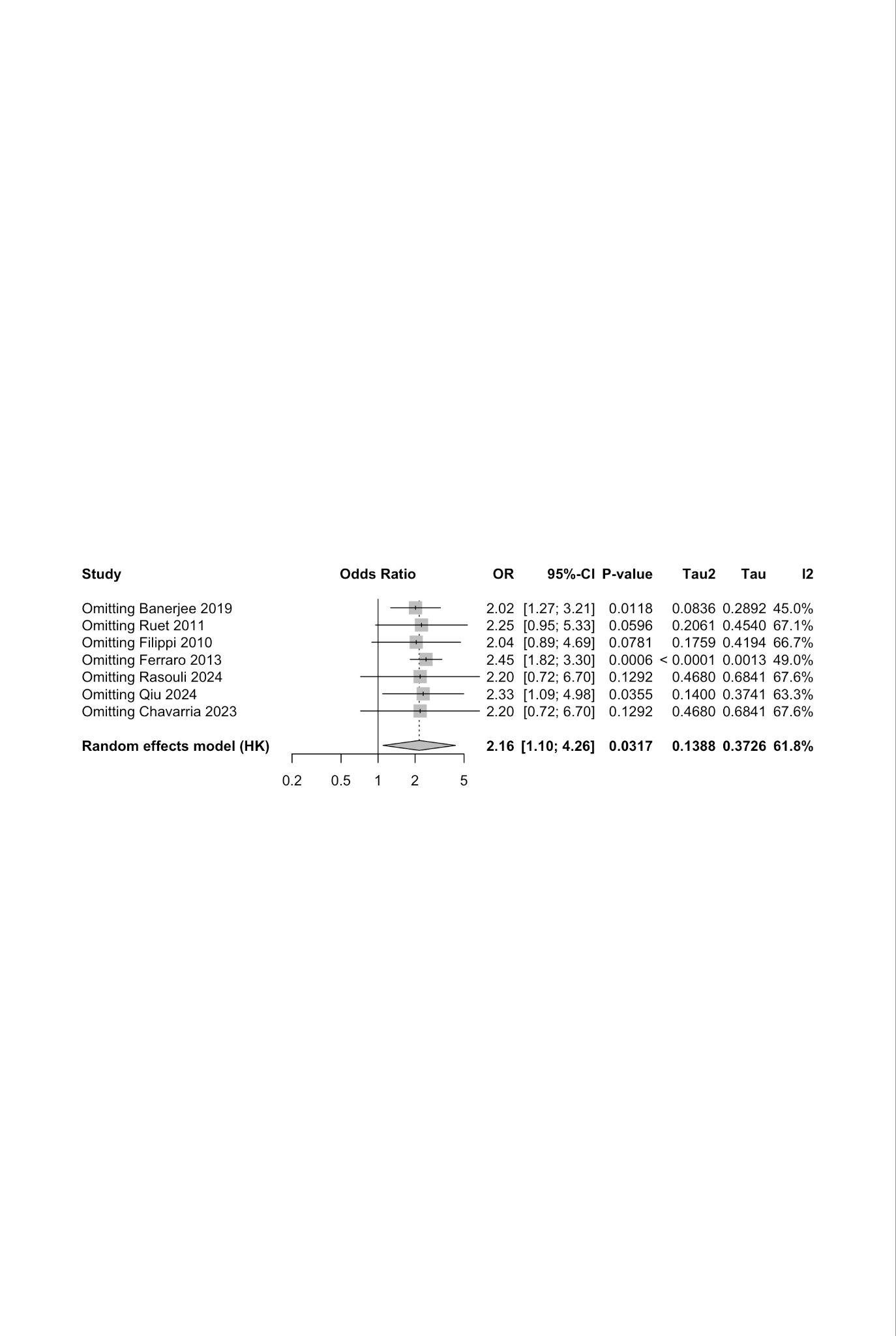
**

1. **Corpus callosum**

**
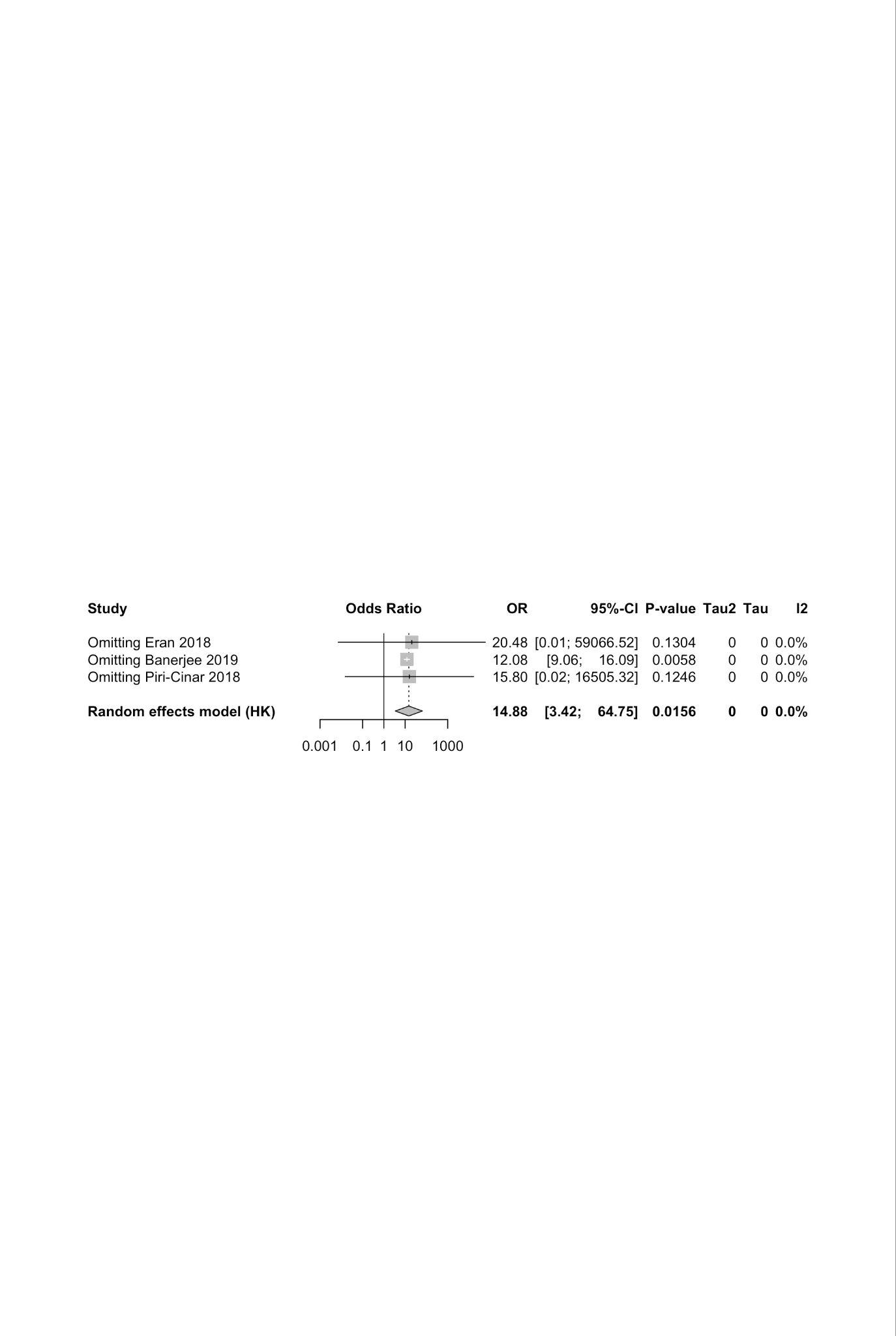
**

**eFigure 13. Funnel plot for lesion localization**

1. **Spinal cord**

**
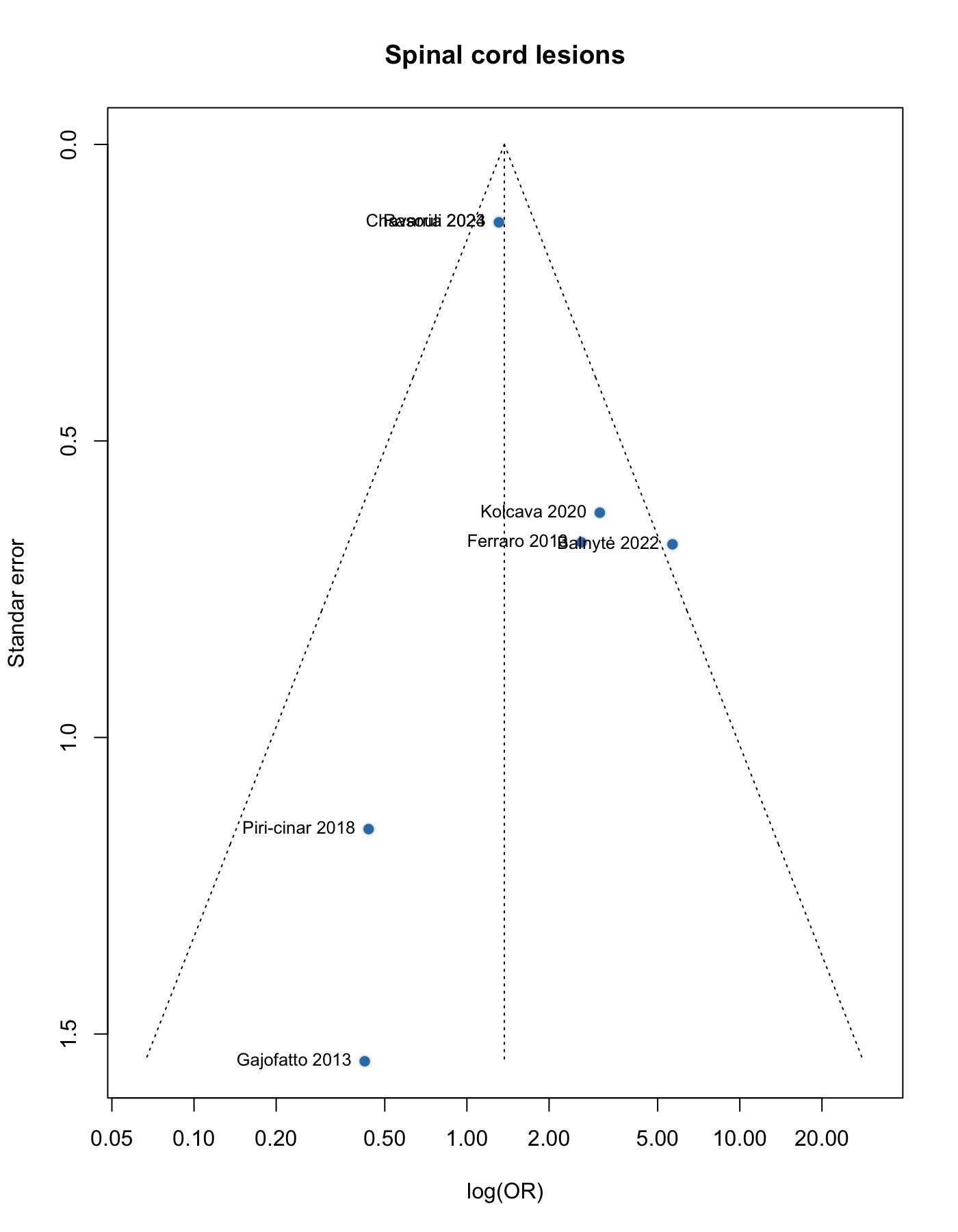
**

1. **Periventricular**


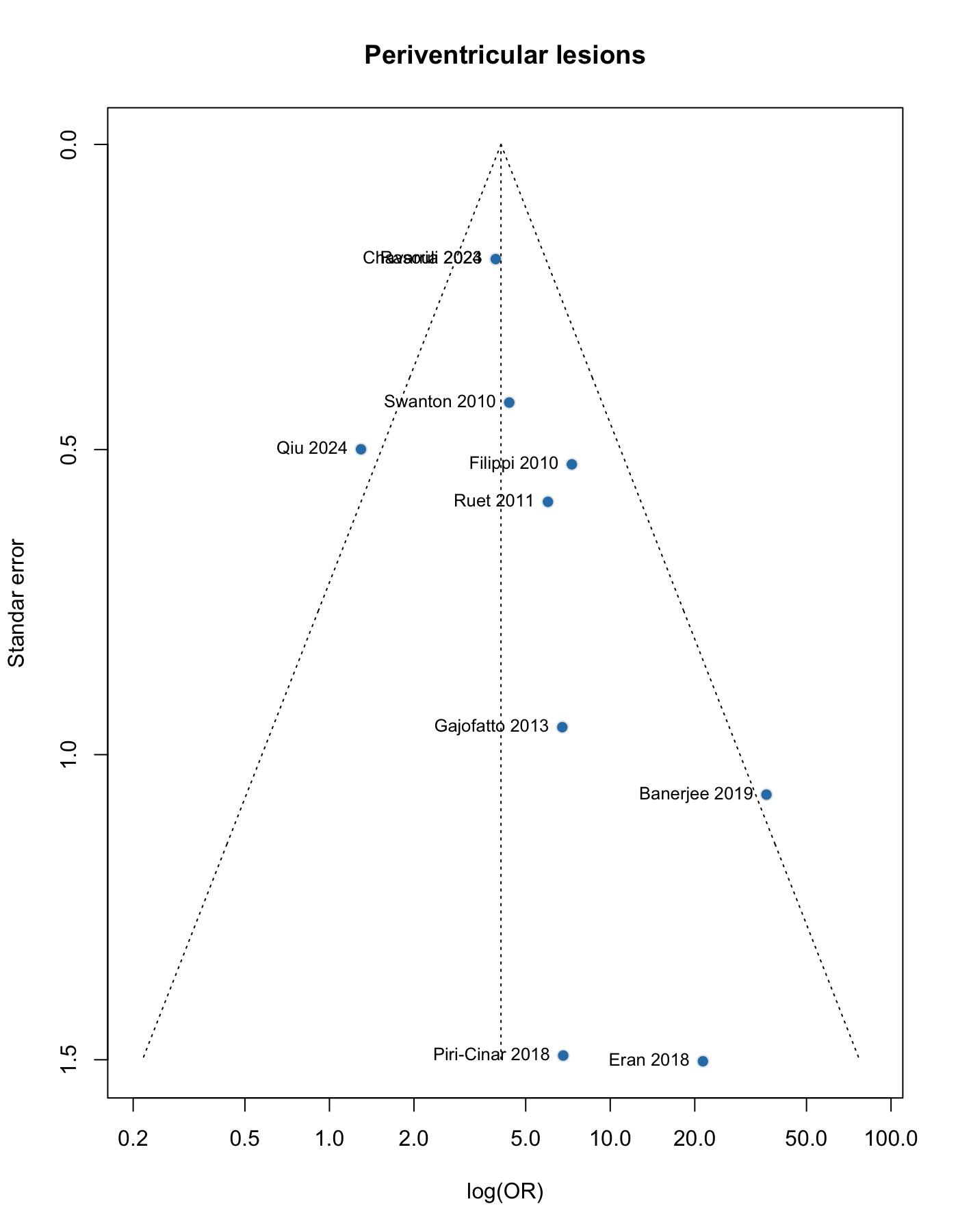


1. **Infratentorial**

**
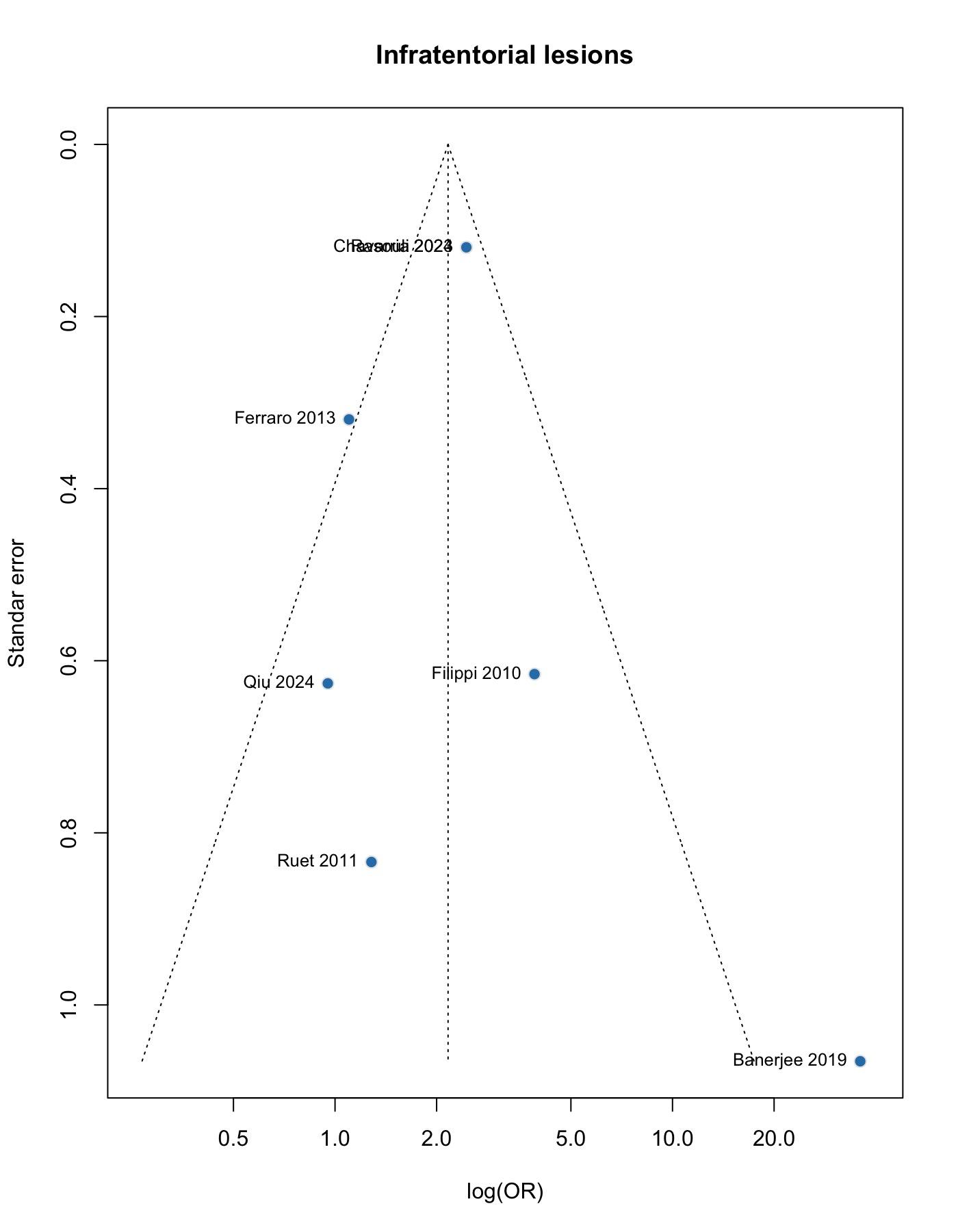
**

1. **Corpus callosum**


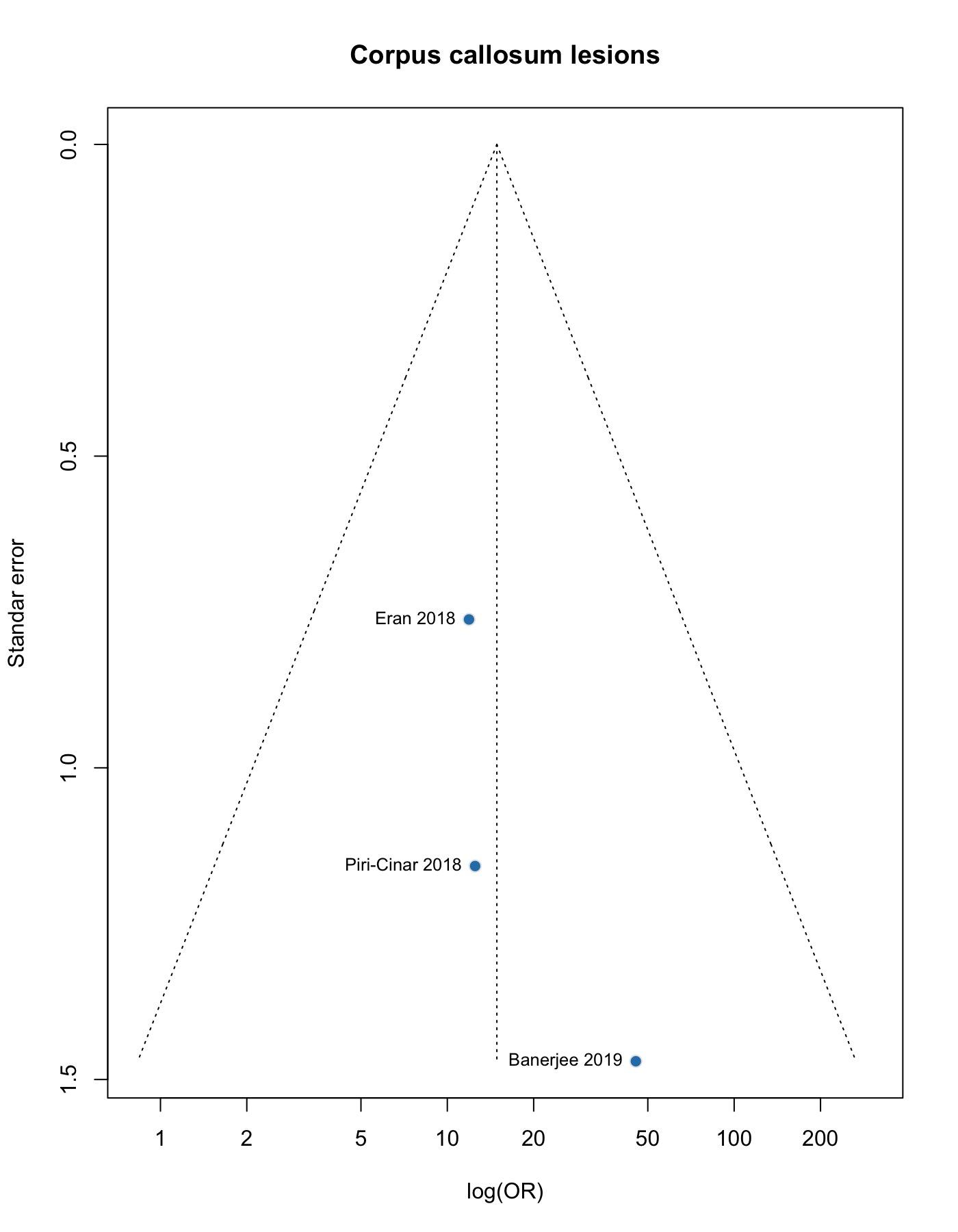


**eFigure 14. Forest-plot of leave-one-out analysis for gadolinium enhancement**

**
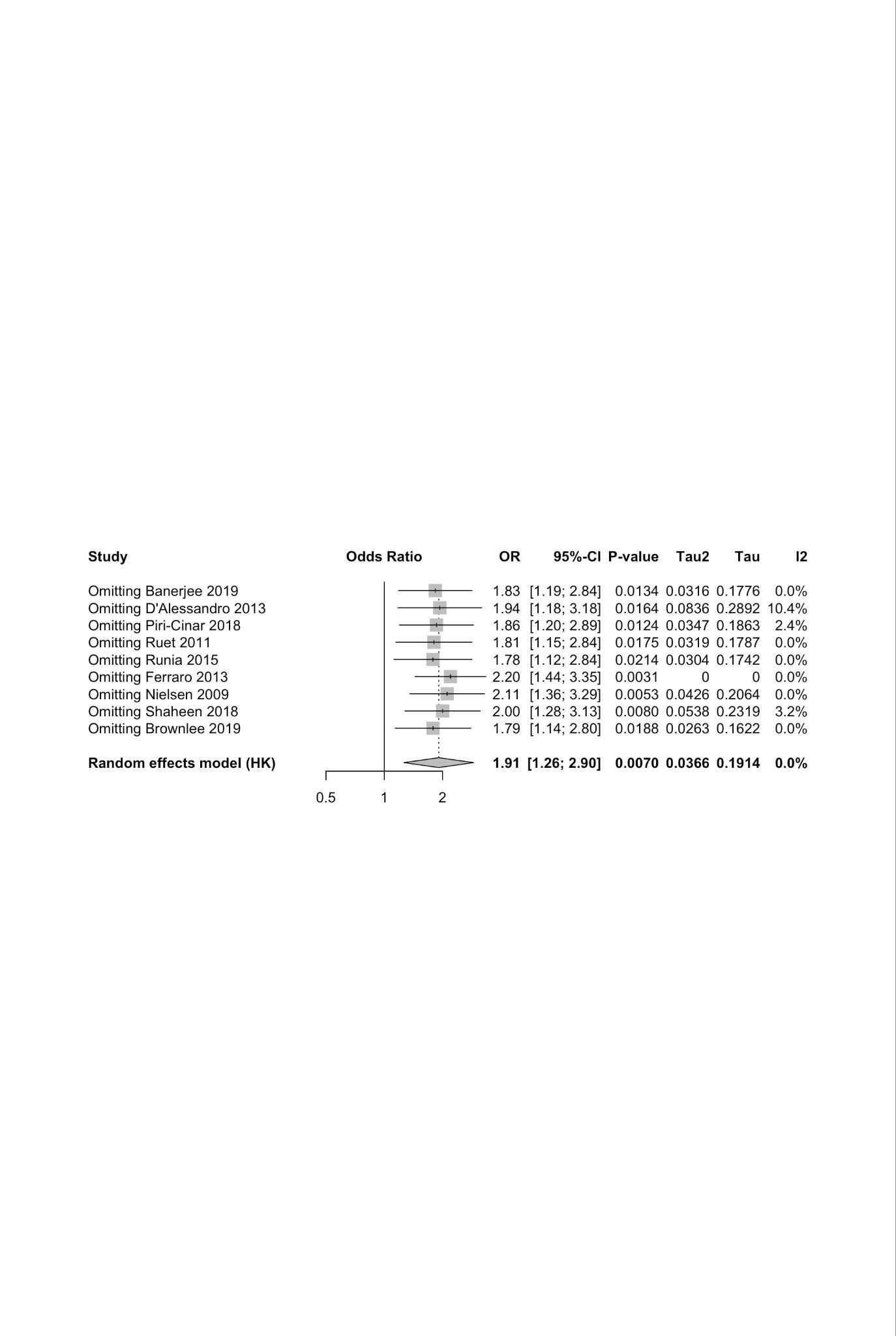
**

**eFigure 15. Funnel plot for gadolinium enhancement**

**
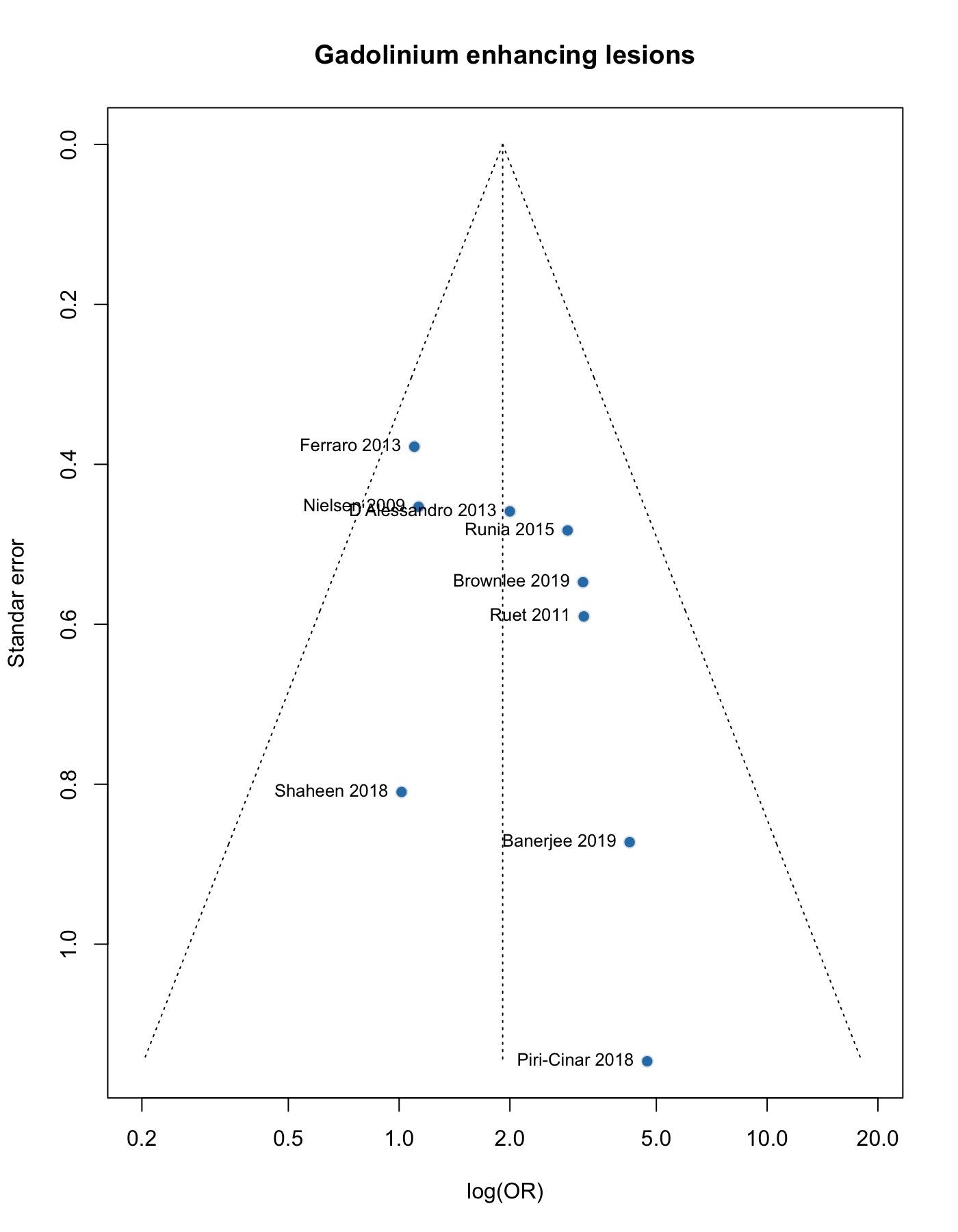
**

**eFigure 16. Forest-plot of leave-one-out analysis for oligoclonal bands**

**
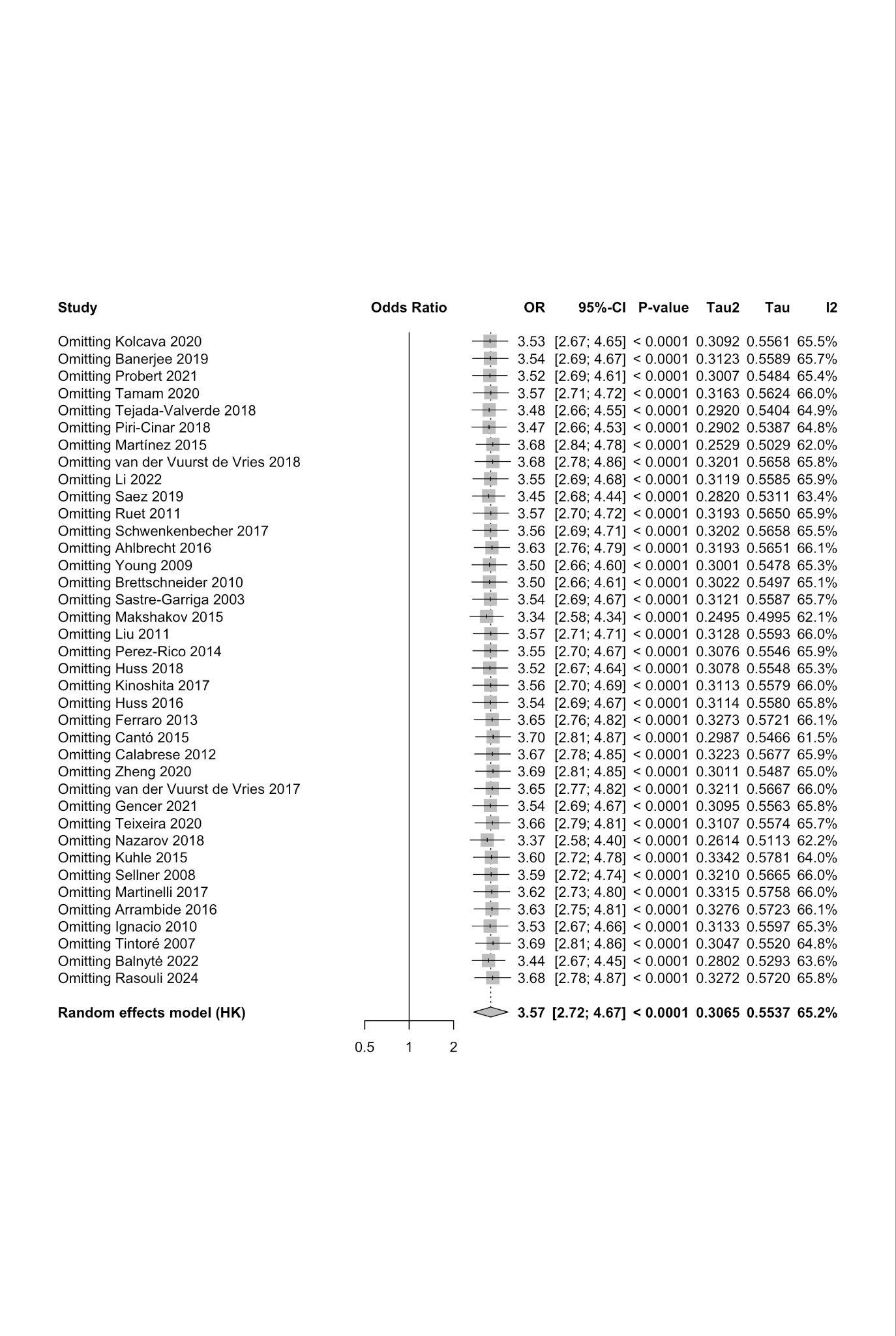
**

**eFigure 17. Funnel plot for oligoclonal bands**

**
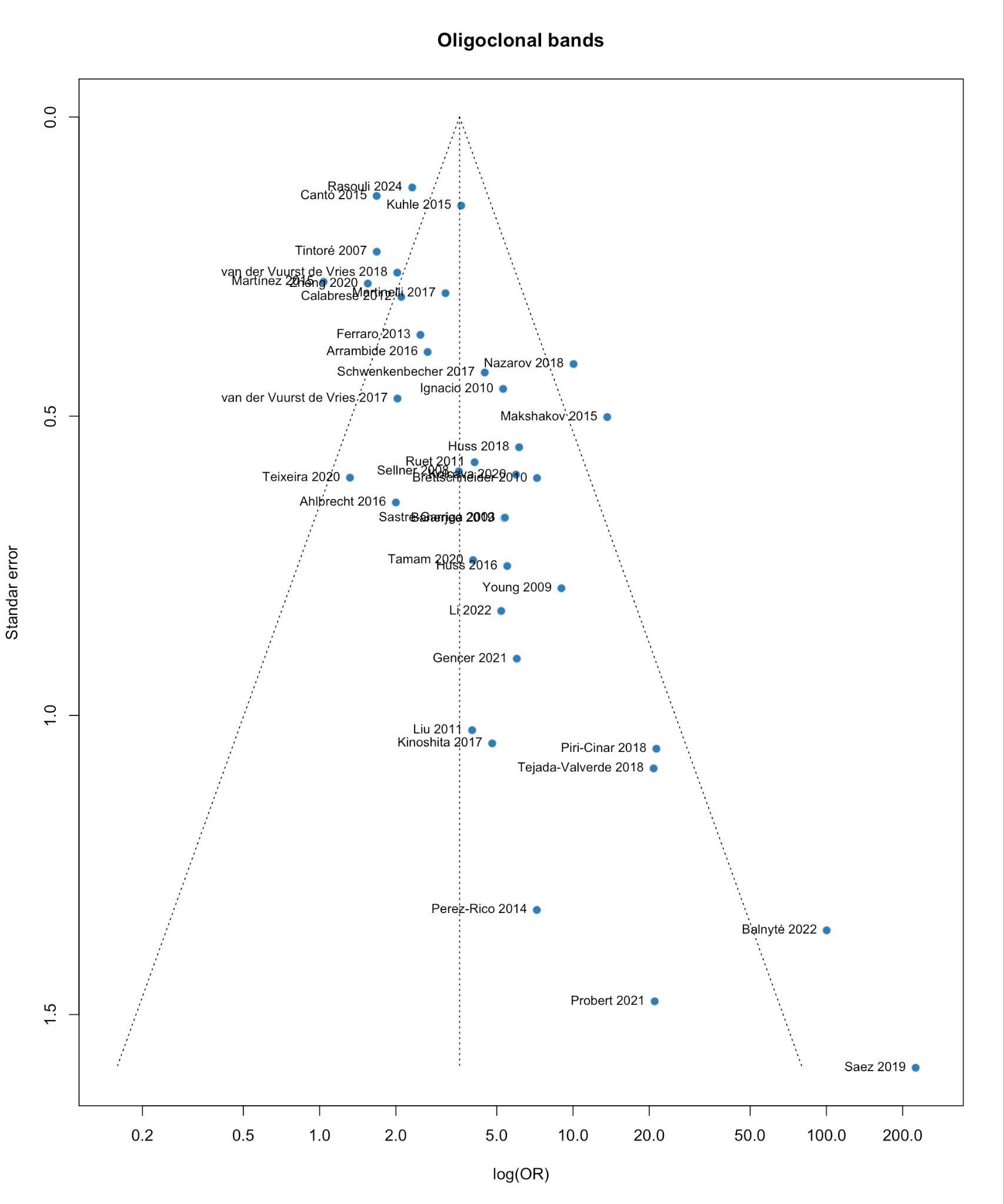
**

**eFigure 18. Forest-plot of leave-one-out analysis for CSF pleocytosis**

**
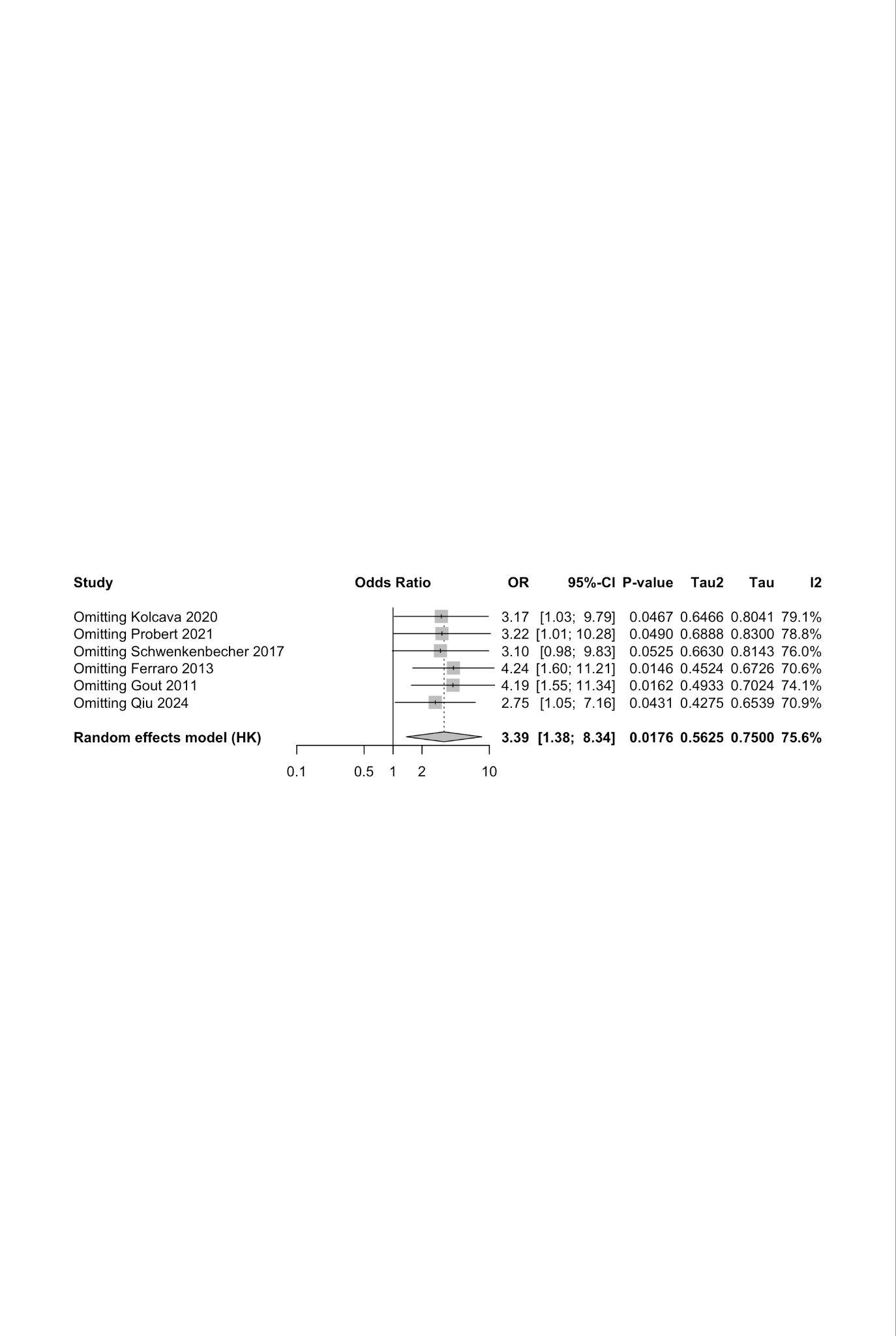
**

**eFigure 19. Funnel plot for CSF pleocytosis**

**
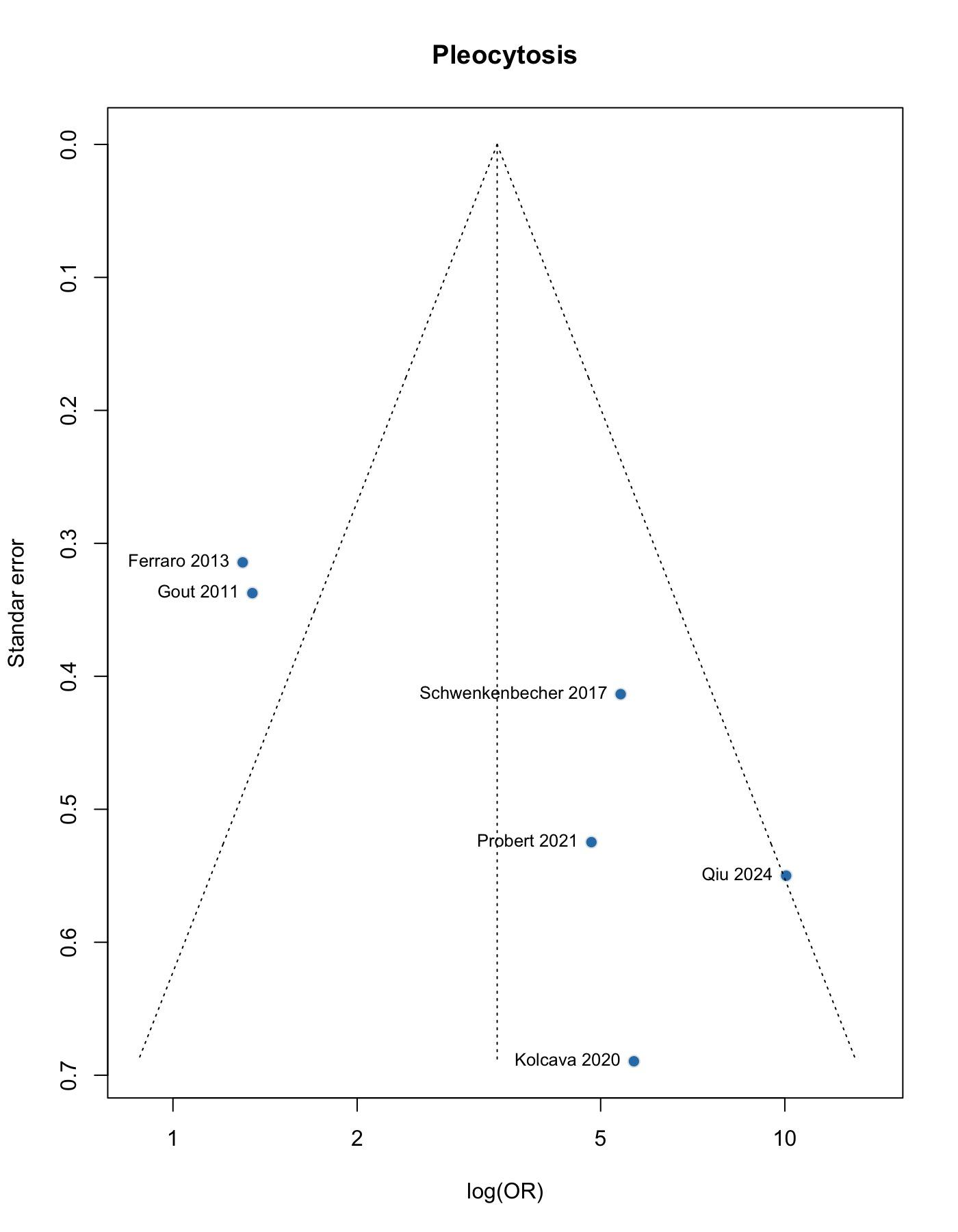
**

**eFigure 20. Forest-plot of leave-one-out analysis for serum vitamin D deficiency and CSF NFL**

1. **Serum vitamin D deficiency
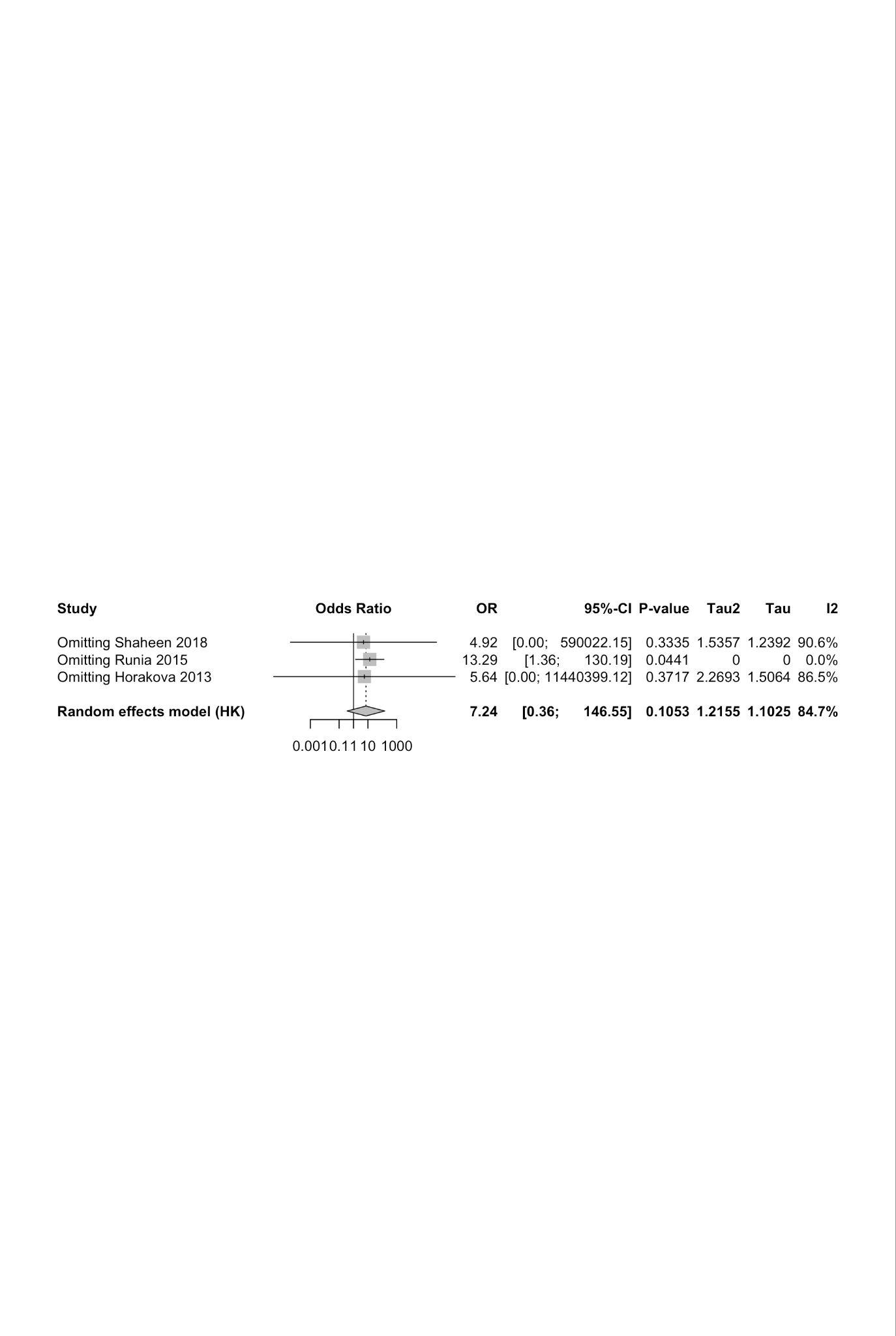
**
2. **CSF NFL levels**

**
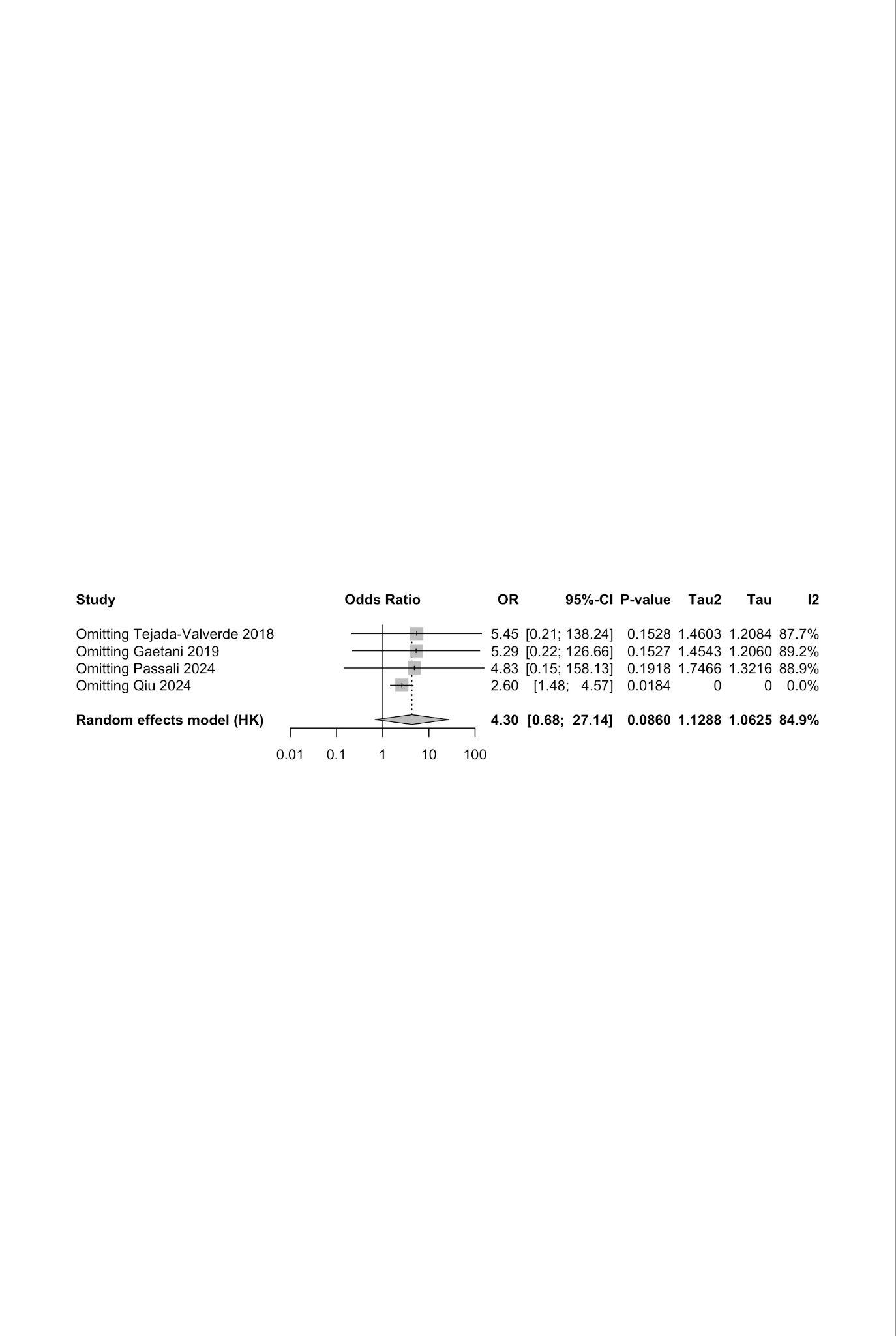
**
